# Are items from current self-report measures adequate for assessing autistic traits in women? Insights from a modified Delphi study with autistic women and professional experts

**DOI:** 10.1186/s13229-026-00724-1

**Published:** 2026-07-17

**Authors:** Nora Uglik-Marucha, Jude Fredman, Tala Al-Shurafa, Clarissa Ragrag, Francesca Happé, Hannah Belcher, Silia Vitoratou

**Affiliations:** 1 Psychometrics and Measurement Lab, Biostatistics and Health Informatics Department, Institute of Psychiatry, Psychology and Neuroscience, King’s College London, London, SE5 8AF UK; 2 Social, Genetic and Developmental Psychiatry Centre, Institute of Psychiatry, Psychology and Neuroscience, King’s College London, London, SE5 8AF UK; 3 Health Service and Population Research, Institute of Psychiatry, Psychology and Neuroscience, King’s College London, London, SE5 8AF UK

**Keywords:** Autism, Gender bias, Measurement, Psychometrics

## Abstract

**Background:**

Sex/gender-related measurement bias in autism measures has been proposed as a factor contributing to the underdiagnosis of autistic women, yet research in this area remains limited. This study builds on a small but growing body of work addressing this gap by using a modified Delphi methodology to reach consensus among autistic women and academics/clinicians on the most relevant items from five measures of autistic traits: Autism Spectrum Quotient (AQ-50), Broad Autism Phenotype Questionnaire (BAPQ), Comprehensive Autistic Trait Inventory (CATI), Girls Questionnaire for Autism Spectrum Condition (GQ-ASC), and Ritvo Autism and Asperger Diagnostic Scale-14 (RAADS-14), and to evaluate each measure’s suitability for autistic women in their current form.

**Methods:**

Thirty-three autistic women and thirty-three academics/clinicians participated in the study. Each participant was asked to rate the relevance of individual items to the experiences of autistic women and to indicate whether each measure comprehensively captured experiences relevant to this group. Agreement was calculated separately for each group using item-level content validity indices, from which scale-level indices were derived for each measure.

**Results:**

The RAADS-14 was rated as the most relevant measure for assessing autistic traits in women, followed by the CATI, while the remaining questionnaires were associated with lower perceived relevance. However, none fully captured experiences relevant to autistic women, indicating construct underrepresentation. Items assessing masking were identified as valuable for inclusion to enhance the relevance of measures for women, whereas items related to attention to detail and imagination appeared less useful for assessing autism more broadly or for capturing more nuanced presentations of autistic traits.

**Limitations:**

The sample was predominantly Western, White, and highly educated, limiting the generalisability of findings. Furthermore, the expert groups lacked sufficient homogeneity, as academics and clinicians were combined and participants evaluated different subsets of measures.

**Conclusions:**

Although the RAADS-14 and CATI were identified as the most relevant measures for autistic women, all instruments demonstrated incomplete coverage of the autism construct in this population. Items reaching consensus as most relevant across the five measures provide a foundation for refining existing scales to better capture the experiences of individuals with more nuanced presentations of autistic traits.

**Supplementary Information:**

The online version contains supplementary material available at https://doi.org/10.1186/s13229-026-00724-1.

## Introduction

Over recent decades, understandings of autism spectrum condition (hereafter, ‘autism’) have undergone a substantial shift, particularly in relation to the different ways in which it can present [1]. This reconceptualisation was formalised in the 5th Edition of the Diagnostic and Statistical Manual of Mental Disorders (DSM-5) [2], which broadened the diagnostic criteria and adopted a spectrum approach to describe autism. Under this approach, the heterogeneity of autism is unified under differences in social communication alongside restricted and repetitive patterns of behaviours and interests (RRBIs) [3]. These changes have prompted efforts to identify a ‘lost generation’ of autistic adults who may have gone undiagnosed under earlier criteria [4]. In the United Kingdom (UK) alone, underdiagnosis remains a significant concern, particularly among adults, with estimates indicating that approximately 52.54%, 89.29% and 96.51% of young, middle-aged and older autistic adults, respectively, may remain undiagnosed [5].

Despite evolving conceptualisations of autism and changes to diagnostic criteria, an enduring characteristic has been the higher prevalence of autism diagnoses among males than females. This pattern has likely been shaped by early autism descriptions based predominantly on boys, which, unlike earlier accounts including both boys and girls [6], gained international prominence and influenced the nosological framing of autism. In the past several years, different male-to-female ratios, ranging from approximately 2–4:1, have been reported [7–10]. Variation in these estimates is partly attributable to methodological differences: population-based screening irrespective of prior diagnosis (active ascertainment) tends to yield lower ratios (3.25:1) than approaches relying on existing diagnostic records (passive ascertainment; 4.20:1) [9]. When more recent data are used, which capture increased diagnoses of autism in females, particularly later in life [11, 12], studies employing active methods tend to report even lower ratios (2.1:1) [10]. Differences in rates across case-ascertainment approaches suggest the possibility of bias in females’ access to, or obtaining of, an autism diagnosis. This disparity may, however, be gradually narrowing, with very recent data indicating that sex/gender differences in prevalence may be even less perceptible than previously assumed. A population-based study from Sweden reported that by age 20 in 2022, the sex/gender ratio was estimated at 1.2:1, with projections suggesting that this ratio in prevalence may become comparable by adulthood [13]. Although, reassuringly, diagnoses among females have increased in recent years, females are still often referred [14] and diagnosed later in life than males [11, 13], and underdiagnosis still remains substantial, despite ongoing concerns regarding overdiagnosis [15]. Around 18.13% of autistic girls and 49.27%, 79.48% and 97.19% of young, middle-aged and older autistic women, respectively, are estimated to remain undiagnosed in the UK. As these estimates were computed under the assumption of a 3:1 male-to-female ratio [5], they may therefore underestimate the true extent of underdiagnosis if the true ratio is lower [5], as suggested by more recent studies [10, 13]. While the disparity in ratios may reflect genuine sex/gender^1^ differences, it may also be partially attributable to systemic biases in identification of females, as indicated by discrepancies across case-ascertainment approaches, that function and interact across multiple interpersonal and structural levels. One proposed source of such bias lies in the measurement of autistic traits in screening, assessment and research [16].

Historically, autism has been conceptualised as a predominantly male condition, and earlier measures, which are still frequently used today, were therefore developed in line with this understanding of autistic traits. Growing literature, however, indicates that autistic traits in women and girls may present in more nuanced ways [17], which existing measures may not adequately capture, thereby potentially contributing to their under-identification [16]. In this work, we therefore focus on these more nuanced presentations in autistic women, using gender as an inherently imperfect proxy for variation in autistic trait presentation, reflecting aggregate-level sex/gender differences. It is important to note, however, that autistic trait expression is heterogeneous at the individual level: people of any sex/gender may exhibit more nuanced, classical, or mixed presentations, and these should not be considered exclusive to any particular group, even if they may occur on average more frequently in one than in others.

Findings on sex/gender differences in autism are complex, and evidence from systematic reviews and meta-analyses of the core domains appears inconsistent. Some report no sex/gender differences in social communication [18–20], others suggest that autistic females demonstrate better social communication skills than males [21], and one found differences in social interaction but not in communication [22]. Results for RBBIs are similarly mixed. Some meta-analyses indicate that autistic females display fewer RRBIs than males [18, 19], or that males show higher levels of stereotyped behaviours and restricted interests, with no differences for insistence on sameness or sensory experiences [23]. Another meta-analysis, however, reports no sex/gender differences in this domain [22]. It has been suggested that sex/gender differences may be most evident at the level of subdomains within the core domains of autism (e.g., differences in social-emotional reciprocity within the social communication domain) [16], though only two meta-analyses have examined this. These studies reported higher levels of stereotyped behaviours and restricted interests in males, with no differences in insistence on sameness or sensory experiences [23], and better social communication skills in females [21]. Indeed, further research focusing on this narrow-construct level is needed to clarify sex/gender differences in how autism can present, particularly in adulthood. Existing reviews and meta-analyses predominantly focus on children and adolescents [18, 20, 21] or combine age groups [19, 22, 23], leaving sex/gender differences in core domains and subdomains among autistic adults underexplored, with no meta-analytic synthesis to date focused exclusively on adulthood.

Given growing evidence of these differences, particularly at the subdomain level, it is essential that existing measures capture more nuanced presentations of autistic traits often seen in women and girls, as overlooking these nuances may lead to missed opportunities for identification during screening. Indeed, the possibility of a ‘leaky pipeline’ in the assessment of autistic females has been raised, with a potential point of under-recognition at the level of screening [24]. If autistic women and girls are systematically missed at this stage, it may affect referral for diagnostic assessment, which often serves as an essential gateway to receiving specialised interventions and appropriate support [25]. Earlier identification, in turn, facilitates earlier access to these resources, which can significantly improve developmental outcomes and quality of life for autistic individuals [26]. Timely identification is particularly important for women and girls as autistic females tend to experience poorer mental health than autistic males [27], and may therefore benefit substantially from appropriate support. This disparity may reflect multiple interacting factors, including, but not limited to, delays in receiving autism diagnosis and sex-specific biological differences [27], but also a distinct genetic and developmental profile associated with later diagnoses, which is characterised by higher rates of co-occurring mental health conditions [28]. A large population-based study found that although psychiatric disorders and hospitalisations are common among autistic young adults, autistic females were more likely than males to experience them, particularly in relation to anxiety, sleep, and depressive disorders [29]. Another study highlighted the role of diagnostic timing, showing that in clinical (but not research) settings, females were diagnosed later than males, and this delay partly explained their higher levels of anxious and depressive symptoms [30]. Further, in a cohort of autistic women, those diagnosed in adulthood were more likely to have co-occurring anxiety, depression, eating disorders, and substance use diagnoses than those diagnosed in childhood [31]. Mental disorders are recognised contributors to suicide risk [32]. Globally, in 2021, autistic people were nearly three times more likely to die by suicide than the general population, with autistic females showing a significantly higher relative risk for suicide than autistic males [33]. These estimates are particularly concerning given the high rates of undiagnosed autistic women, who may consequently lack access to appropriate support. However, it needs to be acknowledged that access to such support still remains poor for autistic people more generally, with the improvement of mental health support in this population identified as a key priority [34]. Together, these findings highlight the pressing need to investigate potential sex/gender bias in measurement tools that could facilitate more timely identification of autism in women and girls and, consequently, their access to the associated benefits of diagnosis, including professional support and beneficial impact on sense of self [35].

If existing measures assess autistic traits differently based on sex/gender alone, this would indicate sex/gender-related measurement bias that can undermine their validity for measuring autism. In research, such bias can lead to systematic over- or underestimation of scores for a particular sex/gender [36, 37], leading not only to invalid between-sex/gender comparisons but also to biased sample selection (e.g., differential inclusion in autism groups) and misclassification into trait-based strata (e.g., low vs. high). Consequently, the resulting evidence base may be distorted, with downstream effects on theoretical models and clinical practices that inadvertently reinforce sex/gender-related biases.

Although the literature on the identification of autistic women has raised the possibility of sex/gender-related measurement bias in autism measures, research in this area remains limited. A small but growing body of research has applied psychometric methods to investigate potential sex/gender-related measurement bias in self-report autism measures for adults, using investigations of measurement invariance and differential item functioning. The present work focuses on self-report tools to reflect their widespread use with adults amid the substantial underdiagnosis of autism in adulthood [5], while also addressing the historical preference for informant reports and assessments of observable behaviours [38], presumably rooted in autism’s early conceptualisation as a childhood condition and assumptions about autistic people’s capacity for accurate self-reflection [39].

One measure that has been the focus of sex/gender-related measurement invariance investigations is the Autism Spectrum Quotient (AQ-50) [40], first published in 2001 under the DSM-IV criteria [41]. It remains among the most widely known and commonly used measures of autistic traits in research, as reflected by over 8,000 citations on Google Scholar, which is also recommended by the National Institute for Health and Care Excellence (NICE) [42] as part of a broader assessment battery for autism. While preliminary findings suggest the possible presence of sex/gender-related bias of this measure in English-speaking contexts, both at the level of the basic factor structure [43] and at the item level [44], the magnitude of the bias and the extent to which it affects screening performance remain unclear, making it necessary to exercise caution in drawing firm conclusions. Greater attention, however, has been given to the brief version of the AQ-50, the Autism Spectrum Quotient-10 (AQ-10) [45], which similarly has substantial clinical relevance, having been recommended by NICE for autism screening, while also serving as a widely used instrument in research contexts (>1000 citations on Google Scholar). Similar to its longer version, studies conducted in primarily English-speaking contexts have reported possible sex/gender-related bias in the AQ-10 (analysed under one factor model) both at the level of the basic factor structure [43] and at the item-level [44, 46, 47]. Importantly, however, two of these studies from the same research team reported that despite evidence of item-level bias, no significant bias was observed at either the total score level or at the latent trait level corresponding to the clinical cut-off point [46, 47]. Other frequently used measures investigated in this context include the Ritvo Autism & Asperger Diagnostic Scale (RAADS-14) [48], a brief version of the Ritvo Autism and Asperger Diagnostic Scale-Revised (RAADS-R) [49] designed to assess autistic traits in line with DSM-IV diagnostic criteria and recommended by NICE for autism assessment. The Broad Autism Phenotype Questionnaire (BAPQ) [50], which was also developed at the time of DSM-IV, is increasingly used in research to assess the broader continuum of autistic traits in the general population (>900 citations on Google Scholar). Findings for the BAPQ have been mixed [43, 51, 52], whereas the RAADS showed some item-level bias, though it was reported to be minimal and not systematically affecting total scores [53]. As with the AQ-50, caution is required in drawing conclusions about these measures at this stage, as further measurement invariance investigations are needed alongside research clarifying the practical implications of any identified bias for screening accuracy, particularly in light of ongoing debates in the literature regarding the relevance of measurement invariance [54–58].

Meanwhile, an increasing number of new self-report measures have also emerged in the literature [59–68], reflecting more contemporary understandings of autism and aligning with the updated DSM-5 criteria. Among these, the Comprehensive Autistic Trait Inventory (CATI) [63] has attracted particular interest in the literature. Findings from measurement invariance investigations conducted in English-speaking contexts consistently indicated that the CATI assesses autistic traits equivalently across sex/genders [43, 63, 69], suggesting it to be a promising measure for evaluating autistic traits without sex/gender bias. In response to concerns about sex/gender-related bias in existing instruments, some of these newly developed measures have been specifically designed to assess autistic traits in women, such as the Girls Questionnaire for Autism Spectrum Condition (GQ-ASC) [66], the Autistic Women’s Experience (AWE) [68], and FEM-A (only acronym provided in the original study) [67]. To date, investigations of sex/gender-related measurement invariance have not yet been conducted for these instruments. However, an important consideration is whether female-specific measures should, by design, be expected to demonstrate bias against males and/or gender-diverse individuals, and whether such instruments ultimately reduce or introduce further bias.

While measurement invariance is an important tool for detecting potential bias and evaluating validity, it should not necessarily be relied upon as the sole indicator of bias nor treated as the final stage of research inquiry [70]. Investigations of sex/gender-related bias of autism measures may instead require a more holistic approach, as experts emphasise that invariance testing may be best approached as a theory-guided process that statistical methods alone cannot replace [70]. Accordingly, scholars have suggested that measurement invariance investigations should be complemented by approaches that place greater emphasis on the underlying theoretical constructs being measured [70]. One way to support the development of theoretically driven autism measures, particularly to ensure their validity in capturing more nuanced presentations of autistic traits, could be achieved by evaluations of their content validity, which form part of the broader process of construct validation. Content validity can be understood as the extent to *which elements of an assessment instrument are relevant to and representative of the targeted construct for a particular assessment purpose* [71], based on qualitative or quantitative judgements from target population and subject-matter experts. Importantly, it applies not only to individual items but also to the measure as a whole, encompassing whether all essential aspects of the construct are represented, irrelevant items are excluded, and no single facet of the construct disproportionately influences the overall score [71]. Although content validity is often regarded as the most important step in scale development [72], it can be overlooked or implicitly assumed in the development process [72, 73]. Its significance is underscored by methodological guidelines advising that measures lacking sufficient content validity should neither be further evaluated nor recommended for use [72].

Only recently have a small number of qualitative and mixed-methods studies begun to investigate the content validity of self-report measures of autistic traits for autistic women, with findings often highlighting their limitations [74–76]. In English-speaking contexts, widely used measures such as the AQ-10, AQ-50, BAPQ, and RAADS-14 were perceived by autistic women and individuals socialised as female as reinforcing stereotypical portrayal of autism and as unsuitable for adult women [74, 76]. They also noted missing items on domains such as masking, empathy, internal experiences, daily life skills, and relationship challenges, which were areas considered particularly relevant to their experiences [76]. Items were further perceived as ambiguous and lacking sufficient context or nuance [74, 76], concerns that may likewise apply to the autistic population more broadly. [76, 77] In one of these studies [74], autistic women were also asked about the relevance of GQ-ASC, a measure specifically designed to assess autistic traits in females. Although participants regarded it as less appropriate for adults and late-diagnosed women, and found comparisons with other girls especially difficult, they still viewed it more favourably than the AQ-50 [74]. Notably, however, these qualitative findings contrasted with the same study’s closed-ended responses, in which autistic women rated both the AQ-50 and GQ-ASC as similarly relevant.

In this study, we aim to complement existing sex/gender-related measurement invariance investigations as well as the qualitative work conducted to date, by employing the modified Delphi methodology - an iterative, multi-round approach for achieving expert consensus in under-researched areas [78], to assess the content validity of commonly used self-report measures of autistic traits for autistic women (see Methods for the rationale for measure selection). This approach extends prior statistical work within the sex/gender-related measurement invariance framework by providing a substantive evaluation of whether conclusions based on statistical indices are supported at the conceptual level. Because issues of measurement invariance ultimately reflect limitations in the underlying theoretical conceptualisation of the construct [70], the present study examines whether the conceptualisation of autism embedded in these measures aligns with autistic women’s experiences, thereby facilitating the identification of potential sources of gender-related bias. Further, measurement invariance analyses are limited to detecting bias within an existing item set and cannot address bias arising from construct underrepresentation, which was explicitly examined in the present work. The study further extends prior qualitative work by evaluating content validity at the item level, enabling more fine-grained analysis than previous scale-level investigations [74–76], and by integrating evaluations from both autistic women (experts by experience) and professional experts, the latter not previously incorporated in content validity assessments of autistic trait measures for autistic women.

Content validity was appraised using both quantitative and qualitative survey-based assessments of item relevance and comprehensiveness of measures for capturing autism in women. Quantitative feedback from autistic women, drawing on their lived experience, together with input from academics and clinicians with extensive expertise in autism in women, was used to build consensus on the most relevant items, thereby generating a preliminary pool for further testing. Although the primary focus of this study was on item-level relevance, the measures were also evaluated as a whole, based on these item-level ratings, to determine whether any were considered appropriate for autistic women in their current form. In line with previous research on content validity [79], we report only the quantitative results from Round 1, both because of their scope and to emphasise the importance of thoroughly documenting each stage of measurement development, particularly content validity, as theoretical assessments of constructs are often insufficiently addressed in research.

## Methods

### Study design

Ethics approval for the study was obtained from King’s College London Psychiatry, Nursing & Midwifery Research Ethics Subcommittee (Reference Number: LRS/DP-22/23–34273).

A mixed method modified Delphi study was employed to evaluate the content validity of items from existing self-report questionnaires for autism in relation to their relevance for assessing autism in autistic women. The Delphi method proves valuable in obtaining consensus among experts on under-researched topics, which is accomplished through an iterative approach that builds upon the results of each preceding round [78]. The traditional Delphi, typically begins with open-ended questions followed by the development of a questionnaire based on participants’ responses. This study employed a modified Delphi design [78], whereby participants, comprising experts either with professional (academics/clinicians) or lived (autistic women) experience, were presented with a predefined list of statements to rate. In Delphi studies, two to three rounds are typically required to gather essential information and to achieve a consensus [78, 80]. To mitigate the risk of participant fatigue, it is advisable to limit the number of iterations [81]. In adherence to these guidelines, we have opted for two rounds in this study, with the quantitative results from the first presented here.

In Round 1, participants evaluated individual items from selected measures of autistic traits in terms of their relevance for autistic women, both quantitatively (by rating relevance) and qualitatively (by providing optional explanations and suggesting alternative wording). Item-level ratings were then used to assess the relevance of each measure as a whole. Additionally, experts assessed the suitability of a measure’s response scale and identified whether any relevant experiences were not captured by existing measures, both quantitatively and through optional qualitative feedback. This process resulted in a preliminary item pool of the most relevant items for autistic women, which will be refined based on qualitative feedback. The revised items will be evaluated in Round 2, with the ultimate goal of subsequent psychometric testing.

### Selection and recruitment of experts and final sample characteristics

Recommendations for panel size in Delphi studies vary, with the number of participants contingent upon the scope of the problem and available resources [82–84]. Following suggestions for 15–30 participants or 5–10 per category from different professional groups [85], our panel target was 10 academics/researchers and clinicians (AC), and 10 autistic women (AW) per block of questionnaires in Round 1, totalling 60 participants for three blocks.

Expert panel selection criteria draw from prior Delphi studies on autism diagnosis in women [86] and the female autism phenotype [87]. Academics and researchers were eligible to participate if a) they had been awarded a PhD degree, b) had a minimum of five years of autism research experience inclusive of their PhD training, c) had at least one publication on autistic women or gender differences in autism, and d) English fluency. Clinicians were required to have a) 5 years of experience in the diagnosis of autistic adults, b) professional experience with a minimum of 100 cases involving autistic women, and c) fluency in English. Academics and clinicians who had co-authored any of the self-report questionnaires evaluated in this study (see Measures section) were not eligible to participate. Inclusion criteria for autistic women required that participants either had a) a formal autism diagnosis or self-identified as autistic, b) identified as (cis/trans) woman, c) were 18 years of age or older, d) were fluent in English, and e) did not have a diagnosis of severe intellectual or learning disability (excluding conditions such as dyslexia, dyspraxia, dyscalculia).

Participants were recruited through different channels based on their respective expert groups. Academics were identified via scientific databases and invited to participate through direct outreach. Clinicians were recruited through the England’s Autism Practitioner Network, the King’s College London Research Volunteer Recruitment Circular, and direct outreach. Autistic women were recruited via the Autistica, the online research platform Prolific, the King’s College London Research Volunteer Recruitment Circular, and social media platforms. To ensure the inclusion of underrepresented communities within autistic women group [88, 89], participants from ethnic minority backgrounds were prioritised for invitations to the Delphi survey. Additional recruitment efforts specifically targeting individuals from Black and Asian backgrounds were conducted via the Prolific platform, in response to initially low participation from these groups.

A total of 76 participants started the Delphi survey; however, 10 were excluded due to fully missing or duplicate data (see Section ‘Pre-screening’ for details), resulting in a final sample of 66 participants, comprising two panels: 33 autistic women and 33 academics/clinicians. Detailed demographic characteristics are presented in Table 1.

**Table 1:** Demographic characteristics of the sample (*N* = 66)

Characteristic		Frequency	
		AW (N = 33)	AC (N = 33)
**Gender**	Woman (%)	^a^ 33 (100.0)	^b^ 30 (90.9)
	Man (%)	0 (0.0)	^b^ 2 (6.1)
	Agender (%)	0 (0.0)	1 (3.0)
**Age (years)**	Mean (SD)	32.8 (9.1)	^c^ 43.5 (9.3)
	Median (IQR)	30 (12.0)	^c^ 44 (11.5)
	Range	20 - 57	^c^ 26 - 63
**Ethnicity**	Asian (%)	3 (9.1)	1 (3.0)
	Black (%)	8 (24.2)	0 (0.0)
	Mixed or Multiple Ethnicities (%)	1 (3.0)	4 (12.1)
	White (%)	21 (63.6)	28 (84.8)
**Country**	Australia (%)	0 (0.0)	5 (15.2)
	Austria (%)	1 (3.0)	0 (0.0)
	Belgium (%)	1 (3.0)	0 (0.0)
	Canada (%)	1 (3.0)	0 (0.0)
	Malaysia (%)	1 (3.0)	0 (0.0)
	Netherlands (%)	1 (3.0)	0 (0.0)
	South Africa (%)	7 (21.2)	0 (0.0)
	United Kingdom (%)	19 (57.6)	23 (69.7)
	United States of America (%)	2 (6.1)	5 (15.2)
**Education**	Secondary/High school graduate (%)	2 (6.1)	-
	Vocational or Professional Qualifications (%)	3 (9.1)	-
	Foundation or Access-level Certificate or Diploma (%)	2 (6.1)	-
	Bachelor’s degree (%)	13 (39.4)	-
	Master’s degree (%)	8 (24.2)	-
	Doctoral-level degree (%)	5 (15.2)	-
**Autism**	Diagnosed (%)	22 (66.7)	4 (12.1)
Age of diagnosis (years)	Mean (SD)	^d^ 25.5 (13.0)	36.8 (4.9)
	Median (IQR)	^d^ 27.0 (18.0)	37 (8.2)
	Range	^d^ 3- 54	32 - 41
	Self-identify (%)	11 (33.3)	0 (0.0)
Age of self-identification (years)	Mean (SD)	27.9 (11.2)	-
	Median (IQR)	27 (10.5)	-
	Range	15 - 55	-
	No diagnosis/self-identification (%)	0 (0.0)	27 (81.8)
	Not sure (%)	0 (0.0)	1 (3.0)
	Prefer not to say (%)	0 (0.0)	1 (3.0)
**Neurodivergence**	Attention deficit hyperactivity disorder (%)	14 (42.4)	8 (24.2)
	Dyslexia (%)	6 (18.2)	3 (9.1)
	Dyscalculia (%)	4 (12.1)	0 (0.0)
	Dyspraxia (%)	7 (21.2)	0 (0.0)
	Other neurodivergence (%)	1 (3.0)	3 (9.1)
	Not sure (%)	2 (6.1)	2 (6.1)
	No other neurodivergence (%)	13 (39.4)	18 (54.5)
	Prefer not to say (%)	0 (0.0)	1 (3.0)

*Note.* AW: autistic women; AC: academics and clinicians; SD: standard deviation; IQR: interquartile range. Due to a rounding error, some columns may total 99.9% or 100.1% instead of 100%. ^a^ All participants identified as cis women except one, who preferred not to disclose their cis or trans identity. ^b^ All participants identified as cis gender. ^c^*N* = 32, as one participant preferred not to disclose their age. ^d^*N* = 21, as one participant reported an age of diagnosis of 1, which was considered implausible and treated as missing data

With respect to specialised professional expertise, the academic/clinician panel comprised 18 academic experts (54.55%), 8 (44.4%) of whom also practiced clinically, and 15 clinicians (45.45%), 5 (33.3%) of whom also held academic or research roles. Among academic experts, 15 (83.3%) had a background in psychology, with Lecturer, Assistant Professor, or equivalent senior researcher roles being the most commonly held positions (*n* = 6, 33.3%). On average, participants had 11.4 years of research experience in the field of autism (SD = 6.4) and had published 4.2 articles (SD = 3.4) on autism in women or gender differences in autism. Among clinical experts, the most frequently reported role was Clinical Psychologist (*n* = 6, 40%), and the majority (*n* = 11, 73.3%) worked in autism assessment services. Clinicians reported an average of 10.8 years (SD = 4.2) of experience diagnosing autism in adults. The most commonly reported estimate for the number of autistic women assessed was 101–150 cases (*n* = 5, 33.3%) but ranged from 51 to 100 to >400. Detailed expertise characteristics are presented in Additional File Tables A1 and A2.

### Measures

The items evaluated in this study were drawn from peer-reviewed, self-report measures of autism, selected according to several criteria. First, measures were included if they had either been widely used in research as evidenced by over 150 citations on Google Scholar, or published after 2014, following the 2013 revision of the DSM [2] for autism, thereby allowing a one-year buffer for the publication of measures aligned with the updated diagnostic criteria. For more recent measures, a threshold of at least 50 Google Scholar citations was used, reflecting emerging recognition in the literature while also keeping the number of measures administered manageable. Second, measures had to be validated for use with adults and available as open-access tools to prioritise the evaluation of the most accessible measures. Therefore, measures requiring a licence, such as the Social Responsiveness Scale-2 [90], were not included. Instruments were excluded if they assessed only one of the two core domains of autism. When eligible measures contained more than 50 items and a validated abridged version was available, the shorter version was selected to reduce participant burden. For example, the RAADS-14 [48] was used instead of the original 80-item version [49]. Conversely, when multiple shortened forms of a scale existed, the original version was retained, provided it did not exceed 50 items, as it encompassed the full range of content found in the shorter forms. This was the case for the AQ-50 [40], which has several shorter forms in the literature [45, 91–95], and consequently, the full 50-item version was used in this study. The measures that met these criteria and were included in the study are described below.

The **AQ-50** [40] is a 50-item self-report measure designed to assess the degree of autistic traits in adults without a co-occurring intellectual disability. In the original validation study, the autistic subsample was predominantly male, with a male-to-female ratio of 3.5:1. The measure uses a four-point response scale ranging from ‘definitely agree’ to ‘definitely disagree’; however, for scoring purposes, responses are dichotomised, with all levels of agreement and disagreement grouped together. Items were originally organised into five trait dimensions, each comprising 10 items: *social skill* (Cronbach’s alpha, α = 0.77), *attention switching* (α = 0.67), *attention to detail* (α = 0.63), *communication* (α = 0.65), and *imagination* (α = 0.65). The AQ-50 is widely used in autism research, as evidenced by over 8,000 citations on Google Scholar at the time of measures’ selection (October-November 2023).

The **BAPQ** [50] is a 36-item self-report questionnaire originally developed to assess ‘milder’ or ‘broader autism phenotype’ traits commonly observed in close relatives of autistic individuals. However, it is now frequently used to measure the continuum of autistic traits in the general population. In line with its intended application, validation was conducted in parent samples, with parents of autistic children comprising a predominantly female subsample (male-to-female ratio 0.75:1). Responses are recorded on a six-point response scale ranging from ‘very rarely’ to ‘very often.’ The items are evenly divided across three trait dimensions: *pragmatic language deficits* (α = 0.85), *rigid personality* (α = 0.91) and *aloof personality* (α = 0.94). The BAPQ is well-established in autism research, with over 860 citations on Google Scholar.

The **CATI** [63] is a recently developed 42-item measure that relies on self-report to assess autistic traits in the general population, which was designed in response to advances in the understandings of autism. Validation was conducted across multiple studies, with autistic subsamples reported as relatively balanced with respect to sex (male-to-female ratio = 0.75–1.17:1). Each item is rated on a five-point response scale, ranging from ‘definitely disagree’ to ‘definitely agree.’ The measure covers six dimensions, with each comprising seven items: *social interaction* (α = 0.93–0.95), *communication* (α = 0.83), *sensory sensitivity* (α = 0.80–0.83), *repetitive behaviour* (α = 0.84–0.86), *cognitive rigidity* (α = 0.81–0.85), and *social camouflage* (α = 0.84–0.87). Since its publication, the CATI has been cited over 100 times on Google Scholar.

The **GQ-ASC** [66] is a 21-item autism screening tool designed to identify autism in adult women. It was developed to capture more nuanced presentations of autistic traits often observed in women. Originally created as a parent-report measure for younger girls and a self-report tool for older girls and teenagers, it has since been adapted for use with adults. In its validation, the autistic subsample was almost exclusively female (male-to-female ratio = 0.01:1). The measure uses a four-point response scale ranging from ‘definitely disagree’ to ‘definitely agree.’ Items are organised into five dimensions: *camouflaging* (McDonald’s omega, ω=0.67), *sensory sensitivities* (ω=0.58), *socialising* (ω=0.58), *imagination and play* (ω=0.64) and *interests* (ω=0.56). As a relatively recent instrument, the GQ-ASC has received over 65 citations on Google Scholar since its publication.

The **RAADS-14** [48] is a self-report screening tool for autism that was validated across multiple phases, with autistic subsamples that were relatively balanced with respect to sex (male-to-female ratio = 0.74–1.41:1). Each item is rated on a four-point response scale that captures the presence of traits across the lifespan: ‘true now and when I was young,’ ‘true only now,’ ‘true only when I was younger than 16,’ and ‘never true.’ The items are grouped into three dimensions: *sensory reactivity* (α = 0.58–0.64), *mentalizing deficits* (α = 0.80–0.86), and *social anxiety* (α = 0.73–0.76). The RAADS-14 is well established in the literature, with over 200 citations on Google Scholar at the time of measures’ selection.

### The Delphi survey

The development of an online Delphi survey was informed by a prior qualitative study with autistic women and gender diverse individuals about relevance of existing self-report autism measures to their lived experiences [76]. The survey was first piloted with 28 autistic women, using slightly different versions across participants. Findings revealed inconsistencies in the interpretation of reverse-scored items (for instance, ‘I prefer to do things with others, rather than on my own’), with some participants interpreting these as indicative of autistic traits rather than their opposites, as evidenced by discrepancies between their quantitative responses and qualitative explanations. Accordingly, in the final version administered to autistic women, reverse-coded items were reworded to directly reflect autistic traits (for instance, changing ‘I prefer to do things with others, rather than on my own’ to ‘I prefer to do things on my own, rather than with others’; see Table A3). No such discrepancies were observed in the academic/clinician version, and items were retained in their original form. Autistic women who participated in the pilot, as well as all participants who completed the survey in full, received a £25 compensation in the form of a voucher.

The study comprised two consecutive Qualtrics surveys. The first served as a screening stage to mitigate fraudulent and bot responses, informed by prior research team experience [76] and existing literature [96, 97]. The study was advertised via a link to the screening survey, which incorporated reCAPTCHA and Qualtrics-based fraud detection. Respondents exceeding predefined thresholds for fraudulent (≥60) or duplicate scores (≥75) were automatically excluded. Eligible respondents completed screening questions, provided demographic and contact information, and generated a unique participant identifier (ID) based on prespecified items (e.g., year of birth, the second letter of the city of birth). This ID was used to link screening data to responses in the main survey for those invited to participate and served as a validity check, as key elements (e.g., year of birth) could be cross-verified against reported age. Only participants meeting inclusion and authenticity criteria were invited to complete the main Delphi survey. Additional validation checks were conducted after main survey completion by evaluating optional qualitative responses, which further supported the authenticity of the data.

**Fig. 1:**
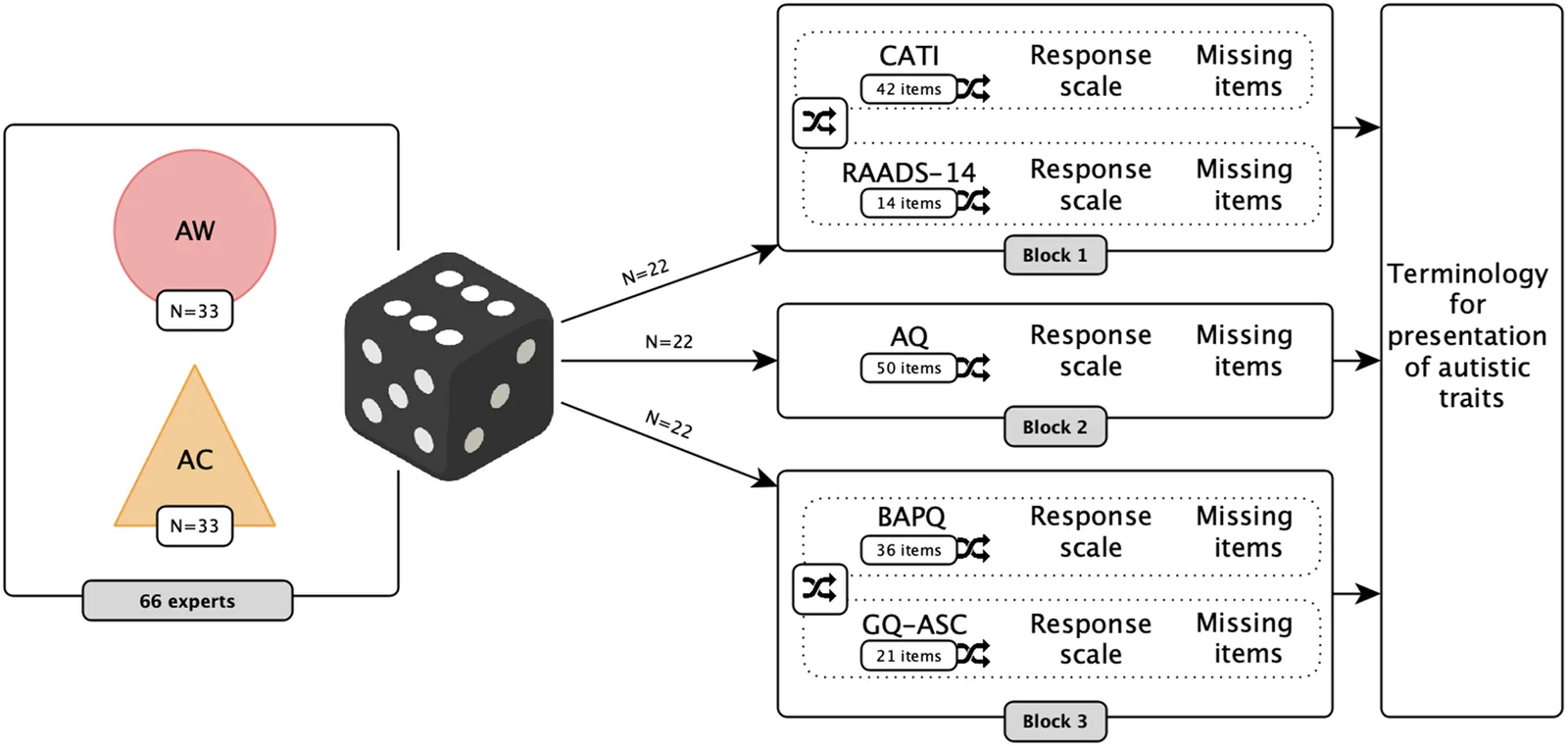
Overview of the Delphi survey process. *Note*. AW: autistic women, AC: academics/clinicians, AQ: the Autism Spectrum Quotient, BAPQ: the Broad Autism Phenotype Questionnaire, CATI: the Comprehensive Autistic Trait Inventory, GQ-ASC: the Girls Questionnaire for Autism Spectrum Condition, RAADS-14: the Ritvo Autism Asperger Diagnostic Scale 14

The first round of the Delphi survey was conducted between January and October 2024. Participants were provided with detailed study information and gave informed consent prior to participation. To address potential participant dropout [78], we have minimised survey length by randomly assigning experts to one of three questionnaire blocks (see Fig. 1), each containing one or two scales, corresponding to 50, 56 and 57 items. The order of questionnaires and their items within each block was randomised to prevent order effects [98].

Before beginning the survey, the participants received instructions on how to complete it, available in both written and video formats. To ensure comprehension of the instructions, they were required to correctly answer a practice question before proceeding. Due to the survey’s length, participants were informed they could take breaks, close the survey, and return to it later, with a two-week window to complete it. The median survey completion time was 59.98 minutes (range = 13.78– 12,057.52, IQR = 121.68) for academics/clinicians and 81.15 minutes (range = 13.55– 11,452.07, IQR = 323.07) for autistic women, with no statistically significant difference between groups (*U* = 437.00, *p* = 0.168, *r* = 0.17). Each of the two groups received a slightly different version of the survey tailored to their area of expertise. The main part of the survey included rating the relevance of individual statements to the experiences of autistic women using the following response options: (1) ‘Yes, it is relevant as it is’ (2), ‘Yes, it is relevant, but needs to be improved’ (3), ‘No, it is not relevant, but it can be modified to be relevant’ (4), ‘No, it is not relevant to autistic women’ (or ‘No, it is not relevant to me’ for autistic women’s survey), and (5) ‘No, it is not relevant to autism at all’. Participants could also provide optional qualitative feedback for each item.

The participants were then asked to evaluate the suitability of each questionnaire’s response scale and to indicate if any relevant experiences for autistic women were missing from the measure, using quantitative ratings and optional qualitative feedback. The final question pertained to identifying the most suitable terms for describing traits that are more commonly observed among autistic women, with responses to this question being evaluated in a separate study. At the end of the survey, experts were invited to indicate their interest in participating in a subsequent feedback round, to be conducted following the analysis of Round 1 data.

### Analytic strategy

#### Pre-screening

Of the 40 autistic women who began the survey, 7 were removed due to entirely missing data, leaving 33 in the final sample. Among those, 11 were assigned to the AQ. Ten participants (90.9%) completed the main survey, while one participant dropped out after completing 13.46% of the items. Similarly, 11 autistic women were assigned to BAPQ, of whom 10 (90.9%) completed the main survey, and one participant discontinued the survey after completing 42.11% of the items. All 9 participants assigned to the CATI completed the main survey in full, as did all 10 participants assigned to the GQ-ASC. Among the 11 autistic women assigned to RAADS-14, 10 (90.9%) submitted complete responses, while one participant dropped out after completing 31.25% of the items.

A total of 36 academics/clinicians started the survey; three participants were removed due to duplication or entirely missing data, resulting in a final sample of 33. Of these, 11 were randomly assigned to the AQ. Nine participants (81.8%) completed all items, while the remaining two discontinued the main part of the survey partway, completing 32.69% to 53.85% of the items. Eleven academics/clinicians were assigned to the BAPQ, of which eight (72.7%) had complete data, while three participants dropped out, having completed between 47.37% and 94.74% of the main survey. For the CATI, 10 (90.9%) out of 11 assignees completed it fully, with one dropout who completed 11.36% of items. All 10 participants assigned to the GQ-ASC, as well as all 10 assigned to the RAADS-14, completed their respective main part of surveys in full.

Due to the small sample size, the item-level focus of the analysis, and the derivation of scale-level content validity index from item-level results, participants who did not complete the survey in full were retained in the analysis.

#### Data analysis

The Delphi survey results were analysed separately per group. Agreement on an item being relevant to autistic women was evaluated using two coefficients: the item-level content validity index (I-CVI) and the modified kappa statistic (k^*^) [99],^2^ which adjusts I-CVI for chance agreement^3^. The I-CVI was calculated as the proportion of experts who rated an item as relevant, which is defined as a rating of either 1 (‘Yes, it is relevant as is’) or 2 (‘Yes, it is relevant but needs to be improved’). The same criteria were applied to responses evaluating whether a particular questionnaire captured the full range of autistic experiences relevant to autistic women, where relevance corresponded to a rating of 2 (‘No, I think everything has been covered), and to the evaluation of response scales, where relevance corresponded to ratings of 1 (‘Yes’) or 2 (‘Somewhat, it can be improved’). Agreement on non-relevance was excluded because, in content validity assessment, the extent to which participants achieved any type of agreement is not informative of item’s relevance [99]. The probability of chance agreement was then computed and used alongside the I-CVI to derive k^*^. Detailed frequencies and content validity indices for each item are available in Tables A4 to A13 in Additional File 1. The modified kappa values were interpreted based on established guidelines [100, 101], whereby values below 0.40 indicate poor agreement, 0.40–0.59 suggest fair agreement, 0.60–0.74 indicate good agreement, and above 0.74 represent excellent agreement. Any I-CVI ≥ 0.78 corresponds to excellent agreement for k^*^ [99]. Throughout this manuscript, if an item is described as ‘relevant,’ it has achieved excellent agreement (k * ≥0.74, I-CVI ≥ 0.78).

Although the primary focus was on item-level relevance, scale-level content validity (S-CVI/Ave) was also evaluated per expert group to determine whether any measure was considered relevant for autistic women in its current form. The S-CVI/Ave was obtained by averaging the I-CVI values of all items within a given measure. Following Polit et al [99]. and Waltz et al, [102] we set a target of 0.90 for the S-CVI/Ave, ensuring the measure includes balanced number of items that attained full agreement (I-CVI = 1.00) as well as some items with only modest disagreement (I-CVI ≥ 0.78).

In this study, an item was considered relevant for measuring autism in autistic women, and thus included in the preliminary pool for further evaluations, only if it achieved excellent agreement (k* ≥0.74, I-CVI ≥ 0.78) across *all* expert groups. This threshold served as the consensus criterion for this Delphi round. For instance, if academics/clinicians reached excellent agreement, but autistic women had only good or lower agreement, the item was not considered sufficiently relevant. This approach ensured that content of items is valid across clinical/academic, and lived-experience perspectives, and to provide equal weight to each group’s judgment.

Data analysis and visualisation were conducted in R Statistics version 4.3.1 for MacOS, with the pyramid plot code adapted from Keyes (2024) [103].

## Results

### Measures

Table 2 summarises the number of items within each measure that achieved excellent or lower agreement regarding their relevance for autistic women, including items with excellent agreement shared across both panels and those identified as relevant exclusively by one group. Detailed results for each measure are presented in the corresponding subsections.

**Table 2:** Frequency summary of items for each measure with excellent and lower agreement on relevance across and within two groups

Scale		Number of items within (sub)scale	Items with excellent agreement on being relevant			Items with below excellent agreement on being relevant across both groups (%)
			Across both AW and AC (%)	AW only (%)	AC only (%)	
AQ	*Total Scale*	50	17 (34.0)	3 (6.0)	10 (20.0)	20 (40.0)
	Attention switching	10	8 (80.0)	1 (10.0)	1 (10.0)	0 (0.0)
	Communication	10	6 (60.0)	0 (0.0)	3 (30.0)	1 (10.0)
	Social skills	10	2 (20.0)	1 (10.0)	2 (20.0)	5 (50.0)
	Attention to detail	10	1 (10.0)	1 (10.0)	2 (20.0)	6 (60.0)
	Imagination	10	0 (0.0)	0 (0.0)	2 (20.0)	8 (80.0)
BAPQ	*Total Scale*	36	10 (27.8)	13 (36.1)	4 (11.1)	9 (25.0)
	Pragmatic Language	12	4 (33.3)	2 (16.7)	3 (25.0)	3 (25.0)
	Rigid	12	4 (33.3)	7 (58.3)	0 (0.0)	1 (8.3)
	Aloof	12	2 (16.7)	4 (33.3)	1 (8.3)	5 (41.7)
CATI	*Total Scale*	42	30 (71.4)	4 (9.5)	7 (16.7)	1 (2.4)
	Cognitive rigidity	7	7 (100.0)	0 (0.0)	0 (0.0)	0 (0.0)
	Sensory sensitivity	7	7 (100.0)	0 (0.0)	0 (0.0)	0 (0.0)
	Social camouflage	7	5 (71.4)	0 (0.0)	2 (28.6)	0 (0.0)
	Social interactions	7	5 (71.4)	2 (28.6)	0 (0.0)	0 (0.0)
	Communication	7	3 (42.9)	0 (0.0)	4 (57.1)	0 (0.0)
	Repetitive behaviours	7	3 (42.9)	2 (28.6)	1 (14.3)	1 (14.3)
GQ-ASC	*Total Scale*	21	8 (38.1)	1 (4.8)	2 (9.5)	10 (47.6)
	Sensory sensitivities	4	3 (75.0)	0 (0.0)	1 (25.0)	0 (0.0)
	Socializing	4	3 (75.0)	1 (25.0)	0 (0.0)	0 (0.0)
	Camouflaging	4	2 (50.0)	0 (0.0)	1 (25.0)	1 (25.0)
	Imagination and play	5	0 (0.0)	0 (0.0)	0 (0.0)	5 (100.0)
	Interests	4	0 (0.0)	0 (0.0)	0 (0.0)	4 (100.0)
RAADS-14	*Total Scale*	14	12 (85.7)	1 (7.1)	1 (7.1)	0 (0.0)
	Sensory reactivity	3	3 (100.0)	0 (0.0)	0 (0.0)	0 (0.0)
	Mentalizing deficits	7	6 (85.7)	0 (0.0)	1 (14.3)	0 (0.0)
	Social anxiety	4	3 (75.0)	1 (25.0)	0 (0.0)	0 (0.0)

*Note.* AW: Autistic women, AC: Academics and clinicians, AQ: the Autism Spectrum Quotient, BAPQ: the Broad Autism Phenotype Questionnaire, CATI: the Comprehensive Autistic Trait Inventory, GQ-ASC: the Girls Questionnaire for Autism Spectrum Condition, RAADS-14: the Ritvo Autism Asperger Diagnostic Scale 14

Across all measures, there was poor agreement regarding their comprehensiveness in capturing the full range of relevant experiences for autistic women, but excellent agreement on the suitability of their response scales for accurate response expression (see Tables A14 and A15 for details).

#### The Autism Spectrum Quotient

At the scale-level, academics/clinicians rated the measure as more relevant (S-CVI/Ave = 0.71) than autistic women (S-CVI/Ave = 0.66); however, neither rating met the threshold for excellent content validity.

##### Item relevance

**Fig. 2:**
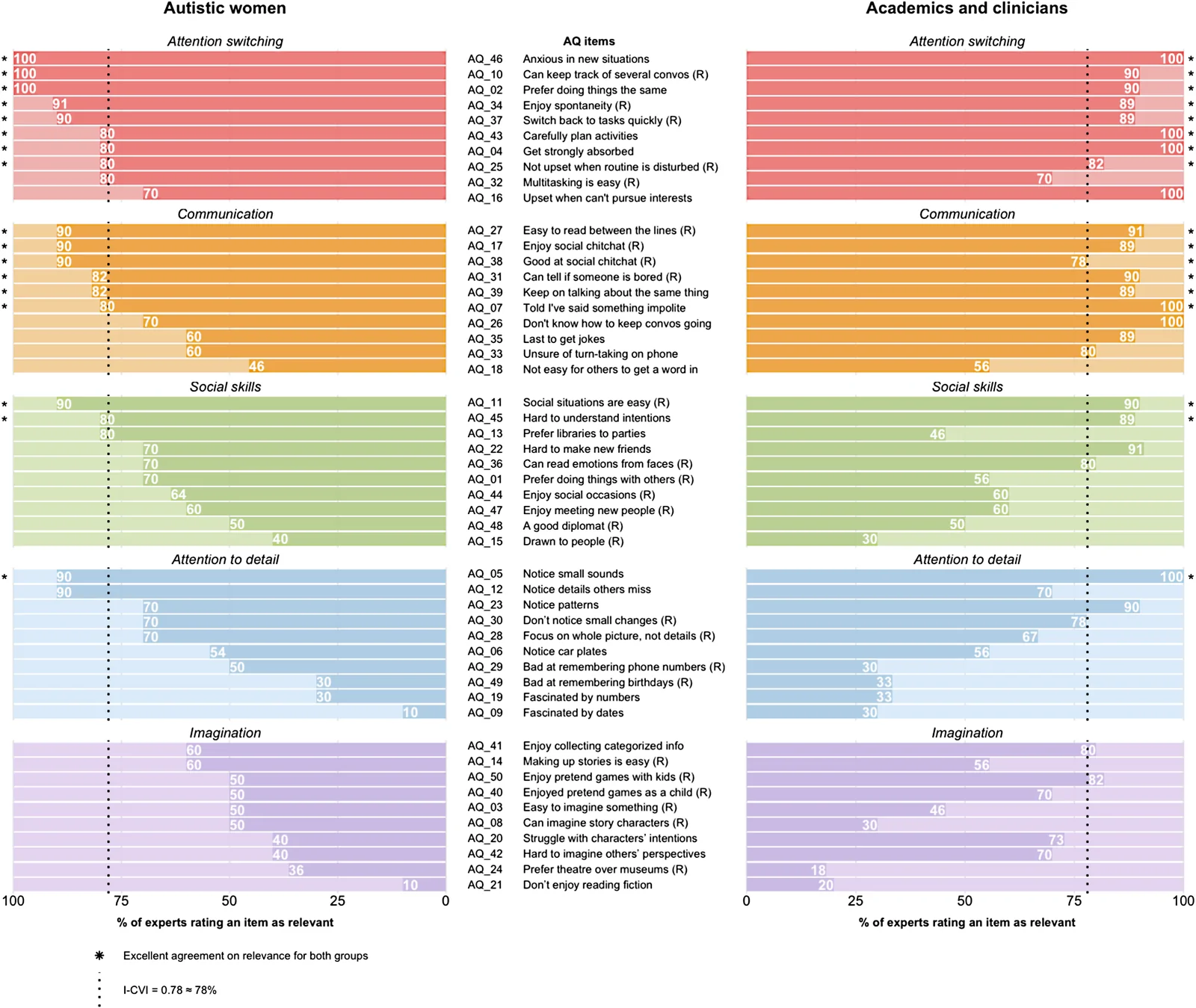
Percentage of autistic women (left) and academics/clinicians (right) rating each AQ item as being relevant (values correspond to I-CVIs × 100) to measuring autism in autistic women, per each factor. *Note.* Bars with * correspond to items that achieved excellent agreement on being relevant across both expert groups. Dotted vertical line represents I-CVI * ≥ *0.78 ≈ 78%, which corresponds to excellent agreement for modified kappa (Polit et al., 2007). Relevant items are those rated by experts as 1 (‘yes, it is relevant as is’) or 2 (‘yes, it is relevant but needs to be improved’). Non-relevant items are those rated as 3 (‘no, it is not relevant but it can be modified to be relevant’), 4 (‘no, it is not relevant to autistic women/to me’), or 5 (‘no, it is not relevant to autism at all’). (**R**) signifies a reverse scored statement

Figure 2 illustrates the percentage of AQ items rated as relevant to measuring autism in autistic women, as evaluated separately by the two groups. Across the 50 AQ items, 17 (34%) were identified as having excellent agreement on being relevant by both groups (see Table 2). Only one item (2%; AQ46, *anxious in new situations*) received unanimous rating (100%) of being relevant across autistic women and academics/clinicians. A discrepancy in ratings was observed for 13 items (26%). Autistic women endorsed three additional items (6%; AQ12, AQ13, AQ32) that were not rated as relevant by academics/clinicians, whereas academics/clinicians identified 10 items (20%; AQ16, AQ22, AQ23, AQ26, AQ30, AQ33, AQ35, AQ36, AQ41, AQ50) as relevant that were not endorsed by autistic women.

The largest difference in percentage relevance ratings between the two groups (34%) was observed for AQ13 (*prefer libraries to parties*), rated relevant only by autistic women. Other items with pronounced differences (≥30%) were AQ50 (*enjoy pretend games with kids (reversed)*, 32%), AQ26 (*don’t know how to keep conversations going*, 30%), and AQ16 (*upset when can’t pursue interest*, 30%), all of which were endorsed by academics/clinicians but not autistic women. Please see Figure A1 in the Additional File 1 for percentage differences in relevance ratings for all AQ items.

##### Domain-specific relevance

The AQ items refer to five constructs, two aligned with the core diagnostic domain of social interaction and communication (*communication* and *social skills* factors) and one aligned with the domain of RRBIs (*attention switching* factor). The remaining two factors, *imagination* and *attention to detail*, reflected autism-related constructs outside the diagnostic criteria. Most RRBI-related items were rated as relevant (8/10). Fewer than half of the social communication items were deemed relevant (8/20), and only one of the 10 attention to detail items was rated as relevant. None of the imagination items was considered relevant.

#### The Broad Autism Phenotype Questionnaire

The overall scale did not meet the threshold for excellent content validity for assessing autism in autistic women. However, it was rated as more relevant by autistic women (S-CVI/Ave = 0.80) than by academics/clinicians (S-CVI/Ave = 0.74).

##### Item relevance

**Fig. 3:**
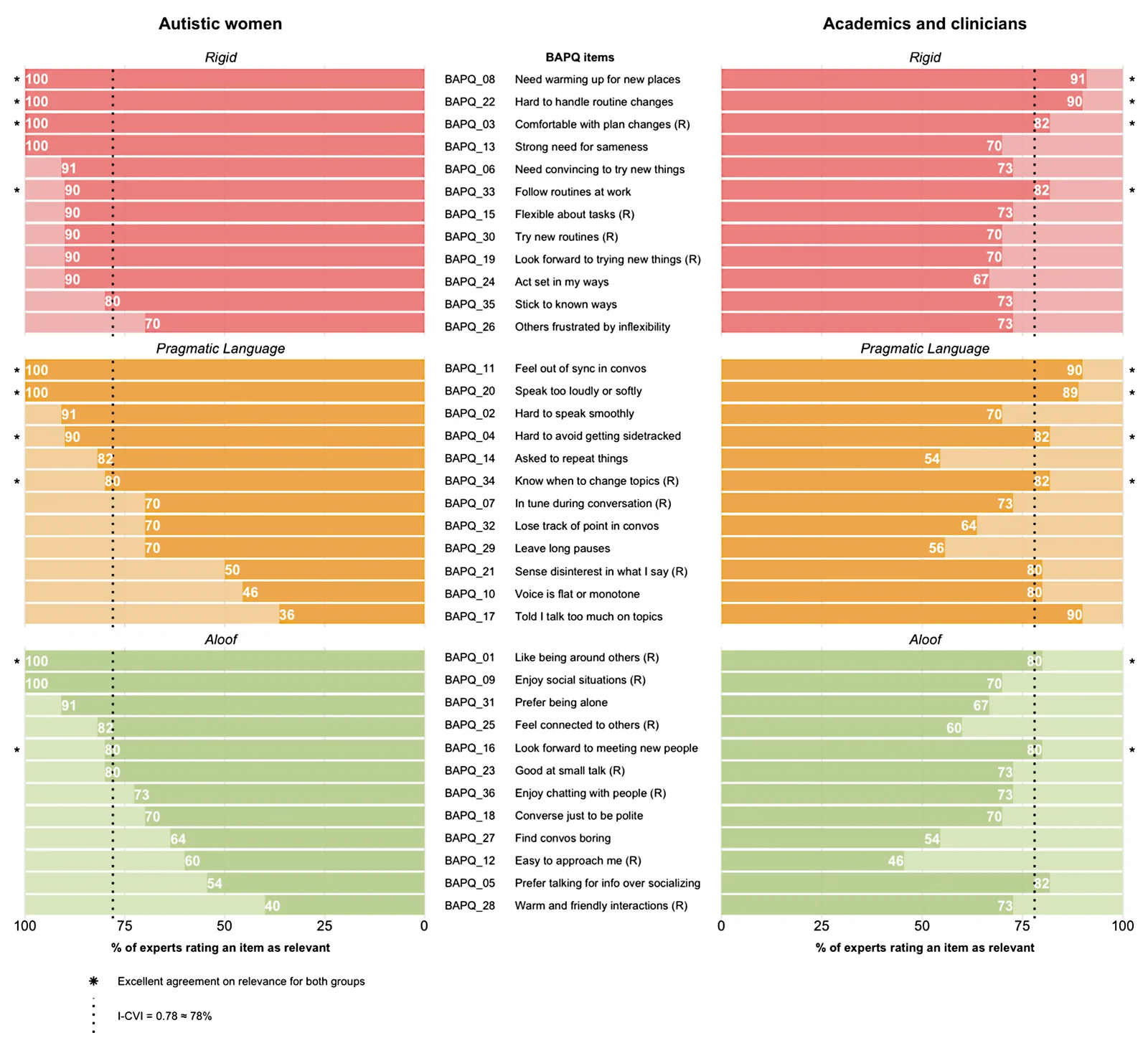
Percentage of autistic women (left) and academics/clinicians (right) rating each BAPQ item as being relevant (values correspond to I-CVIs × 100) to measuring autism in autistic women, per each factor. *Note.* Bars with * correspond to items that achieved excellent agreement on being relevant across both expert groups. Dotted vertical line represents I-CVI * ≥ *0.78 ≈ 78%, which corresponds to excellent agreement for modified kappa (Polit et al., 2007). Relevant items are those rated by experts as 1 (‘yes, it is relevant as is’) or 2 (‘yes, it is relevant but needs to be improved’). Non-relevant items are those rated as 3 (‘no, it is not relevant but it can be modified to be relevant’), 4 (‘no, it is not relevant to autistic women/to me’), or 5 (‘no, it is not relevant to autism at all’). (**R**) signifies a reverse scored statement

Excellent agreement on an item being relevant across both autistic women and academics/clinicians was achieved for 10 (27.8%) of the 36 items (see Fig. 3 and Table 2). No item achieved full consensus (100%) on being relevant across both groups. Autistic women identified 13 additional items (36.1%; BAPQ02, BAPQ06, BAPQ09, BAPQ13, BAPQ14, BAPQ15, BAPQ19, BAPQ23, BAPQ24, BAPQ25, BAPQ30, BAPQ31, BAPQ35) as relevant that were not endorsed by academics/clinicians. Conversely, academics/clinicians rated four items (11.1%; BAPQ05, BAPQ10, BAPQ17, BAPQ21) as relevant that were not endorsed by autistic women.

Among these items, the largest discrepancies in percentage relevance ratings between autistic women and academics/clinicians (≥30%) occurred for BAPQ17 (*told I talk too much on topics*, 53.6%), BAPQ10 (*voice is flat or monotone*, 34.5%), and BAPQ21 (*sense disinterest in what I say (reversed)*, 30%), all of which were rated as relevant only by academics/clinicians. Autistic women exclusively rated BAPQ09 (*enjoy social situations (reversed)*) and BAPQ13 (*strong need for sameness*) as relevant, with a 30% higher rating than those of academics/clinicians. Refer to Figure A2 in Additional File 1 for details for all BAPQ items.

##### Domain-specific relevance

The BAPQ items refer to three constructs, two of which are related to the social-communication domain of autism (*aloof* and *pragmatic language* subscales), and one targeted RRBIs (*rigid* factor). Less than half of the social communication items (6/24) and RRBIs items (4/12) were rated as relevant.

#### The Comprehensive Autistic Trait Inventory

Academics/clinicians rated the overall CATI scale as having excellent content validity for assessing autism in autistic women (S-CVI/Ave = 0.90). Ratings from autistic women, while relatively high, did not meet this threshold (S-CVI/Ave = 0.84).

##### Item relevance

**Fig. 4:**
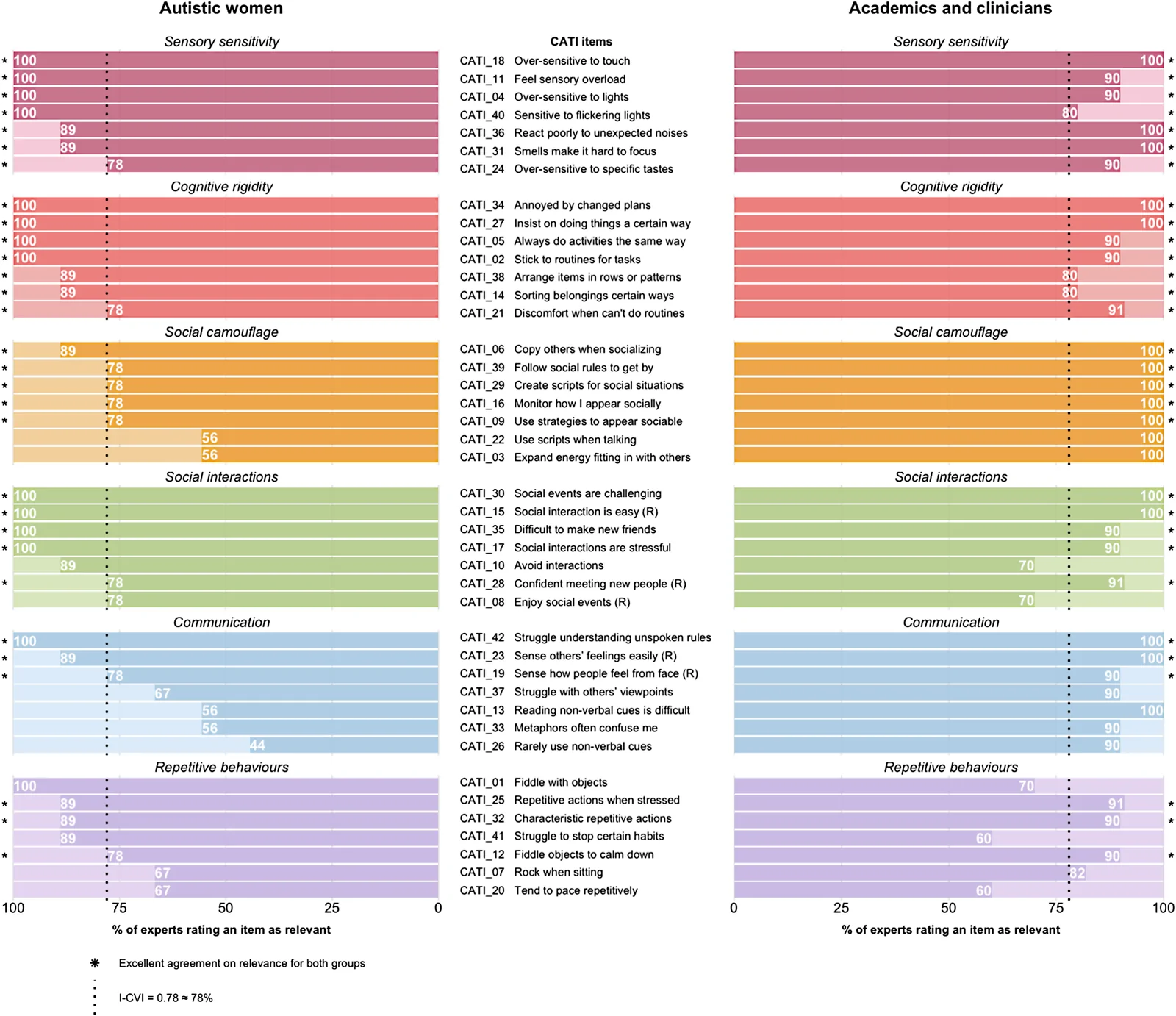
Percentage of autistic women (left) and academics/clinicians (right) rating each CATI item as being relevant (values correspond to I-CVIs × 100) to measuring autism in autistic women, per each factor. *Note.* Bars with * correspond to items that achieved excellent agreement on being relevant across both expert groups. Dotted vertical line represents I-CVI * ≥ *0.78 ≈ 78%, which corresponds to excellent agreement for modified kappa (Polit et al., 2007). Relevant items are those rated by experts as 1 (‘yes, it is relevant as is’) or 2 (‘yes, it is relevant but needs to be improved’). Non-relevant items are those rated as 3 (‘no, it is not relevant but it can be modified to be relevant’), 4 (‘no, it is not relevant to autistic women/to me’), or 5 (‘no, it is not relevant to autism at all’). (**R**) signifies a reverse scored statement

Of the 42 CATI items, 30 (71.4%) had excellent agreement on being relevant by both autistic women and academics/clinicians (see Fig. 4 and Table 2). Six items (14.28%), namely CATI42 (*struggle understanding unspoken rules*), CATI34 (*annoyed by changed plans*), CATI30 (*social events are challenging*), CATI27 (*insist on doing things a certain way*), CATI18 (*over-sensitive to touch*), and CATI15 (*social interaction is easy*), received unanimous endorsement (100%) from both groups. Discrepancies between the two groups arose for 11 items (26.2%). Autistic women identified four items (CATI01, CATI08, CATI10, CATI41) as relevant that were not endorsed by academics/clinicians, while academics/clinicians rated seven items (CATI03, CATI07, CATI13, CATI22, CATI26, CATI33, CATI37) as relevant that were not endorsed by autistic women.

Items with the greatest discrepancies (≥30%) in percentage relevance ratings between the two groups included CATI26 (*rarely use non-verbal cues*, 45.6%), CATI03 (*expand energy fitting in with others*, 44.4%), CATI13 (*reading non-verbal cues is difficult*, 44.4%), CATI22 (*use scripts when talking*, 44.4%), CATI33 (*metaphors often confuse me*, 34.4%), and CATI01 (*fiddle with objects*, 30%). All but the latter (CATI01), which was endorsed only by autistic women, were rated as relevant solely by academics/clinicians. For percentage differences in relevance ratings for all CATI items, see Figure A3 in Additional File 1.

##### Domain-specific relevance

Out of six factors captured by CATI, five corresponded to the core diagnostic domains of autism. Three were related to RRBIs (*sensory sensitivity*, *cognitive rigidity*, and *repetitive behaviours* factors), while two reflected the social communication domain (*social interactions* and *communication* factors). The one remaining factor represented autism-related construct of masking (*social camouflage* factor). Among the core diagnostic domains, the majority of RRBI items (17/21) and just over half of the social communication items (8/14) were considered relevant. The majority of items aligning with masking (5/7) were rated as relevant.

#### The Girls Questionnaire for Autism Spectrum Condition

Academics/clinicians rated the overall measure as more relevant (S-CVI/Ave = 0.71) than autistic women (S-CVI/Ave = 0.66); however, none meet the threshold for excellent content validity.

##### Item relevance

**Fig. 5:**
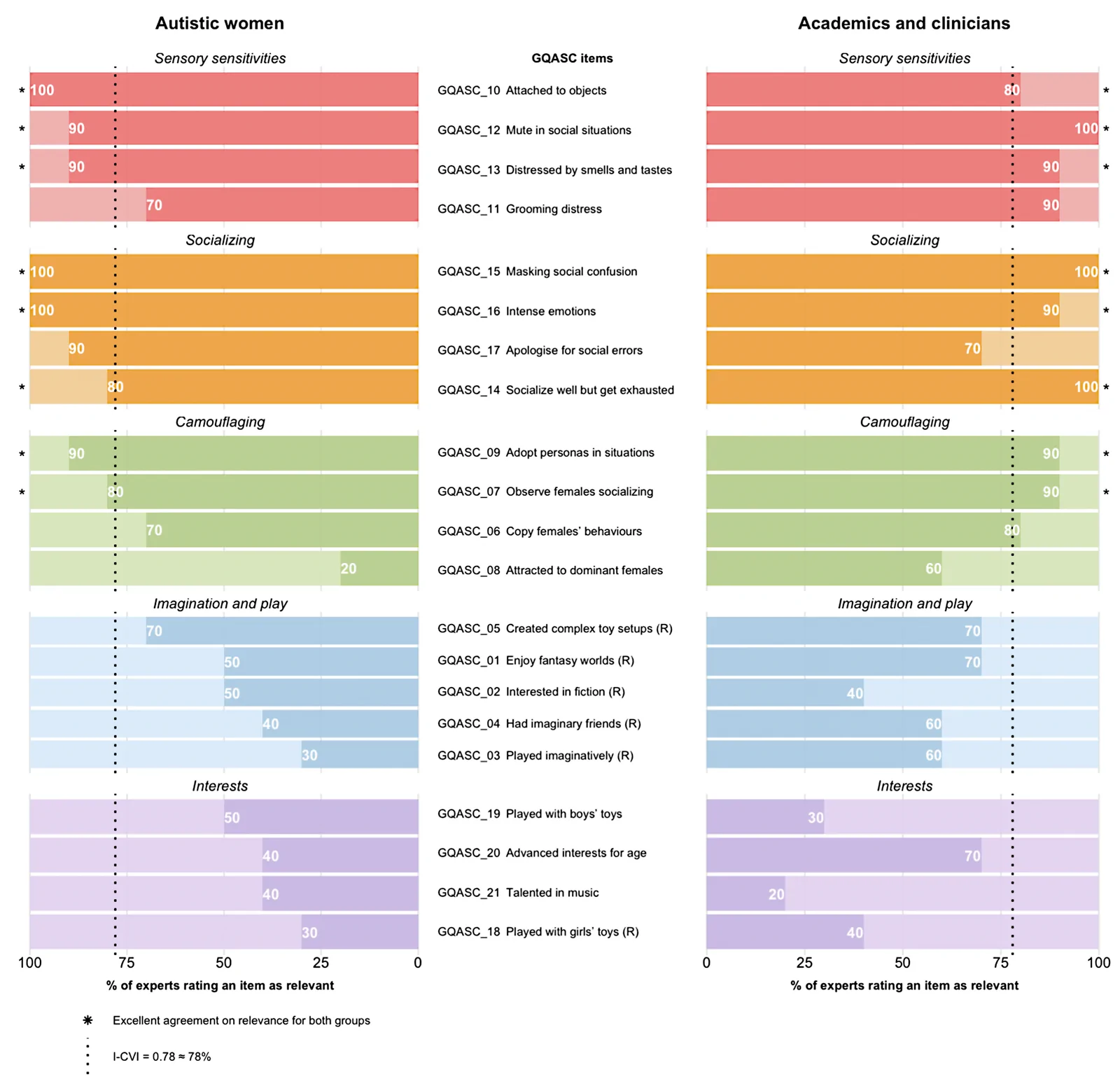
Percentage of autistic women (left) and academics/clinicians (right) rating each GQ-ASC item as being relevant (values correspond to I-CVIs × 100) to measuring autism in autistic women, per each factor. *Note.* Bars with * correspond to items that achieved excellent agreement on being relevant across both expert groups. Dotted vertical line represents I-CVI * ≥ *0.78 ≈ 78%, which corresponds to excellent agree ment for modified kappa (Polit et al., 2007). Relevant items are those rated by experts as 1 (‘yes, it is relevant as is’) or 2 (‘yes, it is relevant but needs to be improved’). Non-relevant items are those rated as 3 (‘no, it is not relevant but it can be modified to be relevant’), 4 (‘no, it is not relevant to autistic women/to me’), or 5 (‘no, it is not relevant to autism at all’). (**R**) signifies a reverse scored statement

Across the 21 GQ-ASC items, 8 (38.1%) achieved excellent agreement on being relevant across the two groups (see Fig. 5 and Table 2). Only one item (4.76%; GQASC15, *masking social confusion*) was rated fully (100%) relevant across autistic women and academics/clinicians. Discrepancies in relevance ratings were found for 3 items (14.3%). Autistic women had excellent agreement on one additional item (4.8%; GQASC17) that was not endorsed by academics/clinicians, while academics/clinicians rated two items as relevant (9.5%; GQASC06, GQASC11) that were not endorsed by autistic women.

The largest rating differences (20%) were observed for GQASC17 (*apologize for social errors*), endorsed only by autistic women, and GQASC11 (*grooming distress*), rated relevant solely by academics/clinicians. Please see Figure A4 in Additional File 1 for percentage differences in relevance ratings for all GQ-ASC items.

##### Domain-specific relevance

GQ-ASC items were organised into five dimensions, of which one targeted the core diagnostic domain of social communication (*socialising* factor), and two aligned with the domain of RRBIs (*sensory sensitivities* and *interests* factors). The remaining two dimensions corresponded to autism-related construct of masking (*camouflaging* factor) and imagination (*imagination and play* factor). Among the core diagnostic domains, the majority of social communication items (3/4) were rated as relevant, while fewer than half of the RRBI items (3/8) were deemed relevant. For the non-diagnostic constructs, none of the imagination items (0/5) and only half of the masking items (2/4) were rated as relevant.

#### The Ritvo Autism and Asperger Diagnostic Scale-14

At scale level, both autistic women (S-CVI/Ave = 0.92) and academics/clinicians (S-CVI/Ave = 0.96) rated RAADS-14 as having excellent content validity for assessing autism in autistic women, with academics/clinicians rating it more relevant than autistic women.

##### Item relevance

**Fig. 6:**
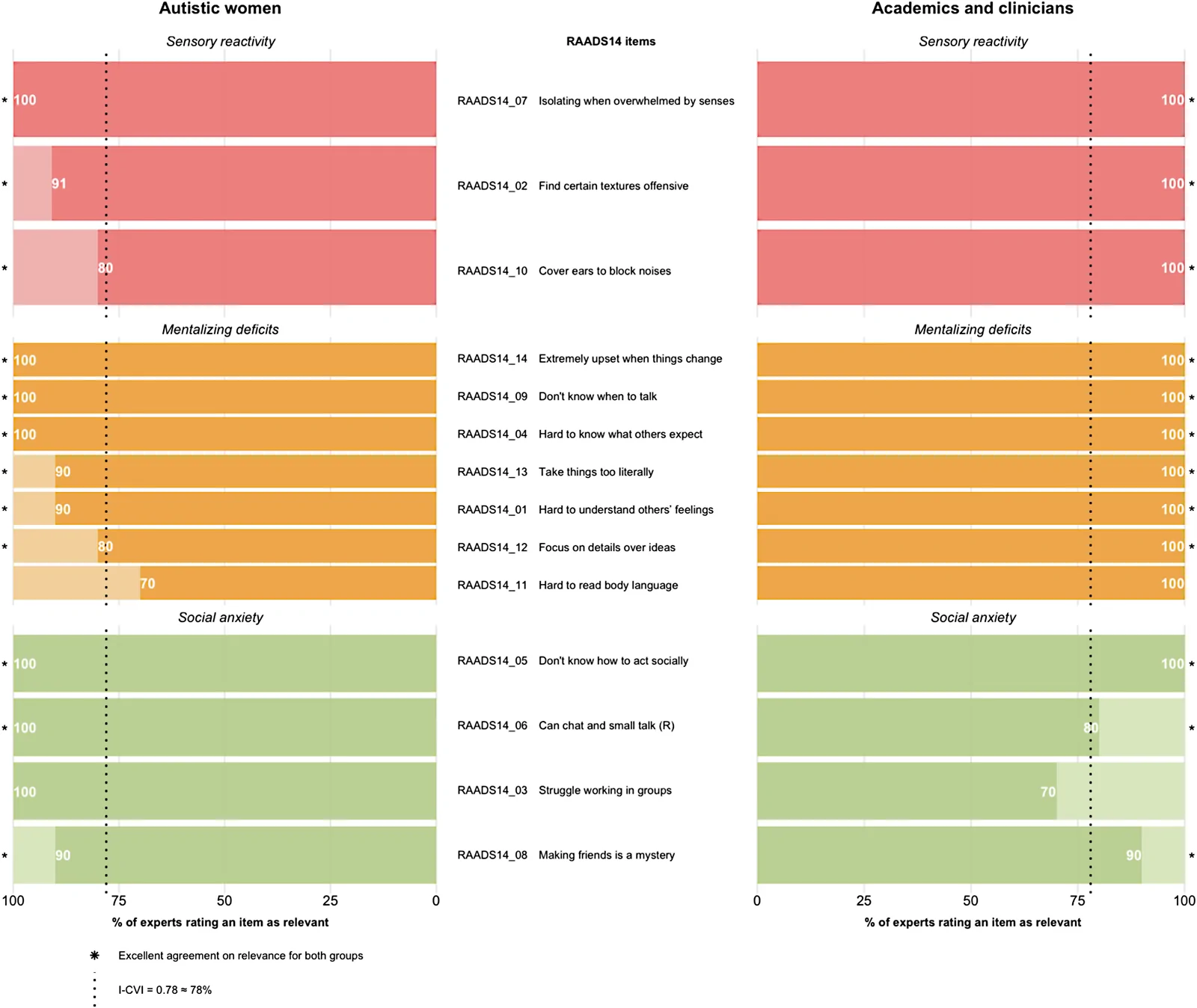
Percentage of autistic women (left) and academics/clinicians (right) rating each RAADS-14 item as being relevant (values correspond to I-CVIs × 100) to measuring autism in autistic women, per each factor. *Note.* Bars with * correspond to items that achieved excellent agreement on being relevant across both expert groups. Dotted vertical line represents I-CVI * ≥ *0.78 ≈ 78%, which corresponds to excellent agreement for modified kappa (Polit et al., 2007). Relevant items are those rated by experts as 1 (‘yes, it is relevant as is’) or 2 (‘yes, it is relevant but needs to be improved’). Non-relevant items are those rated as 3 (‘no, it is not relevant but it can be modified to be relevant’), 4 (‘no, it is not relevant to autistic women/to me’), or 5 (‘no, it is not relevant to autism at all’). (**R**) signifies a reverse scored statement

In total, 12 (85.7%) RAADS-14 items had excellent agreement on being relevant across both groups (see Fig. 6 and Table 2). Five items (35.71%) achieved full (100%) consensus across both autistic women and academic/clinician panels regarding their relevance, namely RAADS14 (*extremely upset when things change*), RAADS09 (*don’t know when to talk*), RAADS07 (*isolating when overwhelmed by senses*), RAADS05 (*don’t know how to act socially*), and RAADS04 (*hard to know what others expect*). Two items were considered relevant within one group but not the other. Specifically, only autistic women rated RAADS03 (*struggle working in groups*) as relevant, with a 30% difference in percentage relevance ratings between the groups. Conversely, academics/clinicians achieved consensus on RAADS11 (*hard to read body language*) being relevant, while autistic women did not, with the same 30% difference. Percentage differences in relevance ratings for all RAADS-14 items are available in Figure A5 of Additional File 1.

##### Domain-specific relevance

RAADS-14 included items mapped onto three constructs that correspond to core diagnostic criteria for autism. One construct related to the social-communication domain (*social anxiety* factor), while the other two aligned with RRBIs (*sensory reactivity* and *mentalizing deficits* factors). The majority of social-communication items (3/4) and nearly all RRBIs items (9/10) were rated as relevant.

### The most relevant items for measuring autism in autistic women

**Fig. 7:**
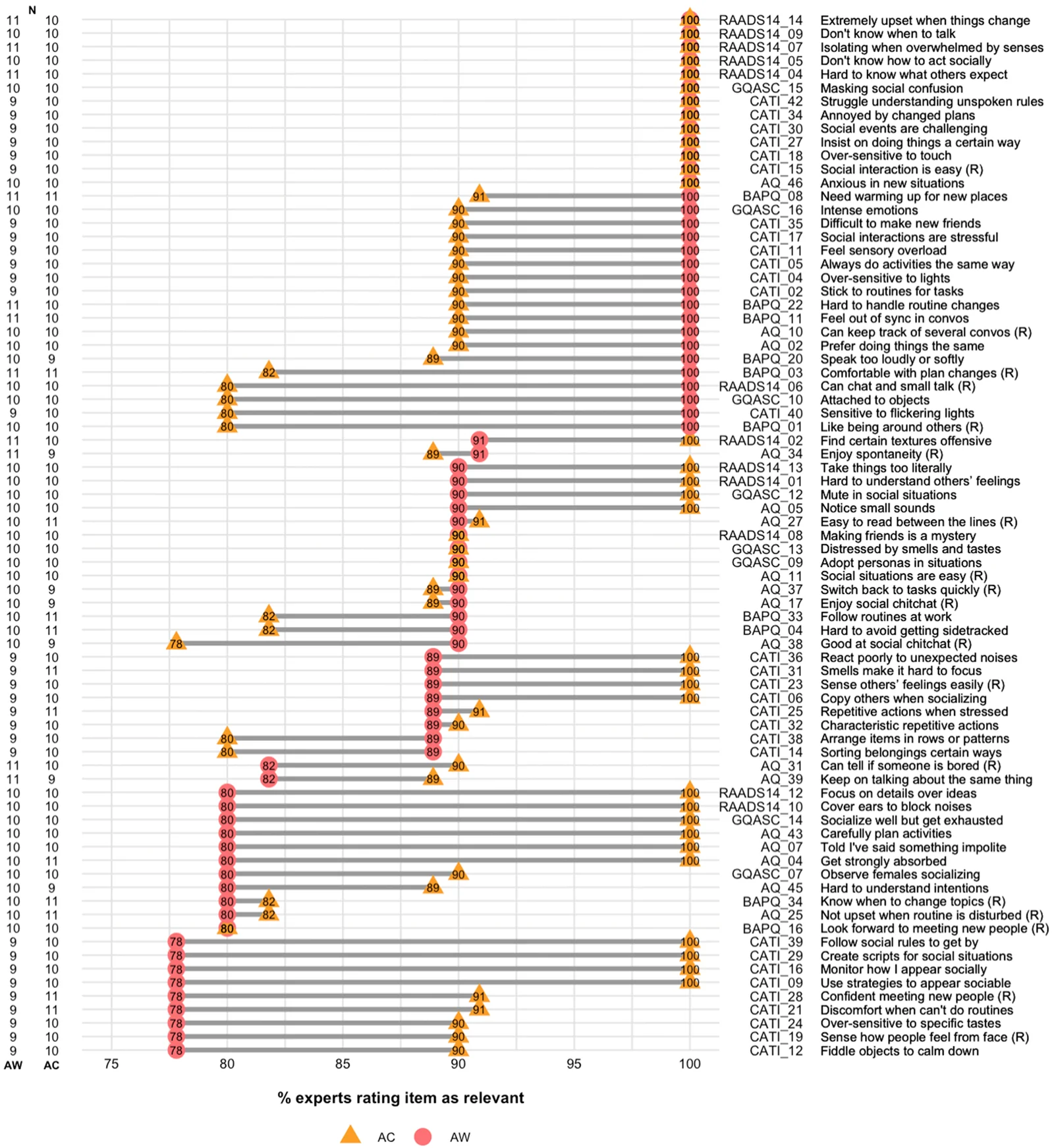
Items with excellent agreement on being relevant across two groups and questionnaires (values correspond to I-CVIs × 100). *Note.* AC: academics and clinicians, AW: autistic women, AQ: the Autism Spectrum Quotient, BAPQ: the Broad Autism Phenotype Questionnaire, CATI: the Comprehensive Autistic Trait Inventory, RAADS-14: the Ritvo Autism Asperger Diagnostic Scale 14, GQ-ASC: the Girls Questionnaire for Autism Spectrum Condition. (**R**) signifies a reverse scored statement

Across the five measures, which together comprised 163 items, 77 items (47.2%) reached excellent agreement on being relevant across both groups (see Fig. 7).

Thirteen items (RAADS14, RAADS09, RAADS07, RAADS05, RAADS04, GQASC15, CATI42, CATI34, CATI30, CATI27, CATI18, CATI15, AQ46) achieved complete (100%) agreement on being relevant across both panels. The items with the largest discrepancy in percentage relevance ratings between autistic women and academics/clinicians, each showing a 22% difference between groups, were CATI09 (*use strategies to appear sociable*), CATI16 (*monitor how I appear socially*), CATI29 (*create scripts for social situations*), and CATI39 (*follow social rules to get by*). These items were rated as less relevant by autistic women compared to academics/clinicians.

The majority of items that achieved consensus on being relevant reflected core diagnostic criteria for autism, based on the original factor assignment of each measure. Over half (*n* = 41; 53.25%) addressed RRBIs, while fewer items (*n* = 28; 36.36%) focused on social communication. The remaining 8 (10.39%) items captured autism-related traits outside the diagnostic criteria, namely masking (*n* = 7; 9.09%) and attention to detail (*n* = 1; 1.30%).

## Discussion

In this study, a modified Delphi method was used to reach consensus among autistic women and academics/clinicians with specialised expertise in autism in women, on the most relevant items from five existing measures of autistic traits: AQ, BAPQ, CATI, GQ-ASC, and RAADS-14.

Based on individual item ratings, we evaluated the relevance of each measure in its current form for autistic women. Autistic women and academics/clinicians broadly showed consistency in their evaluations of measures’ relevance at scale-level, with the same overall order of perceived relevance. That is, when the scale-level content validity indices were calculated and ranked in descending order for each group, both groups produced the same pattern of results, with RAADS-14 showing the highest relevance, followed by CATI and BAPQ, and AQ and GQ-ASC tied as the lowest. From these evaluations, a list of the most relevant items across the five measures was compiled, which could be further tested and integrated into existing instruments to enhance their relevance. The study also identified constructs such as masking as particularly valuable for inclusion, whereas imagination and attention to detail appeared less suitable for assessing autism more broadly or in more nuanced presentations of autistic traits. Nonetheless, all measures were found to incompletely capture aspects of experience relevant to autistic women, indicating construct underrepresentation that warrants further investigation.

### To what extent are the current measures relevant for assessing autistic traits in women?

#### Measures perceived as most relevant: RAADS-14 and CATI

Of the five measures, RAADS-14 was the only one to demonstrate excellent agreement on scale-level relevance by autistic women and academics/clinicians, with 12 of its 14 items rated as relevant, suggesting preliminary evidence of satisfactory content validity for autistic women.

However, despite the excellent content validity of the items, both groups indicated limited construct coverage, suggesting that the measure does not fully capture aspects of autism particularly relevant to women, such as masking, empathy, internal experiences, daily life skills, repetitive behaviours, and relationship challenges, previously identified in qualitative research as important for inclusion [75, 76, 104]. Qualitative feedback from the present study will further delineate which relevant experiences are not captured by the RAADS-14 and other evaluated measures, thereby informing the development of new items. These findings extend prior work by indicating that academics/clinicians similarly recognise these omissions, implying that experiences of potential clinical and research significance remain underrepresented. It is also possible that this construct underrepresentation partially reflects insufficient coverage of the autism subdomains outlined in the DSM-5, as a detailed review of the items reveals no content addressing, for example, repetitive interests. At the same time, the excellent agreement on RAADS-14’s relevance in this study contrasts with previous qualitative investigations, in which the measure was perceived as broadly not relevant to autistic women and those socialised as female [76], or as having questionable validity to autistic people [77]. This discrepancy likely reflects methodological differences, whereby the qualitative studies evaluated RAADS-14 more broadly alongside other measures, whereas in the present study, expert groups assessed items individually, from which scale-level content validity was derived, and included an independent evaluation of missing experiences. This item-level approach allowed for a clearer delineation of different aspects of content validity, suggesting that the previous perception of RAADS-14 as not relevant to autistic women may stem more from construct underrepresentation than from issues with individual item relevance, although further research is needed. The discrepancies across studies are indeed more plausibly attributable to differences between item-level and scale-level evaluations than to, for instance, sample composition. Notably, prior studies predominantly included cis autistic women [76] or individuals described as female (without specification of cis/trans identity) [77], which is broadly comparable to the present sample, comprising almost exclusively autistic cis women (with one participant not reporting this information). Additionally, prior research has identified discrepancies between open-ended and closed-ended responses when evaluating the relevance of autism measures for autistic women, with open-ended feedback often being perceived as more negative [74]. This pattern may also contribute to the discrepancies in findings and will be further examined using the accompanying qualitative data.

CATI received the second highest perceived relevance ratings and was evaluated relatively highly at the scale level by both groups, although only academics/clinicians reached excellent agreement on its relevance. This may reflect its greater perceived utility for clinical and research purposes, while also suggesting that some items may not fully capture autistic traits as experienced by autistic women or be as accessible to autistic thinking styles. Overall, CATI appears to be a promising measure, with 30 of 42 items achieving consensus across two groups on being relevant. On subscale level, a high proportion of RRBI-related items, especially those from the *sensory sensitivity* and *cognitive rigidity* (recently renamed to *cognitive flexibility* [69]) subscales, were rated as fully relevant. However, several items from the *repetitive behaviours* (renamed to *self-regulatory behaviours* [69]) subscale were identified for revision. Future refinements should focus primarily on items within the social communication domain, especially the *communication* subscale. The potential of CATI is further supported by consistent evidence of gender-related measurement invariance (non-bias) [43, 63, 69] and by the inclusion of a masking subscale, which is a domain absent from RAADS-14 but emphasised by autistic women as important for inclusion in the literature [76, 104]. However, it is important to note that the inclusion of masking items within measures of autistic traits remains debated as not all autistic individuals engage in masking [105]. Further research is needed to determine how such items should be approached, including their contribution to total score.

Both autistic women and academics/clinicians indicated construct underrepresentation, with aspects of autistic women’s experiences remaining unaddressed. As with the RAADS-14, no items capturing, for instance, repetitive interests, were identified, suggesting that this underrepresentation may partly reflect incomplete coverage of autism subdomains. With a revised version of CATI [64] now available, future research should assess whether the modifications have improved its content validity for autistic women, both at the item level and for the measure as a whole, given the observed discrepancies between item-level and overall-scale evaluations.

#### Measures requiring further refinement: BAPQ, GQ-ASC and AQ

Three of the five measures were associated with lower perceived relevance for autistic women in our study. All scales were also considered by our sample as exhibiting construct underrepresentation, which may reflect their limited ability to capture all experiences relevant to measuring autistic traits in women and/or insufficient coverage of the autism subdomains outlined in the DSM-5, both of which have been noted previously for BAPQ and AQ [76, 106]. These findings suggest limited content validity and warrant caution when using these measures to assess autism in individuals with more nuanced presentations of autistic traits.

BAPQ achieved an acceptable content validity index as rated by autistic women but was evaluated less favourably by professionals, with neither group reaching the threshold for excellent content validity. This difference may reflect the BAPQ’s original design to capture milder autistic traits typically observed in close relatives of autistic individuals, making it less clinically specific but more broadly representative of the autism phenotype, which may, in turn, make it more relatable to autistic women. Autistic women in a previous qualitative study perceived the BAPQ as broadly not relevant [76], a pattern reflected to a lesser extent among autistic women in the current study but more strongly among academics/clinicians. The differences in findings between the two studies are likely attributable to methodological variation, as noted in the case of RAADS-14. At the item level, both groups shared excellent agreement on 10 of 36 items as being relevant. Items assessing both social communication and RRBIs were identified as needing revision, particularly those related to social communication. Caution in its use is further warranted given the mixed evidence on the measure’s sex/gender-related measurement invariance [43, 43, 51], which requires additional investigation.

The AQ having one of the lowest scale-level relevance ratings among the evaluated measures is consistent with existing qualitative reports on its limited relevance to autistic women and individuals socialised as female [74–76]. However, in one of these studies, closed-ended responses suggested that the measure was somewhat relevant (3.60 on a five-point scale), contrasting with open-ended responses that indicated limited relevance [74]. The difference between that study’s close-ended ratings and our findings likely stems from methodological differences, with our item-level approach contrasting the prior study’s evaluation of the measure as a whole. In the present study, 17 of 50 items reached consensus on relevance across both groups, most of which related to RRBIs. In contrast, items from the *attention to detail* and *imagination* subscales were generally not rated as relevant, with the only *attention to detail* item considered relevant (*notice small sounds*) arguably also reflecting sensory sensitivities. This suggests that these domains may be less relevant for assessing autism in women, for autism more broadly, or that the items are not well formulated, an issue to be further examined through qualitative feedback. Previous research has consistently identified an *attention to detail* factor across AQ models [107]. In the context of our findings, this may indicate that while *attention to detail* is reliably part of the autism construct, it may be less relevant in more nuanced presentations of autistic traits or poorly captured for this presentation by the existing items. Future research should explore whether this factor emerges in factor analyses in samples composed exclusively of autistic women, women with high autistic traits, and/or those (of any gender) with more nuanced presentations of autistic traits. Supporting this, a study on gender-related item bias of different models of AQ (adjusted for age) found that eight *attention to detail* items were consistently biased (in at least 50% of the models present), with five more likely to be endorsed by men, with only one, also the sole relevant item in this study, consistently more likely endorsed by women [44]. With regards to the *imagination* items, *imagination* factor has not been consistently identified in prior factor-analytic studies of the AQ [107], suggesting that this domain may be less relevant for assessing autism more broadly or that better-formulated items are needed. Neither gender systematically endorsed a greater number of imagination items than the other [44], and the GQ-ASC likewise contained no items from its *imagination and play* subscale rated as relevant, further supporting the possibility of the limited value of the imagination construct in assessing autistic traits in either males or females.

GQ-ASC also demonstrated low scale-level content validity relative to the other evaluated measures. Overall, 8 of 21 items reached excellent agreement on relevance, primarily those assessing social communication, while items related to *imagination and play* were identified by the participants as candidates for revision or removal. Our findings align with qualitative reports highlighting the limited suitability of the GQ-ASC for adult women [74]. However, as with the AQ, discrepancies between closed- and open-ended responses have been noted in the literature: while closed-ended ratings indicated that the GQ-ASC was somewhat relevant (3.59 on a five-point scale), open-ended responses acknowledged its limitations yet perceived it as more suitable than the other measures (AQ and Camouflaging Autistic Traits Questionnaire [108]) assessed in the same study [74]. The authors of that study recommended re-evaluating the GQ-ASC’s appropriateness for assessing autistic traits, noting that non-autistic women, on average, scored above the suggested threshold and that the measure demonstrated high sensitivity but low specificity. This contrasts with the original validation study, which reported both high sensitivity and specificity [66], further underscoring the need for re-examining the measure’s validity. The current findings reinforce this recommendation.

### Which constructs are most relevant for evaluating autistic traits in women?

Across the five measures and 163 items, 77 items were identified as relevant, with 13 achieving full agreement across both groups. Although a higher proportion of RRBI-related items reached consensus on relevance compared with social communication items, closer examination revealed a general lack of items addressing restricted and repetitive interests. Only one item (*get strongly absorbed*), which reflects the qualitative aspect of such interests, was identified as relevant. This finding aligns with previous qualitative research in which autistic women and those socialised as female noted that existing measures inadequately capture the nature of their interests [76]. It further supports the view that apparent gender differences in restricted interests may partly reflect the insensitivity of current measures to more nuanced presentations of autistic traits [23]. In line with previous recommendations, new items should be developed to capture the nature, quality, and style of autistic women’s interests in ways that are more relevant and representative of their experiences. A similar implication applies to social communication items, where additional items are needed to better capture the nuanced social communication experiences of autistic women. This is important to ensure that the core domains of the autism construct are represented proportionately among the set of items identified as most relevant across the measures, thereby avoiding the under- or over-representation of either domain. Achieving this balance is important for establishing satisfactory content validity of a measure [71].

This study also identified one construct outside the core diagnostic criteria for autism, namely masking, as relevant for inclusion into measures of autistic traits. Interestingly, the greatest discrepancies in item ratings between the two groups were observed for masking items: academics/clinicians consistently rated them as highly relevant, reaching full consensus, whereas autistic women rated them lower. This difference may reflect that masking items were less relevant for autistic women who possibly do not personally engage in masking. Since not all autistic individuals mask [109], this supports the idea that such items may best serve as complementary indicators informing screening [76] rather than components of the core autistic trait score, as also reflected in recent discussions concerning the misuse of a widely used measure of camouflaging of autistic traits for diagnostic decision-making [105]. Including masking items in total autistic trait scores could potentially disadvantage autistic people who do not mask, as they may receive lower total scores relative to individuals with comparable score on core autistic traits who engage in masking. This risks conflating masking with higher levels of autistic traits, although masking does not necessarily indicate more pronounced autistic trait expression. Items relevant to masking may thus warrant separate scoring to contextualise responses on core traits, particularly when individuals score below clinical cut-off points. Further research is needed to determine whether masking may be better assessed within measures of broader lived experience rather than in screening tools. Consensus-based methods could help clarify how masking items should be integrated, especially given that autistic women and those socialised as female have identified them as important for inclusion, in previous research [76]. Furthermore, as the construct of autistic masking is still being conceptually refined and operationalised [105, 110, 111], further research is needed to develop autism-specific items that accurately capture this phenomenon.

### Future directions for the use and refinement of the items

The items identified as the most relevant for autistic women, particularly those with complete consensus, represent strong candidates for inclusion in a preliminary pool of items for future testing or for integration into existing measures to enhance their relevance for autistic women. The latter approach is especially encouraged, given that developing new instruments is resource-intensive and time-consuming, while recent years have seen a proliferation of autism trait measures. Accordingly, refining and enhancing existing tools may represent a more efficient and impactful direction for future work. Importantly, as noted in our previous work [76], we caution against using these items to develop gender-specific versions of autism measures, as doing so risks implying that all autistic women share the same presentation of autistic traits and could inadvertently exclude autistic people of other genders whose presentations are similarly more nuanced. Instead, future efforts should focus on ensuring a broad range of items that capture the diversity of presentations within subdomains of the autism construct, supported by a wide range of behavioural examples.

The items identified as relevant may be retained in their current form but should ideally be revised based on qualitative feedback, which constitutes the next phase of this study, supplemented with new items capturing experiences identified as missing from existing measures, and subsequently evaluated through different methods. These may include (Bayesian) formal content analysis [112] to ensure comprehensive and balanced coverage of all autism subdomains, cognitive interviewing to identify potential comprehension issues and explore alternative phrasings, and broader psychometric testing. The inclusion of items addressing relevant non-core autism constructs should, however, be guided by the intended purpose of the measure, as some aspects may be more appropriate for instruments assessing broader lived experiences rather than for screening or outcome measures [71, 76]. Following identification of such experiences, it may also be valuable to assess consensus among autistic women and academics/clinicians regarding the type of measure in which these items are best situated. While this recommendation has previously been made in relation to masking items, it remains necessary for other constructs as well.

### Limitations and future research

While this study focused on the relevance of existing measures for autistic women, the inclusion of autistic men and gender-diverse individuals would have enabled comparison of item relevance across groups. This would allow for clearer delineation of whether the identified limitations are specific to autistic women or generalisable across the broader autistic population, an issue that future studies should evaluate. As noted by a reviewer of this manuscript, it is also recommended that similar efforts be extended to parent-report measures of autistic traits used for screening of children, given recent evidence suggesting a catch-up in autism diagnoses among adolescent girls and adult women, presumably due to delays in referral and identification in early childhood [13]. Therefore, future investigation of measures used for autism screening in children is pivotal, given the delays experienced by autistic females. Finally, given emerging evidence of different developmental trajectories and genetic profiles associated with early versus late autism diagnoses [28], future research could also examine the relevance of such measures across these subgroups.

Although this study included an international sample of autistic women and academic/clinical experts, most participants were from Western countries, identified as White, and were highly educated. Accordingly, the findings may not generalise to non-Western contexts or to the full diversity of autistic women’s experiences. This may be particularly relevant for those with more complex or higher support needs, as well as for those at the intersection of racial minority identity and autism, whereby some concepts particularly salient to them (e.g., the compounded demands of masking associated with both racial minority identity and autism) may not have been taken into account. The professional expertise of clinicians also varied considerably, particularly in terms of the number of autistic women they had assessed, suggesting that ‘expert’ was not a fully homogeneous category in this study. This was further reflected in the decision to combine academics and clinicians into a single professional expert group despite differences in their respective backgrounds and education levels (with a PhD required for academics, whereas clinical experience was prioritised for clinicians). This decision was made to ensure equal weighting between the professional expert and lived-experience groups and to avoid having multiple professional subgroups outweigh the lived-experience perspectives. Future studies should re-evaluate the items with academic and clinical experts analysed separately, using larger samples of each and ensuring comparable numbers of autistic women to maintain balanced representation of perspectives. Furthermore, different participants completed different blocks of measures, which may have reduced consistency in evaluations. However, similar patterns were observed across blocks (e.g., consistently lower relevance ratings for imagination items and higher relevance ratings for masking items across measures assigned to different groups), suggesting acceptable consistency in evaluations.

While the inclusion of self-identifying autistic women was considered a strength, given the high rates of underdiagnosis, misdiagnosis, and late diagnosis among autistic women [4], their diagnostic status could not be independently verified and may have changed over time. Nonetheless, recent evidence supports the inclusion of self-identifying autistic individuals, as their perspectives and findings often align with those of formally diagnosed participants [69, 113].

Furthermore, in the survey administered to autistic women, reverse-coded items were reworded to directly reflect autistic traits rather than their opposites. It is possible that in some cases, this may have resulted in items being perceived as deficit-focused, potentially contributing to lower relevance ratings for those items. Finally, items were assigned to one of the two core domains of autism or to constructs outside the diagnostic criteria based on their original factor assignments. However, this approach was not ideal, as some items within the same factor could reasonably be interpreted as reflecting different domains. For example, in the RAADS-14, the items *get extremely upset when things change* and *don’t know when to talk* were both classified under the *mentalising deficits* factor, which was categorised as belonging to the RRBIs domain, although they could also be understood as reflecting RRBIs and social communication, respectively. It is therefore recommended that future research seek expert consensus on how individual items align with DSM-5 domains, ideally at the subdomain level, so that the distribution of relevant items across domains can be re-evaluated. Frequencies of responses provided in the supplementary materials may be used to support such re-examination.

## Conclusions

This study drew on the lived-expertise of autistic women and the professional expertise of academics and clinicians to reach consensus on the most relevant items (drawn from five existing measures) for assessing autistic traits in autistic women. It provides future scale-developers with a list of items that can be used to enhance the relevance of autism measures for this population. Although RAADS-14 and CATI were identified as the most relevant measures for autistic women, all five instruments showed limited content validity in terms of comprehensive construct coverage. Further research on the content validity of these measures is warranted, particularly in light of discrepancies between findings from closed-ended responses and qualitative reports from autistic women, and to more thoroughly identify experiences not captured by existing tools. By thoroughly documenting each step of the content validity evaluation, this study aims to enable scale-developers to build on these findings to refine existing questionnaires for individuals with more nuanced presentations of autistic traits and to promote collaborative progress in measure development.

## Electronic supplementary material

Below is the link to the electronic supplementary material.

Supplementary material 1

## Data Availability

Data supporting the findings of this study are provided in Additional File 1 as summary response frequencies rather than individual-level data, to ensure participant anonymisation.

## Abbreviations

AC: Academics/clinicians
AQ-10: Autism Spectrum Quotient-10
AQ-50: Autism Spectrum Quotient
AW: Autistic women
AWE: Autistic’s Women Experience
BAPQ: Broad Autism Phenotype Questionnaire
CATI: Comprehensive Autistic Trait Inventory
DSM: Diagnostic and Statistical Manual of Mental Disorders
GQ-ASC: Girls Questionnaire for Autism Spectrum Condition
I-CVI: Item-level content validity index
k*: Modified kappa statistic
NICE: National Institute for Health and Care Excellence
RAADS: Ritvo Autism & Asperger Diagnostic Scale
RRBIs: Restricted and repetitive patterns of behaviours and interests
S-CVI/Ave: Scale-level content validity index
UK: United Kingdom

## Footnotes

1 In the introduction, the term sex/gender is used because the described studies vary in whether they report sex or gender and frequently use these terms interchangeably. Although we acknowledge the conceptual distinction between sex and gender, this inconsistency in the literature necessitates the use of the combined term sex/gender throughout the introduction. Nevertheless, assessments of relevance in the present study focused specifically on autistic women and were therefore framed in terms of gender rather than biological sex.

2 Modified kappa, $${k^*} = {{I - CVI - {p_c}} \over {1 - {p_c}}}$$.

3 Probability of chance agreement, ${p_c} = \left[ {{{N!} \over {A!\left( {N - A} \right)!}}} \right]{0.5^N}$, where N represents the number of experts and A is the number who rated an item as relevant (options 1 or 2).

## References

[1] Happé F, Frith U. Annual research review: looking back to look forward – changes in the concept of autism and implications for future research. Child Phychol Psychiatry. 2020;61(3):218–32. 10.1111/jcpp.13176. 31994188

[2] American Psychiatric Association. Diagnostic and statistical manual of mental disorders: dSM-5TM. 5th. Arlington, VA, US: American Psychiatric Publishing, Inc; 2013.

[3] American Psychiatric Association. Diagnostic and statistical manual of mental disorders. Fifth Edition, Text Revision. Arlington, VA, US: American Psychiatric Publishing, Inc; 2022. p. DSM-5–TR.

[4] Lai MC, Baron-Cohen S. Identifying the lost generation of adults with autism spectrum conditions. Lancet Psychiatry. 2015;2(11):1013–27. 10.1016/S2215-0366(15)00277-1. 26544750

[5] Stewart GR, Happé F. Aging across the autism Spectrum. Annu Rev Dev Psychol. 2025;7(1):461–84. 10.1146/annurev-devpsych-111323-090813.

[6] Dell’osso L, Toschi D, Carpita B, Amatori G. Female autism: Grunya Sukhareva’s pioneering description reinterpreted in light of DSM-5-TR and the concept of camouflaging. Italian J Psychiatry. 2024;10(1). 10.36180/2421-4469-2024-1.

[7] Talantseva OI, Romanova RS, Shurdova EM, Dolgorukova TA, Sologub PS, Titova OS, et al. The global prevalence of autism spectrum disorder: a three-level meta-analysis. Front Psychiatry. 2023;14:1071181. 10.3389/fpsyt.2023.1071181. PMC9947250 36846240

[8] Zeidan J, Fombonne E, Scorah J, Ibrahim A, Durkin MS, Saxena S, et al. Global prevalence of autism: a systematic review update. Autism Res. 2022;15(5):778–90. 10.1002/aur.2696. PMC9310578 35238171

[9] Loomes R, Hull L, Mandy WPL. What is the male-to-female ratio in autism Spectrum disorder? A systematic review and meta-analysis. J Am Acad Child Psy. 2017, Jun;56(6):466–74. 10.1016/j.jaac.2017.03.013. 28545751

[10] Santomauro DF, Erskine HE, Herrera AMM, Miller PA, Shadid J, Hagins H, et al. The global epidemiology and health burden of the autism spectrum: findings from the global burden of Disease study 2021. Lancet Psychiatry. 2025;12(2):111–21. 10.1016/S2215-0366(24)00363-8. PMC11750762 39709974

[11] Harrop C, Tomaszewski B, Putnam O, Klein C, Lamarche E, Klinger L. Are the diagnostic rates of autistic females increasing? An examination of state-wide trends. Child Phychol Psychiatry. 2024;65(7):973–83. 10.1111/jcpp.13939. PMC11161335 38181181

[12] Russell G, Stapley S, Newlove-Delgado T, Salmon A, White R, Warren F, et al. Time trends in autism diagnosis over 20 years: a UK population-based cohort study. Child Phychol Psychiatry. 2022;63(6):674–82. 10.1111/jcpp.13505. 34414570

[13] Fyfe C, Winell H, Dougherty J, Gutmann DH, Kolevzon A, Marrus N, et al. Time trends in the male to female ratio for autism incidence: population based, prospectively collected, birth cohort study. BMJ. 2026;392:e084164. 10.1136/bmj-2025-084164. PMC12961995 41638711

[14] Maciver D, Singh Roy A, Johnston L, Boilson M, Curnow E, Johnstone-Cooke V, et al. Are we getting better at identifying and diagnosing neurodivergent girls and women? Insights into sex ratios and age of diagnosis from clinical population data in Scotland. Autism. 2026;30(2):375–89. 10.1177/13623613251383343. PMC12804396 41157911

[15] Fombonne E. Editorial: is autism overdiagnosed? Child Phychol Psychiatry. 2023;64(5):711–14. 10.1111/jcpp.13806. 37055375

[16] Lai MC, Lombardo MV, Auyeung B, Chakrabarti B, Baron-Cohen S. Sex/Gender differences and autism: setting the scene for future research. J Am Acad Child Psy. 2015;54(1):11–24. 10.1016/j.jaac.2014.10.003. PMC4284309 25524786

[17] Hull L, Petrides KV, Mandy W. The female autism Phenotype and camouflaging: a narrative review. Rev J Autism Dev Disord. 2020;7(4):306–17. 10.1007/s40489-020-00197-9.

[18] Van Wijngaarden-Cremers PJM, van Eeten E, Groen WB, Van Deurzen PA, Oosterling IJ, Van der Gaag RJ. Gender and age differences in the core Triad of impairments in autism Spectrum disorders: a systematic review and meta-analysis. J Autism Dev Disord. 2014;44(3):627–35. 10.1007/s10803-013-1913-9. 23989936

[19] Hull L, Mandy W, Petrides KV. Behavioural and cognitive sex/gender differences in autism spectrum condition and typically developing males and females. Autism. 2017;21(6):706–27. 10.1177/1362361316669087. 28749232

[20] Mahendiran T, Brian J, Dupuis A, Muhe N, Wong PY, Iaboni A, et al. Meta-analysis of sex differences in social and communication function in children with autism Spectrum disorder and attention-Deficit/Hyperactivity disorder. Front Psychiatry. 2019;10:804. 10.3389/fpsyt.2019.00804. PMC6844182 31749718

[21] Wood-Downie H, Wong B, Kovshoff H, Cortese S, Hadwin JA. Research review: a systematic review and meta-analysis of sex/gender differences in social interaction and communication in autistic and nonautistic children and adolescents. Child Phychol Psychiatry. 2021;62(8):922–36. 10.1111/jcpp.13337. 33137209

[22] Cruz S, Zubizarreta SCP, Costa AD, Araújo R, Martinho J, Tubío-Fungueiriño M, et al. Is there a bias towards males in the diagnosis of autism? A systematic review and meta-analysis. Neuropsychol Rev. 2025, Mar;35(1):153–76. 10.1007/s11065-023-09630-2. PMC11965184 38285291

[23] Edwards H, Wright S, Sargeant C, Cortese S, Wood-Downie H. Research review: a systematic review and meta-analysis of sex differences in narrow constructs of restricted and repetitive behaviours and interests in autistic children, adolescents, and adults. Child Phychol Psychiatry. 2024;65(1):4–17. 10.1111/jcpp.13855. 37485556

[24] Barnard-Brak L, Richman D, Almekdash MH. How many girls are we missing in ASD? An examination from a clinic- and community-based sample. AIA. 2019;5(3):214–24. 10.1108/AIA-11-2018-0048.

[25] Mazurek MO, Curran A, Burnette C, Sohl K. ECHO autism STAT: accelerating early access to autism diagnosis. J Autism Dev Disord. 2019;49(1):127–37. 10.1007/s10803-018-3696-5. 30043354

[26] Okoye C, Obialo-Ibeawuchi CM, Obajeun OA, Sarwar S, Tawfik C, Waleed MS, et al. Early diagnosis of autism Spectrum disorder: a review and analysis of the risks and benefits. Cureus. 2023;15(8):e43226. 10.7759/cureus.43226. PMC10491411 37692637

[27] Lacroix A, Tzang CC, Yu JX, Jacob BK, Alexandrovsky M, Winge-Breen A, et al. Disproportionate mental health risks in autistic females: a rapid review with quantitative and narrative syntheses. Front Neuroendocrinol. 2026;80:101229. 10.1016/j.yfrne.2025.101229. 41401863

[28] Zhang X, Grove J, Gu Y, Buus CK, Nielsen LK, Neufeld SAS, et al. Polygenic and developmental profiles of autism differ by age at diagnosis. Nature. 2025;646(8087):1146–55. 10.1038/s41586-025-09542-6. PMC12571882 41034588

[29] Martini MI, Kuja-Halkola R, Butwicka A, Du Rietz E, D’Onofrio BM, Happé F, et al. Sex differences in mental health problems and Psychiatric hospitalization in autistic young adults. JAMA Psychiatry. 2022;79(12):1188–98. 10.1001/jamapsychiatry.2022.3475. PMC9607919 36287538

[30] Smith JV, McQuaid GA, Wallace GL, Neuhaus E, Lopez A, Ratto AB, et al. Time is of the essence: age at autism diagnosis, sex assigned at birth, and psychopathology. Autism. 2024;28(11):2909–22. 10.1177/13623613241249878. PMC11496012 38725306

[31] Diemer MC, Ros-Demarize R, Bradley CC, Kanne S, Kim SH, Parish-Morris J, et al. Comparative analysis of autistic women across the Lifespan: childhood vs. Adulthood diagnosis. Adulthood Diagnosis. Autism Res. 2025;18(8):1651–63. 10.1002/aur.70073. PMC12384748 40546237

[32] Moitra M, Santomauro D, Degenhardt L, Collins PY, Whiteford H, Vos T, et al. Estimating the risk of suicide associated with mental disorders: a systematic review and meta-regression analysis. J Psychiatric Res. 2021;137:242–49. 10.1016/j.jpsychires.2021.02.053. PMC8095367 33714076

[33] Santomauro DF, Hedley D, Sahin E, Brugha TS, Naghavi M, Vos T, et al. The global burden of suicide mortality among people on the autism spectrum: a systematic review, meta-analysis, and extension of estimates from the global burden of Disease study 2021. Psychiatry Res. 2024;341:116150. 10.1016/j.psychres.2024.116150. 39197224

[34] Wechsler DL, Mandy W, Lai MC, Boyd B, Goodwin MS, Divan G, et al. Advancing health-care equity for autistic people: mental health as a key priority. Lancet. 2025;405(10491):1723–26. 10.1016/S0140-6736(25)00667-1. 40194537

[35] Punshon C, Skirrow P, Murphy G. The `not guilty verdict’. Autism. 2009;13(3):265–83. 10.1177/1362361309103795. 19369388

[36] Horn JL, McArdle JJ. A practical and theoretical guide to measurement invariance in aging research. Exp Aging Res. 1992;18(3):117–44. 10.1080/03610739208253916. 1459160

[37] Millsap RE, Meredith W. Factorial invariance: historical perspectives and new problems. In: Factor analysis at 100: historical developments and future directions. Mahwah, NJ, US: Lawrence Erlbaum Associates Publishers; 2007.

[38] Lai MC, Szatmari P. Sex and gender impacts on the behavioural presentation and recognition of autism. Curr Opin Psychiatry. 2020;33(2):117. 10.1097/YCO.0000000000000575. 31815760

[39] Pellicano E, den Houting J. Annual research review: shifting from ‘normal science’ to neurodiversity in autism science. Child Phychol Psychiatry. 2022;63(4):381–96. 10.1111/jcpp.13534. PMC9298391 34730840

[40] Baron-Cohen S, Wheelwright S, Skinner R, Martin J, Clubley E. The autism-Spectrum Quotient (AQ): evidence from Asperger Syndrome/High-functioning autism, males and females, scientists and mathematicians. J Autism Dev Disord. 2001;31(1):5–17. 10.1023/a:1005653411471. 11439754

[41] American Psychiatric Association. Diagnostic and statistical manual of mental disorders. 4th. Washington (DC): American Psychiatric Association; 1994.

[42] National Institute for Health and Care Excellence. Autism spectrum disorder in adults: diagnosis and management. 2021. Clinicial guideline no.: cG142. Available from: https://www.nice.org.uk/guidance/cg142. 32186834

[43] Waldren LH, Livingston LA, Shah P. Is there an optimal self-report measure to investigate autism-related sex differences? Res Autism. 2025;125:202617. 10.1016/j.reia.2025.202617.

[44] Belcher HL, Uglik-Marucha N, Vitoratou S, Ford RM, Morein-Zamir S. Gender bias in autism screening: measurement invariance of different model frameworks of the autism Spectrum Quotient. BJPsych Open. 2023;9(5):e173. 10.1192/bjo.2023.562. PMC10594186 37781848

[45] Allison C, Auyeung B, Baron-Cohen S. Toward brief “red flags” for autism screening: the short autism spectrum quotient and the short quantitative checklist in 1, 000 cases and 3, 000 controls. J Am Acad Child Psy. 2012;51(2):202–12.e7. 10.1016/j.jaac.2011.11.003. 22265366

[46] Murray AL, Allison C, Smith PL, Baron-Cohen S, Booth T, Auyeung B. Investigating diagnostic bias in autism spectrum conditions: an item response theory analysis of sex bias in the AQ-10. Autism Res. 2017;10(5):790–800. 10.1002/aur.1724. 27891820

[47] Murray AL, Booth T, Auyeung B, McKenzie K, Kuenssberg R. Investigating sex bias in the AQ-10: a replication study. Assessment. 2019;26(8):1474–79. 10.1177/1073191117733548. 28954527

[48] Eriksson JM, Andersen LM, Bejerot S. RAADS-14 screen: validity of a screening tool for autism spectrum disorder in an adult psychiatric population. Mol Autism. 2013;4(1):49. 10.1186/2040-2392-4-49. PMC3907126 24321513

[49] Ritvo RA, Ritvo ER, Guthrie D, Ritvo MJ, Hufnagel DH, McMahon W, et al. The Ritvo autism Asperger diagnostic scale-revised (RAADS-R): a scale to assist the diagnosis of autism Spectrum disorder in adults: an international validation study. J Autism Dev Disord. 2011;41(8):1076–89. 10.1007/s10803-010-1133-5. PMC3134766 21086033

[50] Hurley RSE, Losh M, Parlier M, Reznick JS, Piven J. The broad autism phenotype questionnaire. J Autism Dev Disord. 2007;37(9):1679–90. 10.1007/s10803-006-0299-3. 17146701

[51] Broderick N, Wade JL, Meyer JP, Hull M, Reeve RE. Model invariance across genders of the broad autism Phenotype Questionnaire. J Autism Dev Disord. 2015;45(10):3133–47. 10.1007/s10803-015-2472-z. 26063446

[52] Kingston SE. The factor structure of the broad autism phenotype questionnaire in a non-clinical sample of Canadian undergraduate students. 2024. Available from: https://mspace.lib.umanitoba.ca/server/api/core/bitstreams/f238e6ef-9dbb-48b6-bd19-a9ce8519474c/content [PhD thesis]. Winnipeg: The University of Manitoba.

[53] Sturm A, Huang S, Bal V, Schwartzman B. Psychometric exploration of the RAADS-R with autistic adults: implications for research and clinical practice. Autism. 2024;28(9):2334–45. 10.1177/13623613241228329. 38305196

[54] Robitzsch A, Lüdtke LOW. Why full, Partial, or Approximate measurement invariance are not a prerequisite for meaningful and valid group comparisons. Struct Equation Modeling: Multidiscip J. 2023;30(6):859–70. 10.1080/10705511.2023.2191292.

[55] Fischer R, Karl JA, Fontaine JRJ, Poortinga YH. Evidence of validity does not rule out systematic bias: a commentary on nomological noise and cross-Cultural invariance. Sociol Method Res. 2023;52(3):1420–37. 10.1177/00491241221091756.

[56] Meuleman B, Żółtak T, Pokropek A, Davidov E, Muthén B, Oberski DL, et al. Why measurement invariance is important in comparative research. A response to Welzel. Sociol Method Res. 2021;2023, 52(3):1401–19. 10.1177/00491241221091755.

[57] Welzel C, Brunkert L, Kruse S, Inglehart RF. Non-invariance? An overstated problem with misconceived causes. Sociol Method Res. 2023;52(3):1368–400. 10.1177/0049124121995521.

[58] Welzel C, Kruse S, Brunkert L. Against the Mainstream: on the limitations of non-invariance Diagnostics: response to Fischer et al. And Meuleman et al., et al. and Meuleman, Sociol Method Res. 2023;52(3):1438–55. 10.1177/00491241221091754 et al.

[59] Livingston LA, Leekam SR, Davies G, Shah P, Layinka O, Carrington SJ, et al. The signposting questionnaire for autism-2 (SQ-A-2): using input from autistic adults to inform questionnaire development. Mol Autism. 2026;17(1). 10.1186/s13229-026-00714-3. PMC13151257 41987220

[60] Ratto AB, Bascom J, DaVanport S, Strang JF, Anthony LG, Verbalis A, et al. Centering the inner experience of autism: development of the self-assessment of autistic traits. Autism Adulthood. 2023;5(1):93–105. 10.1089/aut.2021.0099. PMC10024271 36941856

[61] Kenworthy L, DaVanport S, Bascom J, Ratto A, Anthony LG, Khuu A, et al. Psychometric investigation of preliminary item-set for the self-assessment of autistic traits. Autism Adulthood. 2026;25739581251366048. 10.1177/25739581251366048.

[62] Abdallah SM, Fiorelli NM, Reardon KW, Poreh AM. Development of a Novel self-report tool to aid in the assessment of autism in Adulthood: the autism Spectrum trait scale. Autism Adulthood. 2025; 10.1089/aut.2024.0278.

[63] English MCW, Gignac GE, Visser TAW, Whitehouse AJO, Enns JT, Maybery MT. The Comprehensive autistic trait Inventory (CATI): development and validation of a new measure of autistic traits in the general population. Mol Autism. 2021;12(1):37. 10.1186/s13229-021-00445-7. PMC8130295 34001225

[64] Hechler FC, Tuomainen O, Weber N, Fahr F, Karlek B, Maroske M, et al. “What does ‘often’ even mean?” Revising and validating the comprehensive autistic trait Inventory in partnership with autistic people. Mol Autism. 2025;16(1):7. 10.1186/s13229-025-00643-7. PMC11803966 39915887

[65] Garau V, Murray AL, Woods R, Chown N, Hallett S, Murray F, et al. Development and validation of a novel self-report measure of Monotropism in autistic and non-autistic people: the Monotropism Questionnaire. 2023. (2025 Jun 19). . OSF [Preprint]. 10.31219/osf.io/ft73y. OSF [Preprint].

[66] Brown CM, Attwood T, Garnett M, Stokes SMAAI. Am I autistic? Utility of the girls questionnaire for autism spectrum condition as an autism assessment in adult women. Autism Adulthood. 2020;2(3):216–26. 10.1089/aut.2019.0054. PMC8992859 36601442

[67] Ortas de Haro E, Ayllón-Negrillo, Ayllón-Negrillo E. FEM-A Questionnaire: assessment tool for level 1 autism in women. Educ Sci. 2025;15(9):1242. 10.3390/educsci15091242.

[68] Groen Y, Ebert WM, Dittner FM, Stapert AF, Henning D, Greaves-Lord K, et al. Measuring the autistic Women’s experience (AWE). IJERPH. 2023;20(24):7148. 10.3390/ijerph20247148. PMC10742621 38131700

[69] English MC, Poulsen RE, Maybery MT, McAlpine D, Sowman PF, Pellicano E. Psychometric evaluation of the Comprehensive autistic trait Inventory in autistic and non-autistic adults. Autism. 2025;13623613251347740. 10.1177/13623613251347740. PMC12618716 40671261

[70] Fischer R, Karl JA, Luczak-Roesch M, Hartle L. Why we need to rethink measurement invariance: the role of measurement invariance for cross-Cultural research. Cross-Cult Res. 2025;59(2):147–79. 10.1177/10693971241312459.

[71] Haynes SN, Richard DCS, Kubany ES. Content validity in psychological assessment: a functional approach to concepts and methods. Psychological Assess. 1995;7(3):238–47. 10.1037/1040-3590.7.3.238.

[72] Mokkink LB, Elsman EBM, Terwee CB. COSMIN guideline for systematic reviews of patient-reported outcome measures version 2.0. Qual Life Res. 2024;33(11):2929–39. 10.1007/s11136-024-03761-6. PMC11541334 39198348

[73] Koller I, Levenson MR, Glück J. What do You think You are measuring? A mixed-methods procedure for assessing the content validity of test items and theory-based scaling. Front Psychol. 2017;8:8. 10.3389/fpsyg.2017.00126. PMC5318383 28270777

[74] Parsons CH, Jackson MC. “These questions are geared towards male autistics, not female … I found they never quite reflected my experience”: assessing the efficacy of autism questionnaires in identifying adult autistic women. PsyArXiv [Preprint]. 2025. [2025 Apr 2]. https://osf.io/preprints/psyarxiv/k4ey5_v2.

[75] Chow CKC, Lam GYH. “I thought about whether my answers represent What I Am like”: Hong Kong autistic females’ perspectives on autism Spectrum Quotient (AQ). Qual Health Res. 2025;10497323251343773. 10.1177/10497323251343773. 40574537

[76] Uglik-Marucha N, Show S, Vitoratou S, Happé F, Belcher H. ‘I fit the category of the box, it just doesn’t describe me well.’ exploring the perspectives of autistic women and gender-diverse individuals on self-report autism measures. PLoS One. 2026;21(1):e0337600. 10.1371/journal.pone.0337600. PMC12803452 41533668

[77] Stacey R, Cage E. “Simultaneously vague and oddly specific”: understanding autistic People’s experiences of decision making and research questionnaires. Autism Adulthood. 2023;5(3):263–74. 10.1089/aut.2022.0039. PMC10468547 37663447

[78] Hsu CC, Sandford BA. The Delphi technique: making sense of consensus. Practical Assess, Res, Evaluation. 2007;12(10). 10.7275/pdz9-th90.

[79] Kis A, Marci T, Altoè G, Azevedo F, Tur E, Houkes W, et al. Academic research values scale: item selection and content validity. Preprint. 2024. [2025 Apr 3]. OSF. https://osf.io/preprints/psyarxiv/c4dux_v1.

[80] McMillan SS, King M, Tully MP. How to use the nominal group and Delphi techniques. Int J Clin Pharm. 2016;38(3):655–62. 10.1007/s11096-016-0257-x. PMC4909789 26846316

[81] Taze D, Hartley C, Morgan AW, Chakrabarty A, Mackie SL, Griffin KJ. Developing consensus in Histopathology: the role of the Delphi method. Histopathology. 2022;81(2):159–67. 10.1111/his.14650. PMC9541891 35322456

[82] Delbecq AL, Van de Ven AH, David G. Group techniques for program Planning; a guide to nominal group and Delphi processes. Glenview IL: Scott Foresman and Company; 1975.

[83] Fink A, Kosecoff J, Chassin M, Brook RH. Consensus methods: characteristics and guidelines for use. Am J Public Health. 1984;74(9):979–83. 10.2105/ajph.74.9.979. PMC1651783 6380323

[84] Hasson F, Keeney S, McKenna H. Research guidelines for the Delphi survey technique. J Adv Nurs. 2000;32(4):1008–15. 10.1046/j.1365-2648.2000.t01-1-01567.x. 11095242

[85] de Villiers MR, de Villiers Pjt, Kent AP, de Villiers PJT. The Delphi technique in health sciences education research. Med Teach. 2005;27(7):639–43. 10.1080/13611260500069947. 16332558

[86] Cumin J, Pelaez S, Mottron L. Positive and differential diagnosis of autism in verbal women of typical intelligence: a Delphi study. Autism. 2022;26(5):1153–64. 10.1177/13623613211042719. PMC9340131 34514874

[87] Kok FM. The female side of ADHD and ASD. 2021. Available from: 10.33612/diss.179073089 [PhD thesis]. Groningen: University of Groningen.

[88] Lovelace TS, Comis MP, Tabb JM, Oshokoya OE. Missing from the narrative: a seven-decade scoping review of the inclusion of black autistic women and girls in autism research. Behav Anal Pract. 2022;15(4):1093–105. 10.1007/s40617-021-00654-9. PMC9745006 36605161

[89] Malone KM, Pearson JN, Palazzo KN, Manns LD, Rivera AQ, Mason Martin DL. The scholarly neglect of black autistic adults in autism research. Autism Adulthood. 2022;4(4):271–80. 10.1089/aut.2021.0086. PMC9908289 36777371

[90] Constantino JN, Gruber CP. Torrance, CA: Western Psychological Services; 2012 Social Responsiveness Scale-Second Edition (SRS-2).

[91] Stewart ME, Austin EJ. The structure of the autism-Spectrum Quotient (AQ): evidence from a student sample in Scotland. Pers Indiv Differ. 2009;47(3):224–28. 10.1016/j.paid.2009.03.004.

[92] Russell-Smith SN, Maybery MT, Bayliss DM. Relationships between autistic-like and schizotypy traits: an analysis using the autism Spectrum Quotient and Oxford-liverpool Inventory of feelings and experiences. Pers Indiv Differ. 2011;51(2):128–32. 10.1016/j.paid.2011.03.027.

[93] Freeth M, Sheppard E, Ramachandran R, Milne E. A cross-Cultural comparison of autistic traits in the UK, India and Malaysia. J Autism Dev Disord. 2013;43(11):2569–83. 10.1007/s10803-013-1808-9. 23494561

[94] Lau WYP, Gau SSF, Chiu YN, Wu YY, Chou WJ, Liu SK, et al. Psychometric properties of the Chinese version of the autism Spectrum Quotient (AQ). Res Dev Disabil. 2013;34(1):294–305. 10.1016/j.ridd.2012.08.005. 22985783

[95] Lau WYP, Kelly AB, Peterson CC. Further evidence on the Factorial structure of the autism Spectrum Quotient (AQ) for adults with and without a clinical diagnosis of autism. J Autism Dev Disord. 2013;43(12):2807–15. 10.1007/s10803-013-1827-6. 23619950

[96] Bottini S, Conine DE. Strategies to prevent and detect fraudulent responses in online research: a cautionary tale and tutorial. Behav Anal Pract. 2026;19(2):845–55. 10.1007/s40617-026-01163-3. PMC13275931 42327400

[97] Pellicano E, Adams D, Crane L, Hollingue C, Allen C, Almendinger K, et al. Letter to the editor: a possible threat to data integrity for online qualitative autism research. Autism. 2024;28(3):786–92. 10.1177/13623613231174543. 37212144

[98] Brookes ST, Chalmers KA, Avery KNL, Coulman K, Blazeby JM. Impact of question order on prioritisation of outcomes in the development of a core outcome set: a randomised controlled trial. Trials. 2018;19(1):66. 10.1186/s13063-017-2405-6. PMC5784591 29370827

[99] Polit DF, Beck CT, Owen SV. Is the CVI an acceptable indicator of content validity? Appraisal and recommendations. Res Nurs Health. 2007;30(4):459–67. 10.1002/nur.20199. 17654487

[100] Cicchetti DV, Sparrow SA. Developing criteria for establishing interrater reliability of specific items: applications to assessment of adaptive behavior. Am J Ment Defic. 1981;86(2):127–37. 7315877

[101] Fleiss JL. Statistical methods for rates and proportions. Second. New York: John Wiley & Sons; 1981.

[102] Waltz CF, Strickland OL, Lenz ER, Satyshur RD, Stone KS, et al. Measurement in Nursing and health research. 5th. New York: Springer Publishing Company; 2016.

[103] Keyes D. How to make polished population pyramids in ggplot: part 1 [Internet]. R for the rest of us. 2024. [2025 Jan 8]. https://rfortherestofus.com/2024/07/population-pyramid-part-1.

[104] Trundle G, Chester V, Alexander Z, Morris P, Crump I. Guidelines to Assist in autism diagnostic assessments with females: a Delphi study. Autism Adulthood. 2025; 10.1089/aut.2024.0325.

[105] Hannon BR, Hull L, Lai MC, Magiati I, Mandy W. The use and misuse of the camouflaging autistic traits Questionnaire in autism research and clinical practice: issues, considerations, and suggestions. Autism Adulthood. 2026; 10.1177/25739581261435868.

[106] Godoy-Giménez M, González-Rodríguez A, Cañadas F, Estévez AF, Sayans-Jiménez P. Is it possible to assess the two-domain definition of the Broad autism Phenotype using the available measurement tools? J Autism Dev Disord. 2022;52(7):2884–95. 10.1007/s10803-021-05158-7. PMC9213296 34185239

[107] English MCW, Gignac GE, Visser TAW, Whitehouse AJO, Maybery MT. A comprehensive psychometric analysis of autism-spectrum quotient factor models using two large samples: Model recommendations and the influence of divergent traits on total-scale scores. Autism Res. 2020;13(1):45–60. 10.1002/aur.2198. 31464106

[108] Hull L, Mandy W, Lai MC, Baron-Cohen S, Allison C, Smith P, et al. Development and validation of the camouflaging autistic traits Questionnaire (CAT-Q). J Autism Dev Disord. 2019;49(3):819–33. 10.1007/s10803-018-3792-6. PMC6394586 30361940

[109] Chapman L, Rose K, Hull L, Mandy W. “I want to fit in… but I don’t want to change myself fundamentally”: a qualitative exploration of the relationship between masking and mental health for autistic teenagers. Res Autism Spectr Disord. 2022;99:102069. 10.1016/j.rasd.2022.102069.

[110] Lai MC, Hull L, Mandy W, Chakrabarti B, Nordahl CW, Lombardo MV, et al. Commentary: ‘camouflaging’ in autistic people – reflection on Fombonne (2020). Child Phychol Psychiatry. 2021;62(8). 10.1111/jcpp.13344. 33289092

[111] Williams ZJ. Commentary: the construct validity of ‘camouflaging’ in autism: psychometric considerations and recommendations for future research - reflection on Lai et al. (2020). Child Phychol Psychiatry. 2022;63(1):118–21. 10.1111/jcpp.13468. PMC8678389 34145574

[112] Spoto A, Nucci M, Prunetti E, Vicovaro M. Improving content validity evaluation of assessment instruments through formal content validity analysis. Psychological Methods. 2025;30(2):203–22. 10.1037/met0000545. 36595444

[113] Ardeleanu K, Steinberg H, Garfield T, Voltaire S, Shea L, Brown M, et al. Self-identification of autism: why some autistic adults lack a clinical diagnosis and why this matters for inclusion. Autism. 2025;29(9):2344–55. 10.1177/13623613241297222. 39552426

