# Supplementary material for: Are items from current self-report measures adequate for assessing autistic traits in women? Insights from a modified Delphi study with autistic women and professional experts
Source: Mol Autism. 2026 Jul 17;17:41. doi: 10.1186/s13229-026-00724-1 (PMC13663005; doi:10.1186/s13229-026-00724-1)
Supplement: Supplementary file 1 [file 13229_2026_724_MOESM1_ESM.pdf]

Additional File 1

Table A1

Characteristics of the academic experts (N=18)

Characteristic		Frequency
**Academic/Research position**	Lecturer, Assistant Professor, or equivalent senior researcher (%)	6 (33.3)
	Research fellow, Research associate, Postdoctoral researcher (%)	5 (27.8)
	Senior Lecturer or Reader, Associate Professor, or equivalent senior research position (%)	3 (16.7)
	Professor, or equivalent senior research position (%)	1 (5.6)
	Other academic position (%)	3 (16.7)
**Academic background**	Biomedical sciences (%)	1 (5.6)
	Psychology (%)	15 (83.3)
	Education (%)	1 (5.6)
	Genetics (%)	1 (5.6)
	Medicine (%)	2 (11.1)
	Neuroscience (%)	4 (22.2)
	Other academic background (%)	4 (22.2)
**Years of autism research experience**	Mean (SD)	11.4 (6.4)
	Median (IQR)	9 (6.5)
	Range	5 - 24
**Number of published articles on autistic women and/or gender differences in autism**	Mean (SD)	4.2 (3.4)
	Median (IQR)	4 (1.8)
	Range	1 - 16
**Secondary clinical work**	(%)	8 (44.4)
Clinical background	Assistant psychologist (%)	1 (12.5)
	Speech and language therapist (%)	2 (25.0)

	Clinical psychologist (%)	5 (62.5)
	Registered nurse (%)	1 (12.5)

Note. SD: standard deviation; IQR: interquartile range. Due to a rounding error, some columns may total 99.9% or 100.1% instead of 100%.

Table A2

Characteristics of the clinical experts (N=15)

Characteristic		Frequency
**Clinical background**	Assistant psychologist or neurodevelopmental worker/specialist (%)	1 (6.7)
	Speech and language therapist (%)	1 (6.7)
	Clinical psychologist (%)	6 (40.0)
	Occupational therapist (%)	1 (6.7)
	Psychiatrist (%)	2 (13.3)
	Registered nurse (%)	3 (20.0)
	Other professional background (%)	2 (13.3)
**Clinical setting**	Autism assessment service (%)	11 (73.3)
	National specialist service (%)	2 (13.3)
	Criminal Justice System (%)	1 (6.7)
	Specialist service for intellectual disability (%)	1 (6.7)
	Independent practice (%)	4 (26.7)
	Inpatient ward (%)	1 (6.7)
	Other clinical setting (%)	3 (20.0)
**Age group worked with**	Adults (%)	9 (60.0)
	Lifespan (%)	6 (40.0)
**Years of experience diagnosing autism in adults**	Mean (SD)	10.8 (4.2)
	Median (IQR)	11 (5.5)
	Range	5 - 20
**Number of cases of women assessed for autism**	51-100 (%)	2 (13.3)
	101-150 (%)	5 (33.3)
	151-200 (%)	2 (13.3)
	201-250 (%)	0 (0.0)
	251-300 (%)	1 (6.7)

	301-350 (%)	2 (13.3)
	>400 (%)	3 (20.0)
**Secondary academic work**	(%)	5 (33.3)
	Research fellow, Research associate, Postdoctoral researcher (%)	2 (40.0)
	Senior Lecturer or Reader, Associate Professor, or equivalent senior research position (%)	1 (20.0)
	Other position (%)	2 (40.0)

Note. SD: standard deviation; IQR: interquartile range. Due to a rounding error, some columns may total 99.9% or 100.1% instead of 100%.

Table A3

Reworded reverse-scored statements presented to autistic women in the survey

Scale	Item	Original (reverse scored) statements	Reworded reverse-scored statements
AQ	AQ01	I prefer to do things with others, rather than on my own	I prefer to do things on my own, rather than with others
	AQ03	If I try to imagine something, I find it very easy to create a picture in my mind	If I try to imagine something, I don't find it easy to create a picture in my mind
	AQ08	When I'm reading a story, I can easily imagine what the characters might look like	When I'm reading a story, I find it difficult to imagine what the characters might look like
	AQ10	In a social group, I can easily keep track of several different people's conversations	In a social group, I struggle to keep track of several different people's conversations
	AQ11	I find social situations easy	I find social situations difficult
	AQ14	I find making up stories easy	I find making up stories difficult
	AQ15	I find myself drawn more strongly to people than to things	I find myself drawn more strongly to things than to people
	AQ17	I enjoy social chitchat	I don't enjoy social chitchat
	AQ24	I would rather go to the theatre than to a museum	I would rather go to a museum than to the theatre
	AQ25	It does not upset me if my daily routine is disturbed	It upsets me if my daily routine is disturbed

	AQ27	I find it easy to 'read between the lines' when someone is talking to me	I find it difficult to 'read between the lines' when someone is talking to me
	AQ28	I usually concentrate more on the whole picture, rather than on the small details	I usually concentrate more on the small details, rather than on the whole picture
	AQ29	I am not very good at remembering phone numbers	I am very good at remembering phone numbers
	AQ30	I don't usually notice small changes in a situation or a person's appearance	I usually notice small changes in a situation or a person's appearance
	AQ31	I know how to tell if someone listening to me is getting bored	I don't know how to tell if someone listening to me is getting bored
	AQ32	I find it easy to do more than one thing at once	I find it difficult to do more than one thing at once
	AQ34	I enjoy doing things spontaneously	I don't enjoy doing things spontaneously
	AQ36	I find it easy to work out what someone is thinking or feeling just by looking at their face	I find it difficult to work out what someone is thinking or feeling just by looking at their face
	AQ37	If there is an interruption, I can switch back to what I was doing very quickly	If there is an interruption, I can't switch back to what I was doing very quickly
	AQ38	I am good at social chitchat	I am not good at social chitchat
	AQ40	When I was young, I used to enjoy playing games involving pretending with other children	When I was young, I did not enjoy playing games involving pretending with other children
	AQ44	I enjoy social occasions	I don't enjoy social occasions
	AQ47	I enjoy meeting new people	I don't enjoy meeting new people
	AQ48	I am a good diplomat	I am not a good diplomat
	AQ49	I am not very good at remembering people's date of birth	I am very good at remembering people's date of birth
	AQ50	I find it very easy to play games with children that involve pretending	I don't find it very easy to play games with children that involve pretending.
BAPQ	BAPQ01	I like being around other people	I don't like being around other people
	BAPQ03	I am comfortable with unexpected changes in plans	I am not comfortable with unexpected changes in plans

	BAPQ07	I am ‘‘in-tune’’ with the other person during conversation	I am not ‘‘in-tune’’ with the other person during conversation
	BAPQ09	I enjoy being in social situations	I don't enjoy being in social situations
	BAPQ12	People find it easy to approach me	People find it difficult to approach me
	BAPQ15	I am flexible about how things should be done	I am not flexible about how things should be done
	BAPQ16	I look forward to situations where I can meet new people	I dread situations where I can meet new people
	BAPQ19	I look forward to trying new things	I dread trying new things
	BAPQ21	I can tell when someone is not interested in what I am saying	I can't tell when someone is not interested in what I am saying
	BAPQ23	I am good at making small talk	I am not good at making small talk
	BAPQ25	I feel like I am really connecting with other people	I don't feel like I am really connecting with other people
	BAPQ28	I am warm and friendly in my interactions with others	I am not warm and friendly in my interactions with others
	BAPQ30	I alter my daily routine by trying something different	I stick to my daily routine and avoid trying something different
	BAPQ34	I can tell when it is time to change topics in conversation	I can't tell when it is time to change topics in conversation
	BAPQ36	I enjoy chatting with people	I don't enjoy chatting with people
CATI	CATI08	I generally enjoy social events	I don't generally enjoy social events
	CATI15	Social interaction is easy for me	Social interaction is difficult for me
	CATI19	I can tell how people feel from their facial expressions	I cannot tell how people feel from their facial expressions
	CATI23	I find it easy to sense what someone else is feeling	I find it difficult to sense what someone else is feeling
	CATI28	I feel confident or capable when meeting new people	I don't feel confident or capable when meeting new people
GQ-ASC	GQASC01	I enjoy fantasy worlds	I don't enjoy fantasy worlds
	GQASC02	I am interested in fiction	I am not interested in fiction

	GQASC03	When I was 5-12 years old, I played as imaginatively as other girls	When I was 5-12 years old, I didn't play as imaginatively as other girls
	GQASC04	When I was 5-12 years old, I had imaginary friends or imaginary animals	When I was 5-12 years old, I didn't have imaginary friends or imaginary animals
	GQASC05	When I was 5-12 years old, I created my own complex 'set ups' with toys	When I was 5-12 years old, I didn't create my own complex 'set ups' with toys
	GQASC18	When I was 5-12 years old, I preferred to play with girls' toys	When I was 5-12 years old, I did not prefer to play with girls' toys
RAADS-14	RAADS06	I can chat and make small talk with people	I struggle to chat and make small talk with people.

Note. AQ: Autism Spectrum Quotient, BAPQ: Broad Autism Phenotype Questionnaire, CATI: The Comprehensive Autistic Trait Inventory, GQ-ASC: the Girls Questionnaire for Autism Spectrum Condition, RAADS-14: Ritvo Autism Asperger Diagnostic Scale 14.

Table A4

Frequencies of response options for AQ relevance evaluations across academics, clinicians, and autistic women

Item	Label	Factor	Expert group	N	Yes, it is relevant as it is (%)	Yes, it is relevant but needs to be improved (%)	No, it is not relevant but can be modified to be relevant (%)	No, it is not relevant to autistic women / to me (%)	No, it is not relevant to autism at all (%)
AQ01	I prefer to do things with others, rather than on my own (R)	Social skills	AC	9	4 (44.44)	1 (11.11)	1 (11.11)	2 (22.22)	1 (11.11)
			AW	10	6 (60.00)	1 (10.00)	3 (30.00)	0 (0.00)	0 (0.00)
AQ02	I prefer to do things the same way over and over again	Attention switching	AC	10	7 (70.00)	2 (20.00)	1 (10.00)	0 (0.00)	0 (0.00)
			AW	10	7 (70.00)	3 (30.00)	0 (0.00)	0 (0.00)	0 (0.00)
AQ03	If I try to imagine something, I find it very easy to create a picture in my mind (R)	Imagination	AC	11	3 (27.27)	2 (18.18)	1 (9.09)	1 (9.09)	4 (36.36)
			AW	10	5 (50.00)	0 (0.00)	0 (0.00)	4 (40.00)	1 (10.00)
AQ04	I frequently get so strongly absorbed in one thing that I lose sight of other things	Attention switching	AC	11	10 (90.91)	1 (9.09)	0 (0.00)	0 (0.00)	0 (0.00)
			AW	10	8 (80.00)	0 (0.00)	1 (10.00)	0 (0.00)	1 (10.00)
AQ05	I often notice small sounds when others do not	Attention to detail	AC	10	8 (80.00)	2 (20.00)	0 (0.00)	0 (0.00)	0 (0.00)
			AW	10	8 (80.00)	1 (10.00)	0 (0.00)	1 (10.00)	0 (0.00)
AQ06			AC	9	2 (22.22)	3 (33.33)	1 (11.11)	3 (33.33)	0 (0.00)

Item	Label	Factor	Expert group	N	Yes, it is relevant as it is (%)	Yes, it is relevant but needs to be improved (%)	No, it is not relevant but can be modified to be relevant (%)	No, it is not relevant to autistic women / to me (%)	No, it is not relevant to autism at all (%)
	I usually notice car number plates or similar strings of information	Attention to detail	AW	11	5 (45.45)	1 (9.09)	1 (9.09)	3 (27.27)	1 (9.09)
AQ07	Other people frequently tell me that what I've said is impolite, even though I think it is polite	Communication	AC	10	4 (40.00)	6 (60.00)	0 (0.00)	0 (0.00)	0 (0.00)
			AW	10	6 (60.00)	2 (20.00)	0 (0.00)	2 (20.00)	0 (0.00)
AQ08	When I'm reading a story, I can easily imagine what the characters might look like (R)	Imagination	AC	10	2 (20.00)	1 (10.00)	1 (10.00)	4 (40.00)	2 (20.00)
			AW	10	5 (50.00)	0 (0.00)	1 (10.00)	3 (30.00)	1 (10.00)
AQ09	I am fascinated by dates	Attention to detail	AC	10	1 (10.00)	2 (20.00)	2 (20.00)	2 (20.00)	3 (30.00)
			AW	10	1 (10.00)	0 (0.00)	0 (0.00)	8 (80.00)	1 (10.00)
AQ10	In a social group, I can easily keep track of several different people's conversations (R)	Attention switching	AC	10	6 (60.00)	3 (30.00)	0 (0.00)	0 (0.00)	1 (10.00)
			AW	10	8 (80.00)	2 (20.00)	0 (0.00)	0 (0.00)	0 (0.00)
AQ11	I find social situations easy (R)	Social skills	AC	10	8 (80.00)	1 (10.00)	0 (0.00)	0 (0.00)	1 (10.00)
			AW	10	8 (80.00)	1 (10.00)	1 (10.00)	0 (0.00)	0 (0.00)
AQ12	I tend to notice details that others do not	Attention to detail	AC	10	7 (70.00)	0 (0.00)	0 (0.00)	2 (20.00)	1 (10.00)
			AW	10	8 (80.00)	1 (10.00)	0 (0.00)	1 (10.00)	0 (0.00)
AQ13	I would rather go to a library than to a party	Social skills	AC	11	3 (27.27)	2 (18.18)	0 (0.00)	2 (18.18)	4 (36.36)
			AW	10	5 (50.00)	3 (30.00)	1 (10.00)	0 (0.00)	1 (10.00)
AQ14	I find making up stories easy (R)	Imagination	AC	9	5 (55.56)	0 (0.00)	0 (0.00)	2 (22.22)	2 (22.22)
			AW	10	6 (60.00)	0 (0.00)	1 (10.00)	3 (30.00)	0 (0.00)
AQ15	I find myself drawn more strongly to people than to things (R)	Social skills	AC	10	2 (20.00)	1 (10.00)	2 (20.00)	3 (30.00)	2 (20.00)
			AW	10	3 (30.00)	1 (10.00)	3 (30.00)	3 (30.00)	0 (0.00)

Item	Label	Factor	Expert group	N	Yes, it is relevant as it is (%)	Yes, it is relevant but needs to be improved (%)	No, it is not relevant but can be modified to be relevant (%)	No, it is not relevant to autistic women / to me (%)	No, it is not relevant to autism at all (%)
AQ16	I tend to have very strong interests, which I get upset about if I can't pursue	Attention switching	AC	10	8 (80.00)	2 (20.00)	0 (0.00)	0 (0.00)	0 (0.00)
			AW	10	4 (40.00)	3 (30.00)	2 (20.00)	1 (10.00)	0 (0.00)
AQ17	I enjoy social chitchat (R)	Communication	AC	9	8 (88.89)	0 (0.00)	1 (11.11)	0 (0.00)	0 (0.00)
			AW	10	4 (40.00)	5 (50.00)	0 (0.00)	1 (10.00)	0 (0.00)
AQ18	When I talk, it isn't always easy for others to get a word in edgewise	Communication	AC	9	1 (11.11)	4 (44.44)	3 (33.33)	1 (11.11)	0 (0.00)
			AW	11	5 (45.45)	0 (0.00)	4 (36.36)	2 (18.18)	0 (0.00)
AQ19	I am fascinated by numbers	Attention to detail	AC	9	1 (11.11)	2 (22.22)	2 (22.22)	2 (22.22)	2 (22.22)
			AW	10	3 (30.00)	0 (0.00)	1 (10.00)	5 (50.00)	1 (10.00)
AQ20	When I'm reading a story, I find it difficult to work out the characters' intentions	Imagination	AC	11	6 (54.55)	2 (18.18)	0 (0.00)	3 (27.27)	0 (0.00)
			AW	10	3 (30.00)	1 (10.00)	1 (10.00)	5 (50.00)	0 (0.00)
AQ21	I don't particularly enjoy reading fiction	Imagination	AC	10	1 (10.00)	1 (10.00)	0 (0.00)	5 (50.00)	3 (30.00)
			AW	10	1 (10.00)	0 (0.00)	0 (0.00)	6 (60.00)	3 (30.00)
AQ22	I find it hard to make new friends	Social skills	AC	11	9 (81.82)	1 (9.09)	1 (9.09)	0 (0.00)	0 (0.00)
			AW	10	3 (30.00)	4 (40.00)	2 (20.00)	1 (10.00)	0 (0.00)
AQ23	I notice patterns in things all the time	Attention to detail	AC	10	8 (80.00)	1 (10.00)	0 (0.00)	1 (10.00)	0 (0.00)
			AW	10	5 (50.00)	2 (20.00)	1 (10.00)	2 (20.00)	0 (0.00)
AQ24	I would rather go to the theatre than to a museum (R)	Imagination	AC	11	2 (18.18)	0 (0.00)	2 (18.18)	1 (9.09)	6 (54.55)
			AW	11	4 (36.36)	0 (0.00)	2 (18.18)	3 (27.27)	2 (18.18)
AQ25	It does not upset me if my daily routine is disturbed (R)	Attention switching	AC	11	8 (72.73)	1 (9.09)	1 (9.09)	0 (0.00)	1 (9.09)
			AW	10	4 (40.00)	4 (40.00)	2 (20.00)	0 (0.00)	0 (0.00)

Item	Label	Factor	Expert group	N	Yes, it is relevant as it is (%)	Yes, it is relevant but needs to be improved (%)	No, it is not relevant but can be modified to be relevant (%)	No, it is not relevant to autistic women / to me (%)	No, it is not relevant to autism at all (%)
AQ26	I frequently find that I don't know how to keep a conversation going	Communication	AC	11	9 (81.82)	2 (18.18)	0 (0.00)	0 (0.00)	0 (0.00)
			AW	10	5 (50.00)	2 (20.00)	2 (20.00)	0 (0.00)	1 (10.00)
AQ27	I find it easy to 'read between the lines' when someone is talking to me (R)	Communication	AC	11	8 (72.73)	2 (18.18)	1 (9.09)	0 (0.00)	0 (0.00)
			AW	10	5 (50.00)	4 (40.00)	0 (0.00)	1 (10.00)	0 (0.00)
AQ28	I usually concentrate more on the whole picture, rather than on the small details (R)	Attention to detail	AC	9	6 (66.67)	0 (0.00)	0 (0.00)	2 (22.22)	1 (11.11)
			AW	10	5 (50.00)	2 (20.00)	0 (0.00)	3 (30.00)	0 (0.00)
AQ29	I am not very good at remembering phone numbers (R)	Attention to detail	AC	10	2 (20.00)	1 (10.00)	1 (10.00)	3 (30.00)	3 (30.00)
			AW	10	5 (50.00)	0 (0.00)	1 (10.00)	3 (30.00)	1 (10.00)
AQ30	I don't usually notice small changes in a situation or a person's appearance (R)	Attention to detail	AC	9	7 (77.78)	0 (0.00)	0 (0.00)	1 (11.11)	1 (11.11)
			AW	10	6 (60.00)	1 (10.00)	1 (10.00)	1 (10.00)	1 (10.00)
AQ31	I know how to tell if someone listening to me is getting bored (R)	Communication	AC	10	6 (60.00)	3 (30.00)	1 (10.00)	0 (0.00)	0 (0.00)
			AW	11	6 (54.55)	3 (27.27)	0 (0.00)	2 (18.18)	0 (0.00)
AQ32	I find it easy to do more than one thing at once (R)	Attention switching	AC	10	5 (50.00)	2 (20.00)	1 (10.00)	0 (0.00)	2 (20.00)
			AW	10	7 (70.00)	1 (10.00)	0 (0.00)	1 (10.00)	1 (10.00)
AQ33	When I talk on the phone, I'm not sure when it's my turn to speak	Communication	AC	10	8 (80.00)	0 (0.00)	2 (20.00)	0 (0.00)	0 (0.00)
			AW	10	5 (50.00)	1 (10.00)	1 (10.00)	3 (30.00)	0 (0.00)
AQ34	I enjoy doing things spontaneously (R)	Attention switching	AC	9	7 (77.78)	1 (11.11)	0 (0.00)	0 (0.00)	1 (11.11)
			AW	11	8 (72.73)	2 (18.18)	1 (9.09)	0 (0.00)	0 (0.00)
AQ35	I am often the last to understand the point of a joke	Communication	AC	9	7 (77.78)	1 (11.11)	0 (0.00)	1 (11.11)	0 (0.00)
			AW	10	6 (60.00)	0 (0.00)	2 (20.00)	2 (20.00)	0 (0.00)

Item	Label	Factor	Expert group	N	Yes, it is relevant as it is (%)	Yes, it is relevant but needs to be improved (%)	No, it is not relevant but can be modified to be relevant (%)	No, it is not relevant to autistic women / to me (%)	No, it is not relevant to autism at all (%)
AQ36	I find it easy to work out what someone is thinking or feeling just by looking at their face (R)	Social skills	AC	10	8 (80.00)	0 (0.00)	1 (10.00)	1 (10.00)	0 (0.00)
			AW	10	5 (50.00)	2 (20.00)	1 (10.00)	2 (20.00)	0 (0.00)
AQ37	If there is an interruption, I can switch back to what I was doing very quickly (R)	Attention switching	AC	9	7 (77.78)	1 (11.11)	0 (0.00)	0 (0.00)	1 (11.11)
			AW	10	7 (70.00)	2 (20.00)	0 (0.00)	0 (0.00)	1 (10.00)
AQ38	I am good at social chitchat (R)	Communication	AC	9	7 (77.78)	0 (0.00)	0 (0.00)	1 (11.11)	1 (11.11)
			AW	10	6 (60.00)	3 (30.00)	0 (0.00)	1 (10.00)	0 (0.00)
AQ39	People often tell me that I keep going on and on about the same thing	Communication	AC	9	5 (55.56)	3 (33.33)	1 (11.11)	0 (0.00)	0 (0.00)
			AW	11	7 (63.64)	2 (18.18)	2 (18.18)	0 (0.00)	0 (0.00)
AQ40	When I was young, I used to enjoy playing games involving pretending with other children (R)	Imagination	AC	10	6 (60.00)	1 (10.00)	2 (20.00)	0 (0.00)	1 (10.00)
			AW	10	4 (40.00)	1 (10.00)	0 (0.00)	5 (50.00)	0 (0.00)
AQ41	I like to collect information about categories of things (e.g. types of cars, birds, trains, plants)	Imagination	AC	10	3 (30.00)	5 (50.00)	1 (10.00)	1 (10.00)	0 (0.00)
			AW	10	6 (60.00)	0 (0.00)	3 (30.00)	1 (10.00)	0 (0.00)
AQ42	I find it difficult to imagine what it would be like to be someone else	Imagination	AC	10	4 (40.00)	3 (30.00)	1 (10.00)	1 (10.00)	1 (10.00)
			AW	10	3 (30.00)	1 (10.00)	1 (10.00)	5 (50.00)	0 (0.00)
AQ43	I like to carefully plan any activities I participate in	Attention switching	AC	10	9 (90.00)	1 (10.00)	0 (0.00)	0 (0.00)	0 (0.00)
			AW	10	7 (70.00)	1 (10.00)	0 (0.00)	2 (20.00)	0 (0.00)
AQ44	I enjoy social occasions (R)	Social skills	AC	10	4 (40.00)	2 (20.00)	3 (30.00)	1 (10.00)	0 (0.00)
			AW	11	4 (36.36)	3 (27.27)	1 (9.09)	2 (18.18)	1 (9.09)
AQ45	I find it difficult to work out people's intentions	Social skills	AC	9	5 (55.56)	3 (33.33)	1 (11.11)	0 (0.00)	0 (0.00)
			AW	10	6 (60.00)	2 (20.00)	2 (20.00)	0 (0.00)	0 (0.00)

Item	Label	Factor	Expert group	N	Yes, it is relevant as it is (%)	Yes, it is relevant but needs to be improved (%)	No, it is not relevant but can be modified to be relevant (%)	No, it is not relevant to autistic women / to me (%)	No, it is not relevant to autism at all (%)
AQ46	New situations make me anxious	Attention switching	AC	10	9 (90.00)	1 (10.00)	0 (0.00)	0 (0.00)	0 (0.00)
			AW	10	8 (80.00)	2 (20.00)	0 (0.00)	0 (0.00)	0 (0.00)
AQ47	I enjoy meeting new people (R)	Social skills	AC	10	6 (60.00)	0 (0.00)	0 (0.00)	2 (20.00)	2 (20.00)
			AW	10	4 (40.00)	2 (20.00)	1 (10.00)	2 (20.00)	1 (10.00)
AQ48	I am a good diplomat (R)	Social skills	AC	10	1 (10.00)	4 (40.00)	0 (0.00)	2 (20.00)	3 (30.00)
			AW	10	3 (30.00)	2 (20.00)	3 (30.00)	2 (20.00)	0 (0.00)
AQ49	I am not very good at remembering people's date of birth (R)	Attention to detail	AC	9	1 (11.11)	2 (22.22)	0 (0.00)	3 (33.33)	3 (33.33)
			AW	10	2 (20.00)	1 (10.00)	1 (10.00)	2 (20.00)	4 (40.00)
AQ50	I find it very easy to play games with children that involve pretending (R)	Imagination	AC	11	4 (36.36)	5 (45.45)	1 (9.09)	0 (0.00)	1 (9.09)
			AW	10	4 (40.00)	1 (10.00)	0 (0.00)	5 (50.00)	0 (0.00)

Note. AQ: Autism Spectrum Quotient, AC: academics and clinicians, AW: autistic women. (R) signifies a reverse scored statement.

Table A5

Frequencies of relevant and non-relevant response options, I-CVI, p_c, and k^* per item for AQ across academics and clinicians and autistic women

Item	Label	Factor	Expert group	N	Relevant (%)	Not Relevant (%)	I-CVI	pc	k*	Interpretation	Decision
AQ01	I prefer to do things with others, rather than on my own (R)	Social skills	AC	9	5 (55.6)	4 (44.4)	0.556	0.246	0.410	Fair	Discarded
			AW	10	7 (70.0)	3 (30.0)	0.700	0.117	0.660	Good	
AQ02	I prefer to do things the same way over and over again	Attention switching	AC	10	9 (90.0)	1 (10.0)	0.900	0.010	0.899	Excellent	**Included**
			AW	10	10 (100.0)	0 (0.0)	1.000	0.001	1.000	Excellent	

Item	Label	Factor	Expert group	N	Relevant (%)	Not Relevant (%)	I-CVI	pc	k*	Interpretation	Decision
AQ03	If I try to imagine something, I find it very easy to create a picture in my mind (R)	Imagination	AC	11	5 (45.5)	6 (54.5)	0.455	0.226	0.296	Poor	Discarded
			AW	10	5 (50.0)	5 (50.0)	0.500	0.246	0.337	Poor	
AQ04	I frequently get so strongly absorbed in one thing that I lose sight of other things	Attention switching	AC	11	11 (100.0)	0 (0.0)	1.000	0.000	1.000	Excellent	**Included**
			AW	10	8 (80.0)	2 (20.0)	0.800	0.044	0.791	Excellent	
AQ05	I often notice small sounds when others do not	Attention to detail	AC	10	10 (100.0)	0 (0.0)	1.000	0.001	1.000	Excellent	**Included**
			AW	10	9 (90.0)	1 (10.0)	0.900	0.010	0.899	Excellent	
AQ06	I usually notice car number plates or similar strings of information	Attention to detail	AC	9	5 (55.6)	4 (44.4)	0.556	0.246	0.410	Fair	Discarded
			AW	11	6 (54.5)	5 (45.5)	0.545	0.226	0.413	Fair	
AQ07	Other people frequently tell me that what I've said is impolite, even though I think it is polite	Communication	AC	10	10 (100.0)	0 (0.0)	1.000	0.001	1.000	Excellent	**Included**
			AW	10	8 (80.0)	2 (20.0)	0.800	0.044	0.791	Excellent	
AQ08	When I'm reading a story, I can easily imagine what the characters might look like (R)	Imagination	AC	10	3 (30.0)	7 (70.0)	0.300	0.117	0.207	Poor	Discarded
			AW	10	5 (50.0)	5 (50.0)	0.500	0.246	0.337	Poor	
AQ09	I am fascinated by dates	Attention to detail	AC	10	3 (30.0)	7 (70.0)	0.300	0.117	0.207	Poor	Discarded
			AW	10	1 (10.0)	9 (90.0)	0.100	0.010	0.091	Poor	
AQ10	In a social group, I can easily keep track of several different people's conversations (R)	Attention switching	AC	10	9 (90.0)	1 (10.0)	0.900	0.010	0.899	Excellent	**Included**
			AW	10	10 (100.0)	0 (0.0)	1.000	0.001	1.000	Excellent	
AQ11	I find social situations easy (R)	Social skills	AC	10	9 (90.0)	1 (10.0)	0.900	0.010	0.899	Excellent	**Included**
			AW	10	9 (90.0)	1 (10.0)	0.900	0.010	0.899	Excellent	
AQ12	I tend to notice details that others do not	Attention to detail	AC	10	7 (70.0)	3 (30.0)	0.700	0.117	0.660	Good	Discarded
			AW	10	9 (90.0)	1 (10.0)	0.900	0.010	0.899	Excellent	

Item	Label	Factor	Expert group	N	Relevant (%)	Not Relevant (%)	I-CVI	pc	k*	Interpretation	Decision
AQ13	I would rather go to a library than to a party	Social skills	AC	11	5 (45.5)	6 (54.5)	0.455	0.226	0.296	Poor	Discarded
			AW	10	8 (80.0)	2 (20.0)	0.800	0.044	0.791	Excellent	
AQ14	I find making up stories easy (R)	Imagination	AC	9	5 (55.6)	4 (44.4)	0.556	0.246	0.410	Fair	Discarded
			AW	10	6 (60.0)	4 (40.0)	0.600	0.205	0.497	Fair	
AQ15	I find myself drawn more strongly to people than to things (R)	Social skills	AC	10	3 (30.0)	7 (70.0)	0.300	0.117	0.207	Poor	Discarded
			AW	10	4 (40.0)	6 (60.0)	0.400	0.205	0.245	Poor	
AQ16	I tend to have very strong interests, which I get upset about if I can't pursue	Attention switching	AC	10	10 (100.0)	0 (0.0)	1.000	0.001	1.000	Excellent	Discarded
			AW	10	7 (70.0)	3 (30.0)	0.700	0.117	0.660	Good	
AQ17	I enjoy social chitchat (R)	Communication	AC	9	8 (88.9)	1 (11.1)	0.889	0.018	0.887	Excellent	**Included**
			AW	10	9 (90.0)	1 (10.0)	0.900	0.010	0.899	Excellent	
AQ18	When I talk, it isn't always easy for others to get a word in edgewise	Communication	AC	9	5 (55.6)	4 (44.4)	0.556	0.246	0.410	Fair	Discarded
			AW	11	5 (45.5)	6 (54.5)	0.455	0.226	0.296	Poor	
AQ19	I am fascinated by numbers	Attention to detail	AC	9	3 (33.3)	6 (66.7)	0.333	0.164	0.202	Poor	Discarded
			AW	10	3 (30.0)	7 (70.0)	0.300	0.117	0.207	Poor	
AQ20	When I'm reading a story, I find it difficult to work out the characters' intentions	Imagination	AC	11	8 (72.7)	3 (27.3)	0.727	0.081	0.703	Good	Discarded
			AW	10	4 (40.0)	6 (60.0)	0.400	0.205	0.245	Poor	
AQ21	I don't particularly enjoy reading fiction	Imagination	AC	10	2 (20.0)	8 (80.0)	0.200	0.044	0.163	Poor	Discarded
			AW	10	1 (10.0)	9 (90.0)	0.100	0.010	0.091	Poor	
AQ22	I find it hard to make new friends	Social skills	AC	11	10 (90.9)	1 (9.1)	0.909	0.005	0.909	Excellent	Discarded
			AW	10	7 (70.0)	3 (30.0)	0.700	0.117	0.660	Good	

Item	Label	Factor	Expert group	N	Relevant (%)	Not Relevant (%)	I-CVI	pc	k*	Interpretation	Decision
AQ23	I notice patterns in things all the time	Attention to detail	AC	10	9 (90.0)	1 (10.0)	0.900	0.010	0.899	Excellent	Discarded
			AW	10	7 (70.0)	3 (30.0)	0.700	0.117	0.660	Good	
AQ24	I would rather go to the theatre than to a museum (R)	Imagination	AC	11	2 (18.2)	9 (81.8)	0.182	0.027	0.159	Poor	Discarded
			AW	11	4 (36.4)	7 (63.6)	0.364	0.161	0.241	Poor	
AQ25	It does not upset me if my daily routine is disturbed (R)	Attention switching	AC	11	9 (81.8)	2 (18.2)	0.818	0.027	0.813	Excellent	**Included**
			AW	10	8 (80.0)	2 (20.0)	0.800	0.044	0.791	Excellent	
AQ26	I frequently find that I don't know how to keep a conversation going	Communication	AC	11	11 (100.0)	0 (0.0)	1.000	0.000	1.000	Excellent	Discarded
			AW	10	7 (70.0)	3 (30.0)	0.700	0.117	0.660	Good	
AQ27	I find it easy to 'read between the lines' when someone is talking to me (R)	Communication	AC	11	10 (90.9)	1 (9.1)	0.909	0.005	0.909	Excellent	**Included**
			AW	10	9 (90.0)	1 (10.0)	0.900	0.010	0.899	Excellent	
AQ28	I usually concentrate more on the whole picture, rather than on the small details (R)	Attention to detail	AC	9	6 (66.7)	3 (33.3)	0.667	0.164	0.601	Good	Discarded
			AW	10	7 (70.0)	3 (30.0)	0.700	0.117	0.660	Good	
AQ29	I am not very good at remembering phone numbers (R)	Attention to detail	AC	10	3 (30.0)	7 (70.0)	0.300	0.117	0.207	Poor	Discarded
			AW	10	5 (50.0)	5 (50.0)	0.500	0.246	0.337	Poor	
AQ30	I don't usually notice small changes in a situation or a person's appearance (R)	Attention to detail	AC	9	7 (77.8)	2 (22.2)	0.778	0.070	0.761	Excellent	Discarded
			AW	10	7 (70.0)	3 (30.0)	0.700	0.117	0.660	Good	
AQ31	I know how to tell if someone listening to me is getting bored (R)	Communication	AC	10	9 (90.0)	1 (10.0)	0.900	0.010	0.899	Excellent	**Included**
			AW	11	9 (81.8)	2 (18.2)	0.818	0.027	0.813	Excellent	
AQ32	I find it easy to do more than one thing at once (R)	Attention switching	AC	10	7 (70.0)	3 (30.0)	0.700	0.117	0.660	Good	Discarded
			AW	10	8 (80.0)	2 (20.0)	0.800	0.044	0.791	Excellent	

Item	Label	Factor	Expert group	N	Relevant (%)	Not Relevant (%)	I-CVI	pc	k*	Interpretation	Decision
AQ33	When I talk on the phone, I'm not sure when it's my turn to speak	Communication	AC	10	8 (80.0)	2 (20.0)	0.800	0.044	0.791	Excellent	Discarded
			AW	10	6 (60.0)	4 (40.0)	0.600	0.205	0.497	Fair	
AQ34	I enjoy doing things spontaneously (R)	Attention switching	AC	9	8 (88.9)	1 (11.1)	0.889	0.018	0.887	Excellent	**Included**
			AW	11	10 (90.9)	1 (9.1)	0.909	0.005	0.909	Excellent	
AQ35	I am often the last to understand the point of a joke	Communication	AC	9	8 (88.9)	1 (11.1)	0.889	0.018	0.887	Excellent	Discarded
			AW	10	6 (60.0)	4 (40.0)	0.600	0.205	0.497	Fair	
AQ36	I find it easy to work out what someone is thinking or feeling just by looking at their face (R)	Social skills	AC	10	8 (80.0)	2 (20.0)	0.800	0.044	0.791	Excellent	Discarded
			AW	10	7 (70.0)	3 (30.0)	0.700	0.117	0.660	Good	
AQ37	If there is an interruption, I can switch back to what I was doing very quickly (R)	Attention switching	AC	9	8 (88.9)	1 (11.1)	0.889	0.018	0.887	Excellent	**Included**
			AW	10	9 (90.0)	1 (10.0)	0.900	0.010	0.899	Excellent	
AQ38	I am good at social chitchat (R)	Communication	AC	9	7 (77.8)	2 (22.2)	0.778	0.070	0.761	Excellent	**Included**
			AW	10	9 (90.0)	1 (10.0)	0.900	0.010	0.899	Excellent	
AQ39	People often tell me that I keep going on and on about the same thing	Communication	AC	9	8 (88.9)	1 (11.1)	0.889	0.018	0.887	Excellent	**Included**
			AW	11	9 (81.8)	2 (18.2)	0.818	0.027	0.813	Excellent	
AQ40	When I was young, I used to enjoy playing games involving pretending with other children (R)	Imagination	AC	10	7 (70.0)	3 (30.0)	0.700	0.117	0.660	Good	Discarded
			AW	10	5 (50.0)	5 (50.0)	0.500	0.246	0.337	Poor	
AQ41	I like to collect information about categories of things (e.g. types of cars, birds, trains, plants)	Imagination	AC	10	8 (80.0)	2 (20.0)	0.800	0.044	0.791	Excellent	Discarded
			AW	10	6 (60.0)	4 (40.0)	0.600	0.205	0.497	Fair	
AQ42	I find it difficult to imagine what it would be like to be someone else	Imagination	AC	10	7 (70.0)	3 (30.0)	0.700	0.117	0.660	Good	Discarded
			AW	10	4 (40.0)	6 (60.0)	0.400	0.205	0.245	Poor	

Item	Label	Factor	Expert group	N	Relevant (%)	Not Relevant (%)	I-CVI	pc	k*	Interpretation	Decision
AQ43	I like to carefully plan any activities I participate in	Attention switching	AC	10	10 (100.0)	0 (0.0)	1.000	0.001	1.000	Excellent	**Included**
			AW	10	8 (80.0)	2 (20.0)	0.800	0.044	0.791	Excellent	
AQ44	I enjoy social occasions (R)	Social skills	AC	10	6 (60.0)	4 (40.0)	0.600	0.205	0.497	Fair	Discarded
			AW	11	7 (63.6)	4 (36.4)	0.636	0.161	0.567	Fair	
AQ45	I find it difficult to work out people's intentions	Social skills	AC	9	8 (88.9)	1 (11.1)	0.889	0.018	0.887	Excellent	**Included**
			AW	10	8 (80.0)	2 (20.0)	0.800	0.044	0.791	Excellent	
AQ46	New situations make me anxious	Attention switching	AC	10	10 (100.0)	0 (0.0)	1.000	0.001	1.000	Excellent	**Included**
			AW	10	10 (100.0)	0 (0.0)	1.000	0.001	1.000	Excellent	
AQ47	I enjoy meeting new people (R)	Social skills	AC	10	6 (60.0)	4 (40.0)	0.600	0.205	0.497	Fair	Discarded
			AW	10	6 (60.0)	4 (40.0)	0.600	0.205	0.497	Fair	
AQ48	I am a good diplomat (R)	Social skills	AC	10	5 (50.0)	5 (50.0)	0.500	0.246	0.337	Poor	Discarded
			AW	10	5 (50.0)	5 (50.0)	0.500	0.246	0.337	Poor	
AQ49	I am not very good at remembering people's date of birth (R)	Attention to detail	AC	9	3 (33.3)	6 (66.7)	0.333	0.164	0.202	Poor	Discarded
			AW	10	3 (30.0)	7 (70.0)	0.300	0.117	0.207	Poor	
AQ50	I find it very easy to play games with children that involve pretending (R)	Imagination	AC	11	9 (81.8)	2 (18.2)	0.818	0.027	0.813	Excellent	Discarded
			AW	10	5 (50.0)	5 (50.0)	0.500	0.246	0.337	Poor	

Note. AQ: Autism Spectrum Quotient, AC: academics and clinicians, AW: autistic women, I-CVI: item-level content validity index, pc: probability of a chance occurrence, k*: modified kappa. Agreement levels are indicated by modified kappa (k*) values: below 0.40 = poor, 0.40–0.59 = fair, 0.60–0.74 = good, and above 0.74 = excellent. Relevant items are those rated by experts as 1 ('Yes, it is relevant as is') or 2 ('Yes, it is relevant but needs to be improved'). Non-relevant items are those rated as 3 ('No, it is not relevant but it can be modified to be relevant'), 4 ('No, it is not relevant to autistic women'), or 5 ('No, it is not relevant to autism at all'). (R) signifies a reverse scored statement.

Table A6

Frequencies of response options for BAPQ relevance evaluations across academics, clinicians, and autistic women

Item	Label	Factor	Expert group	N	Yes, it is relevant as it is (%)	Yes, it is relevant but needs to be improved (%)	No, it is not relevant but can be modified to be relevant (%)	No, it is not relevant to autistic women / to me (%)	No, it is not relevant to autism at all (%)
BAPQ01	I like being around other people (R)	Aloof	AC	10	6 (60.00)	2 (20.00)	1 (10.00)	1 (10.00)	0 (0.00)
			AW	10	3 (30.00)	7 (70.00)	0 (0.00)	0 (0.00)	0 (0.00)
BAPQ02	I find it hard to get my words out smoothly	Pragmatic Language	AC	10	4 (40.00)	3 (30.00)	1 (10.00)	1 (10.00)	1 (10.00)
			AW	11	7 (63.64)	3 (27.27)	0 (0.00)	1 (9.09)	0 (0.00)
BAPQ03	I am comfortable with unexpected changes in plans (R)	Rigid	AC	11	7 (63.64)	2 (18.18)	0 (0.00)	2 (18.18)	0 (0.00)
			AW	11	10 (90.91)	1 (9.09)	0 (0.00)	0 (0.00)	0 (0.00)
BAPQ04	It's hard for me to avoid getting sidetracked in conversation	Pragmatic Language	AC	11	4 (36.36)	5 (45.45)	0 (0.00)	1 (9.09)	1 (9.09)
			AW	10	3 (30.00)	6 (60.00)	0 (0.00)	0 (0.00)	1 (10.00)
BAPQ05	I would rather talk to people to get information than to socialize	Aloof	AC	11	6 (54.55)	3 (27.27)	1 (9.09)	1 (9.09)	0 (0.00)
			AW	11	6 (54.55)	0 (0.00)	2 (18.18)	2 (18.18)	1 (9.09)
BAPQ06	People have to talk me into trying something new	Rigid	AC	11	6 (54.55)	2 (18.18)	1 (9.09)	1 (9.09)	1 (9.09)
			AW	11	6 (54.55)	4 (36.36)	0 (0.00)	1 (9.09)	0 (0.00)
BAPQ07	I am "in-tune" with the other person during conversation (R)	Pragmatic Language	AC	11	2 (18.18)	6 (54.55)	0 (0.00)	3 (27.27)	0 (0.00)
			AW	10	5 (50.00)	2 (20.00)	0 (0.00)	3 (30.00)	0 (0.00)
BAPQ08	I have to warm myself up to the idea of visiting an unfamiliar place	Rigid	AC	11	5 (45.45)	5 (45.45)	0 (0.00)	1 (9.09)	0 (0.00)
			AW	11	7 (63.64)	4 (36.36)	0 (0.00)	0 (0.00)	0 (0.00)
BAPQ09	I enjoy being in social situations (R)	Aloof	AC	10	1 (10.00)	6 (60.00)	1 (10.00)	2 (20.00)	0 (0.00)
			AW	11	7 (63.64)	4 (36.36)	0 (0.00)	0 (0.00)	0 (0.00)
BAPQ10			AC	10	3 (30.00)	5 (50.00)	0 (0.00)	2 (20.00)	0 (0.00)

Item	Label	Factor	Expert group	N	Yes, it is relevant as it is (%)	Yes, it is relevant but needs to be improved (%)	No, it is not relevant but can be modified to be relevant (%)	No, it is not relevant to autistic women / to me (%)	No, it is not relevant to autism at all (%)
	My voice has a flat or monotone sound to it	Pragmatic Language	AW	11	3 (27.27)	2 (18.18)	0 (0.00)	6 (54.55)	0 (0.00)
BAPQ11	I feel disconnected or ''out of sync'' in conversations with others	Pragmatic Language	AC	10	7 (70.00)	2 (20.00)	0 (0.00)	1 (10.00)	0 (0.00)
			AW	11	9 (81.82)	2 (18.18)	0 (0.00)	0 (0.00)	0 (0.00)
BAPQ12	People find it easy to approach me (R)	Aloof	AC	11	1 (9.09)	4 (36.36)	2 (18.18)	4 (36.36)	0 (0.00)
			AW	10	5 (50.00)	1 (10.00)	0 (0.00)	3 (30.00)	1 (10.00)
BAPQ13	I feel a strong need for sameness from day to day	Rigid	AC	10	6 (60.00)	1 (10.00)	0 (0.00)	3 (30.00)	0 (0.00)
			AW	11	7 (63.64)	4 (36.36)	0 (0.00)	0 (0.00)	0 (0.00)
BAPQ14	People ask me to repeat things I've said because they don't understand	Pragmatic Language	AC	11	5 (45.45)	1 (9.09)	2 (18.18)	2 (18.18)	1 (9.09)
			AW	11	5 (45.45)	4 (36.36)	0 (0.00)	1 (9.09)	1 (9.09)
BAPQ15	I am flexible about how things should be done (R)	Rigid	AC	11	6 (54.55)	2 (18.18)	0 (0.00)	2 (18.18)	1 (9.09)
			AW	10	3 (30.00)	6 (60.00)	0 (0.00)	1 (10.00)	0 (0.00)
BAPQ16	I look forward to situations where I can meet new people (R)	Aloof	AC	10	5 (50.00)	3 (30.00)	0 (0.00)	2 (20.00)	0 (0.00)
			AW	10	8 (80.00)	0 (0.00)	0 (0.00)	1 (10.00)	1 (10.00)
BAPQ17	I have been told that I talk too much about certain topics	Pragmatic Language	AC	10	7 (70.00)	2 (20.00)	0 (0.00)	1 (10.00)	0 (0.00)
			AW	11	4 (36.36)	0 (0.00)	0 (0.00)	6 (54.55)	1 (9.09)
BAPQ18	When I make conversation it is just to be polite	Aloof	AC	10	3 (30.00)	4 (40.00)	0 (0.00)	2 (20.00)	1 (10.00)
			AW	10	5 (50.00)	2 (20.00)	1 (10.00)	1 (10.00)	1 (10.00)
BAPQ19	I look forward to trying new things (R)	Rigid	AC	10	4 (40.00)	3 (30.00)	1 (10.00)	1 (10.00)	1 (10.00)
			AW	10	4 (40.00)	5 (50.00)	1 (10.00)	0 (0.00)	0 (0.00)

Item	Label	Factor	Expert group	N	Yes, it is relevant as it is (%)	Yes, it is relevant but needs to be improved (%)	No, it is not relevant but can be modified to be relevant (%)	No, it is not relevant to autistic women / to me (%)	No, it is not relevant to autism at all (%)
BAPQ20	I speak too loudly or softly	Pragmatic Language	AC	9	3 (33.33)	5 (55.56)	0 (0.00)	1 (11.11)	0 (0.00)
			AW	10	8 (80.00)	2 (20.00)	0 (0.00)	0 (0.00)	0 (0.00)
BAPQ21	I can tell when someone is not interested in what I am saying (R)	Pragmatic Language	AC	10	3 (30.00)	5 (50.00)	0 (0.00)	2 (20.00)	0 (0.00)
			AW	10	3 (30.00)	2 (20.00)	0 (0.00)	5 (50.00)	0 (0.00)
BAPQ22	I have a hard time dealing with changes in my routine	Rigid	AC	10	6 (60.00)	3 (30.00)	0 (0.00)	1 (10.00)	0 (0.00)
			AW	11	11 (100.00)	0 (0.00)	0 (0.00)	0 (0.00)	0 (0.00)
BAPQ23	I am good at making small talk (R)	Aloof	AC	11	4 (36.36)	4 (36.36)	0 (0.00)	1 (9.09)	2 (18.18)
			AW	10	8 (80.00)	0 (0.00)	0 (0.00)	2 (20.00)	0 (0.00)
BAPQ24	I act very set in my ways	Rigid	AC	9	2 (22.22)	4 (44.44)	0 (0.00)	3 (33.33)	0 (0.00)
			AW	10	6 (60.00)	3 (30.00)	1 (10.00)	0 (0.00)	0 (0.00)
BAPQ25	I feel like I am really connecting with other people (R)	Aloof	AC	10	2 (20.00)	4 (40.00)	0 (0.00)	1 (10.00)	3 (30.00)
			AW	11	6 (54.55)	3 (27.27)	1 (9.09)	1 (9.09)	0 (0.00)
BAPQ26	People get frustrated by my unwillingness to bend	Rigid	AC	11	3 (27.27)	5 (45.45)	1 (9.09)	2 (18.18)	0 (0.00)
			AW	10	2 (20.00)	5 (50.00)	0 (0.00)	3 (30.00)	0 (0.00)
BAPQ27	Conversation bores me	Aloof	AC	11	2 (18.18)	4 (36.36)	3 (27.27)	2 (18.18)	0 (0.00)
			AW	11	3 (27.27)	4 (36.36)	2 (18.18)	1 (9.09)	1 (9.09)
BAPQ28	I am warm and friendly in my interactions with others (R)	Aloof	AC	11	2 (18.18)	6 (54.55)	1 (9.09)	2 (18.18)	0 (0.00)
			AW	10	2 (20.00)	2 (20.00)	1 (10.00)	5 (50.00)	0 (0.00)
BAPQ29	I leave long pauses in conversation	Pragmatic Language	AC	9	2 (22.22)	3 (33.33)	2 (22.22)	2 (22.22)	0 (0.00)
			AW	10	5 (50.00)	2 (20.00)	1 (10.00)	2 (20.00)	0 (0.00)

Item	Label	Factor	Expert group	N	Yes, it is relevant as it is (%)	Yes, it is relevant but needs to be improved (%)	No, it is not relevant but can be modified to be relevant (%)	No, it is not relevant to autistic women / to me (%)	No, it is not relevant to autism at all (%)
BAPQ30	I alter my daily routine by trying something different (R)	Rigid	AC	10	3 (30.00)	4 (40.00)	0 (0.00)	3 (30.00)	0 (0.00)
			AW	10	6 (60.00)	3 (30.00)	1 (10.00)	0 (0.00)	0 (0.00)
BAPQ31	I prefer to be alone rather than with others	Aloof	AC	9	2 (22.22)	4 (44.44)	1 (11.11)	2 (22.22)	0 (0.00)
			AW	11	6 (54.55)	4 (36.36)	0 (0.00)	1 (9.09)	0 (0.00)
BAPQ32	I lose track of my original point when talking to people	Pragmatic Language	AC	11	6 (54.55)	1 (9.09)	1 (9.09)	2 (18.18)	1 (9.09)
			AW	10	5 (50.00)	2 (20.00)	0 (0.00)	1 (10.00)	2 (20.00)
BAPQ33	I like to closely follow a routine while working	Rigid	AC	11	7 (63.64)	2 (18.18)	0 (0.00)	2 (18.18)	0 (0.00)
			AW	10	5 (50.00)	4 (40.00)	0 (0.00)	1 (10.00)	0 (0.00)
BAPQ34	I can tell when it is time to change topics in conversation (R)	Pragmatic Language	AC	11	6 (54.55)	3 (27.27)	0 (0.00)	1 (9.09)	1 (9.09)
			AW	10	5 (50.00)	3 (30.00)	0 (0.00)	2 (20.00)	0 (0.00)
BAPQ35	I keep doing things the way I know, even if another way might be better	Rigid	AC	11	6 (54.55)	2 (18.18)	1 (9.09)	1 (9.09)	1 (9.09)
			AW	10	4 (40.00)	4 (40.00)	0 (0.00)	1 (10.00)	1 (10.00)
BAPQ36	I enjoy chatting with people (R)	Aloof	AC	11	4 (36.36)	4 (36.36)	1 (9.09)	2 (18.18)	0 (0.00)
			AW	11	5 (45.45)	3 (27.27)	1 (9.09)	2 (18.18)	0 (0.00)

Note. BAPQ: Broad Autism Phenotype Questionnaire, AC: academics and clinicians, AW: autistic women. (R) signifies a reverse scored statement.

Table A7

Frequencies of relevant and non-relevant response options, I-CVI, p_c, and k^* per item for BAPQ across academics and clinicians and autistic women

Item	Label	Factor	Expert group	N	Relevant (%)	Not Relevant (%)	I-CVI	pc	k*	Interpretation	Decision
BAPQ01		Aloof	AC	10	8 (80.0)	2 (20.0)	0.800	0.044	0.791	Excellent	**Included**

Item	Label	Factor	Expert group	N	Relevant (%)	Not Relevant (%)	I-CVI	pc	k*	Interpretation	Decision
	I like being around other people (R)		AW	10	10 (100.0)	0 (0.0)	1.000	0.001	1.000	Excellent	
BAPQ02	I find it hard to get my words out smoothly	Pragmatic Language	AC	10	7 (70.0)	3 (30.0)	0.700	0.117	0.660	Good	Discarded
			AW	11	10 (90.9)	1 (9.1)	0.909	0.005	0.909	Excellent	
BAPQ03	I am comfortable with unexpected changes in plans (R)	Rigid	AC	11	9 (81.8)	2 (18.2)	0.818	0.027	0.813	Excellent	**Included**
			AW	11	11 (100.0)	0 (0.0)	1.000	0.000	1.000	Excellent	
BAPQ04	It's hard for me to avoid getting sidetracked in conversation	Pragmatic Language	AC	11	9 (81.8)	2 (18.2)	0.818	0.027	0.813	Excellent	**Included**
			AW	10	9 (90.0)	1 (10.0)	0.900	0.010	0.899	Excellent	
BAPQ05	I would rather talk to people to get information than to socialize	Aloof	AC	11	9 (81.8)	2 (18.2)	0.818	0.027	0.813	Excellent	Discarded
			AW	11	6 (54.5)	5 (45.5)	0.545	0.226	0.413	Fair	
BAPQ06	People have to talk me into trying something new	Rigid	AC	11	8 (72.7)	3 (27.3)	0.727	0.081	0.703	Good	Discarded
			AW	11	10 (90.9)	1 (9.1)	0.909	0.005	0.909	Excellent	
BAPQ07	I am "in-tune" with the other person during conversation (R)	Pragmatic Language	AC	11	8 (72.7)	3 (27.3)	0.727	0.081	0.703	Good	Discarded
			AW	10	7 (70.0)	3 (30.0)	0.700	0.117	0.660	Good	
BAPQ08	I have to warm myself up to the idea of visiting an unfamiliar place	Rigid	AC	11	10 (90.9)	1 (9.1)	0.909	0.005	0.909	Excellent	**Included**
			AW	11	11 (100.0)	0 (0.0)	1.000	0.000	1.000	Excellent	
BAPQ09	I enjoy being in social situations (R)	Aloof	AC	10	7 (70.0)	3 (30.0)	0.700	0.117	0.660	Good	Discarded
			AW	11	11 (100.0)	0 (0.0)	1.000	0.000	1.000	Excellent	
BAPQ10	My voice has a flat or monotone sound to it	Pragmatic Language	AC	10	8 (80.0)	2 (20.0)	0.800	0.044	0.791	Excellent	Discarded
			AW	11	5 (45.5)	6 (54.5)	0.455	0.226	0.296	Poor	
BAPQ11	I feel disconnected or "out of sync" in		AC	10	9 (90.0)	1 (10.0)	0.900	0.010	0.899	Excellent	**Included**

Item	Label	Factor	Expert group	N	Relevant (%)	Not Relevant (%)	I-CVI	pc	k*	Interpretation	Decision
	conversations with others	Pragmatic Language	AW	11	11 (100.0)	0 (0.0)	1.000	0.000	1.000	Excellent	
BAPQ12	People find it easy to approach me (R)	Aloof	AC	11	5 (45.5)	6 (54.5)	0.455	0.226	0.296	Poor	Discarded
			AW	10	6 (60.0)	4 (40.0)	0.600	0.205	0.497	Fair	
BAPQ13	I feel a strong need for sameness from day to day	Rigid	AC	10	7 (70.0)	3 (30.0)	0.700	0.117	0.660	Good	Discarded
			AW	11	11 (100.0)	0 (0.0)	1.000	0.000	1.000	Excellent	
BAPQ14	People ask me to repeat things I've said because they don't understand	Pragmatic Language	AC	11	6 (54.5)	5 (45.5)	0.545	0.226	0.413	Fair	Discarded
			AW	11	9 (81.8)	2 (18.2)	0.818	0.027	0.813	Excellent	
BAPQ15	I am flexible about how things should be done (R)	Rigid	AC	11	8 (72.7)	3 (27.3)	0.727	0.081	0.703	Good	Discarded
			AW	10	9 (90.0)	1 (10.0)	0.900	0.010	0.899	Excellent	
BAPQ16	I look forward to situations where I can meet new people (R)	Aloof	AC	10	8 (80.0)	2 (20.0)	0.800	0.044	0.791	Excellent	**Included**
			AW	10	8 (80.0)	2 (20.0)	0.800	0.044	0.791	Excellent	
BAPQ17	I have been told that I talk too much about certain topics	Pragmatic Language	AC	10	9 (90.0)	1 (10.0)	0.900	0.010	0.899	Excellent	Discarded
			AW	11	4 (36.4)	7 (63.6)	0.364	0.161	0.241	Poor	
BAPQ18	When I make conversation it is just to be polite	Aloof	AC	10	7 (70.0)	3 (30.0)	0.700	0.117	0.660	Good	Discarded
			AW	10	7 (70.0)	3 (30.0)	0.700	0.117	0.660	Good	
BAPQ19	I look forward to trying new things (R)	Rigid	AC	10	7 (70.0)	3 (30.0)	0.700	0.117	0.660	Good	Discarded
			AW	10	9 (90.0)	1 (10.0)	0.900	0.010	0.899	Excellent	
BAPQ20	I speak too loudly or softly	Pragmatic Language	AC	9	8 (88.9)	1 (11.1)	0.889	0.018	0.887	Excellent	**Included**
			AW	10	10 (100.0)	0 (0.0)	1.000	0.001	1.000	Excellent	
BAPQ21	I can tell when someone is not		AC	10	8 (80.0)	2 (20.0)	0.800	0.044	0.791	Excellent	Discarded

Item	Label	Factor	Expert group	N	Relevant (%)	Not Relevant (%)	I-CVI	pc	k*	Interpretation	Decision
	interested in what I am saying (R)	Pragmatic Language	AW	10	5 (50.0)	5 (50.0)	0.500	0.246	0.337	Poor	
BAPQ22	I have a hard time dealing with changes in my routine	Rigid	AC	10	9 (90.0)	1 (10.0)	0.900	0.010	0.899	Excellent	**Included**
			AW	11	11 (100.0)	0 (0.0)	1.000	0.000	1.000	Excellent	
BAPQ23	I am good at making small talk (R)	Aloof	AC	11	8 (72.7)	3 (27.3)	0.727	0.081	0.703	Good	Discarded
			AW	10	8 (80.0)	2 (20.0)	0.800	0.044	0.791	Excellent	
BAPQ24	I act very set in my ways	Rigid	AC	9	6 (66.7)	3 (33.3)	0.667	0.164	0.601	Good	Discarded
			AW	10	9 (90.0)	1 (10.0)	0.900	0.010	0.899	Excellent	
BAPQ25	I feel like I am really connecting with other people (R)	Aloof	AC	10	6 (60.0)	4 (40.0)	0.600	0.205	0.497	Fair	Discarded
			AW	11	9 (81.8)	2 (18.2)	0.818	0.027	0.813	Excellent	
BAPQ26	People get frustrated by my unwillingness to bend	Rigid	AC	11	8 (72.7)	3 (27.3)	0.727	0.081	0.703	Good	Discarded
			AW	10	7 (70.0)	3 (30.0)	0.700	0.117	0.660	Good	
BAPQ27	Conversation bores me	Aloof	AC	11	6 (54.5)	5 (45.5)	0.545	0.226	0.413	Fair	Discarded
			AW	11	7 (63.6)	4 (36.4)	0.636	0.161	0.567	Fair	
BAPQ28	I am warm and friendly in my interactions with others (R)	Aloof	AC	11	8 (72.7)	3 (27.3)	0.727	0.081	0.703	Good	Discarded
			AW	10	4 (40.0)	6 (60.0)	0.400	0.205	0.245	Poor	
BAPQ29	I leave long pauses in conversation	Pragmatic Language	AC	9	5 (55.6)	4 (44.4)	0.556	0.246	0.410	Fair	Discarded
			AW	10	7 (70.0)	3 (30.0)	0.700	0.117	0.660	Good	
BAPQ30	I alter my daily routine by trying something different (R)	Rigid	AC	10	7 (70.0)	3 (30.0)	0.700	0.117	0.660	Good	Discarded
			AW	10	9 (90.0)	1 (10.0)	0.900	0.010	0.899	Excellent	
BAPQ31		Aloof	AC	9	6 (66.7)	3 (33.3)	0.667	0.164	0.601	Good	Discarded

Item	Label	Factor	Expert group	N	Relevant (%)	Not Relevant (%)	I-CVI	pc	k*	Interpretation	Decision
	I prefer to be alone rather than with others		AW	11	10 (90.9)	1 (9.1)	0.909	0.005	0.909	Excellent	
BAPQ32	I lose track of my original point when talking to people	Pragmatic Language	AC	11	7 (63.6)	4 (36.4)	0.636	0.161	0.567	Fair	Discarded
			AW	10	7 (70.0)	3 (30.0)	0.700	0.117	0.660	Good	
BAPQ33	I like to closely follow a routine while working	Rigid	AC	11	9 (81.8)	2 (18.2)	0.818	0.027	0.813	Excellent	**Included**
			AW	10	9 (90.0)	1 (10.0)	0.900	0.010	0.899	Excellent	
BAPQ34	I can tell when it is time to change topics in conversation (R)	Pragmatic Language	AC	11	9 (81.8)	2 (18.2)	0.818	0.027	0.813	Excellent	**Included**
			AW	10	8 (80.0)	2 (20.0)	0.800	0.044	0.791	Excellent	
BAPQ35	I keep doing things the way I know, even if another way might be better	Rigid	AC	11	8 (72.7)	3 (27.3)	0.727	0.081	0.703	Good	Discarded
			AW	10	8 (80.0)	2 (20.0)	0.800	0.044	0.791	Excellent	
BAPQ36	I enjoy chatting with people (R)	Aloof	AC	11	8 (72.7)	3 (27.3)	0.727	0.081	0.703	Good	Discarded
			AW	11	8 (72.7)	3 (27.3)	0.727	0.081	0.703	Good	

Note. BAPQ: Broad Autism Phenotype Questionnaire, AC: academics and clinicians, AW: autistic women, I-CVI: item-level content validity index, pc: probability of a chance occurance, k*: modified kappa. Agreement levels are indicated by modified kappa (k*) values: below 0.40 = poor, 0.40–0.59 = fair, 0.60–0.74 = good, and above 0.74 = excellent. Relevant items are those rated by experts as 1 ('Yes, it is relevant as is') or 2 ('Yes, it is relevant but needs to be improved'). Non-relevant items are those rated as 3 ('No, it is not relevant but it can be modified to be relevant'), 4 ('No, it is not relevant to autistic women'), or 5 ('No, it is not relevant to autism at all'). (R) signifies a reverse scored statement.

Table A8

Frequencies of response options for CATI relevance evaluations across academics, clinicians, and autistic women

Item	Label	Factor	Expert group	N	Yes, it is relevant as it is (%)	Yes, it is relevant but needs to be improved (%)	No, it is not relevant but can be modified to be relevant (%)	No, it is not relevant to autistic women / to me (%)	No, it is not relevant to autism at all (%)
CATI01	I often find myself fiddling or playing repetitively with objects (e.g. clicking pens)	Repetitive behaviours	AC	10	5 (50.00)	2 (20.00)	0 (0.00)	2 (20.00)	1 (10.00)
			AW	9	7 (77.78)	2 (22.22)	0 (0.00)	0 (0.00)	0 (0.00)
CATI02			AC	10	4 (40.00)	5 (50.00)	0 (0.00)	0 (0.00)	1 (10.00)

Item	Label	Factor	Expert group	N	Yes, it is relevant as it is (%)	Yes, it is relevant but needs to be improved (%)	No, it is not relevant but can be modified to be relevant (%)	No, it is not relevant to autistic women / to me (%)	No, it is not relevant to autism at all (%)
	I like to stick to certain routines for every-day tasks	Cognitive rigidity	AW	9	7 (77.78)	2 (22.22)	0 (0.00)	0 (0.00)	0 (0.00)
CATI03	I expend a lot of mental energy trying to fit in with others	Social camouflage	AC	10	10 (100.00)	0 (0.00)	0 (0.00)	0 (0.00)	0 (0.00)
			AW	9	5 (55.56)	0 (0.00)	1 (11.11)	3 (33.33)	0 (0.00)
CATI04	I am over-sensitive to bright lighting	Sensory sensitivity	AC	10	1 (10.00)	8 (80.00)	0 (0.00)	1 (10.00)	0 (0.00)
			AW	9	7 (77.78)	2 (22.22)	0 (0.00)	0 (0.00)	0 (0.00)
CATI05	There are certain activities that I always choose to do the same way, every time	Cognitive rigidity	AC	10	6 (60.00)	3 (30.00)	1 (10.00)	0 (0.00)	0 (0.00)
			AW	9	7 (77.78)	2 (22.22)	0 (0.00)	0 (0.00)	0 (0.00)
CATI06	Sometimes I watch people interacting and try to copy them when I need to socialise	Social camouflage	AC	10	9 (90.00)	1 (10.00)	0 (0.00)	0 (0.00)	0 (0.00)
			AW	9	5 (55.56)	3 (33.33)	0 (0.00)	1 (11.11)	0 (0.00)
CATI07	I often rock when sitting in a chair	Repetitive behaviours	AC	11	4 (36.36)	5 (45.45)	0 (0.00)	1 (9.09)	1 (9.09)
			AW	9	5 (55.56)	1 (11.11)	0 (0.00)	3 (33.33)	0 (0.00)
CATI08	I generally enjoy social events (R)	Social interactions	AC	10	3 (30.00)	4 (40.00)	0 (0.00)	2 (20.00)	1 (10.00)
			AW	9	5 (55.56)	2 (22.22)	1 (11.11)	1 (11.11)	0 (0.00)
CATI09	I look for strategies and ways to appear more sociable	Social camouflage	AC	10	7 (70.00)	3 (30.00)	0 (0.00)	0 (0.00)	0 (0.00)
			AW	9	6 (66.67)	1 (11.11)	0 (0.00)	2 (22.22)	0 (0.00)
CATI10	In social situations, I try to avoid interactions with other people	Social interactions	AC	10	4 (40.00)	3 (30.00)	0 (0.00)	1 (10.00)	2 (20.00)
			AW	9	5 (55.56)	3 (33.33)	1 (11.11)	0 (0.00)	0 (0.00)
CATI11	There are times when I feel that my senses are overloaded	Sensory sensitivity	AC	10	7 (70.00)	2 (20.00)	1 (10.00)	0 (0.00)	0 (0.00)
			AW	9	8 (88.89)	1 (11.11)	0 (0.00)	0 (0.00)	0 (0.00)

Item	Label	Factor	Expert group	N	Yes, it is relevant as it is (%)	Yes, it is relevant but needs to be improved (%)	No, it is not relevant but can be modified to be relevant (%)	No, it is not relevant to autistic women / to me (%)	No, it is not relevant to autism at all (%)
CATI12	There are certain objects that I fiddle or play with that can help me calm down or collect my thoughts	Repetitive behaviours	AC	10	9 (90.00)	0 (0.00)	0 (0.00)	0 (0.00)	1 (10.00)
			AW	9	6 (66.67)	1 (11.11)	0 (0.00)	2 (22.22)	0 (0.00)
CATI13	Reading non-verbal cues (e.g. facial expressions, body language) is difficult for me	Communication	AC	10	6 (60.00)	4 (40.00)	0 (0.00)	0 (0.00)	0 (0.00)
			AW	9	3 (33.33)	2 (22.22)	1 (11.11)	3 (33.33)	0 (0.00)
CATI14	I like my belongings to be sorted in certain ways and will spend time making sure they are that way	Cognitive rigidity	AC	10	4 (40.00)	4 (40.00)	2 (20.00)	0 (0.00)	0 (0.00)
			AW	9	7 (77.78)	1 (11.11)	0 (0.00)	1 (11.11)	0 (0.00)
CATI15	Social interaction is easy for me (R)	Social interactions	AC	10	6 (60.00)	4 (40.00)	0 (0.00)	0 (0.00)	0 (0.00)
			AW	9	7 (77.78)	2 (22.22)	0 (0.00)	0 (0.00)	0 (0.00)
CATI16	When interacting with other people, I spend a lot of effort monitoring how I am coming across	Social camouflage	AC	10	9 (90.00)	1 (10.00)	0 (0.00)	0 (0.00)	0 (0.00)
			AW	9	6 (66.67)	1 (11.11)	0 (0.00)	2 (22.22)	0 (0.00)
CATI17	I find social interactions stressful	Social interactions	AC	10	3 (30.00)	6 (60.00)	0 (0.00)	1 (10.00)	0 (0.00)
			AW	9	7 (77.78)	2 (22.22)	0 (0.00)	0 (0.00)	0 (0.00)
CATI18	I am over-sensitive to touch	Sensory sensitivity	AC	10	3 (30.00)	7 (70.00)	0 (0.00)	0 (0.00)	0 (0.00)
			AW	9	7 (77.78)	2 (22.22)	0 (0.00)	0 (0.00)	0 (0.00)
CATI19	I can tell how people feel from their facial expressions (R)	Communication	AC	10	3 (30.00)	6 (60.00)	1 (10.00)	0 (0.00)	0 (0.00)
			AW	9	4 (44.44)	3 (33.33)	0 (0.00)	1 (11.11)	1 (11.11)
CATI20	I have a tendency to pace or move around in a repetitive path	Repetitive behaviours	AC	10	3 (30.00)	3 (30.00)	1 (10.00)	1 (10.00)	2 (20.00)
			AW	9	6 (66.67)	0 (0.00)	1 (11.11)	2 (22.22)	0 (0.00)
CATI21	I feel discomfort when prevented from completing a particular routine	Cognitive rigidity	AC	11	9 (81.82)	1 (9.09)	0 (0.00)	1 (9.09)	0 (0.00)
			AW	9	5 (55.56)	2 (22.22)	1 (11.11)	1 (11.11)	0 (0.00)

Item	Label	Factor	Expert group	N	Yes, it is relevant as it is (%)	Yes, it is relevant but needs to be improved (%)	No, it is not relevant but can be modified to be relevant (%)	No, it is not relevant to autistic women / to me (%)	No, it is not relevant to autism at all (%)
CATI22	I rely on a set of scripts when I talk with people	Social camouflage	AC	10	5 (50.00)	5 (50.00)	0 (0.00)	0 (0.00)	0 (0.00)
			AW	9	4 (44.44)	1 (11.11)	1 (11.11)	3 (33.33)	0 (0.00)
CATI23	I find it easy to sense what someone else is feeling (R)	Communication	AC	10	6 (60.00)	4 (40.00)	0 (0.00)	0 (0.00)	0 (0.00)
			AW	9	3 (33.33)	5 (55.56)	0 (0.00)	1 (11.11)	0 (0.00)
CATI24	I am over-sensitive to particular tastes (e.g. salty, sour, spicy, or sweet)	Sensory sensitivity	AC	10	2 (20.00)	7 (70.00)	1 (10.00)	0 (0.00)	0 (0.00)
			AW	9	4 (44.44)	3 (33.33)	0 (0.00)	2 (22.22)	0 (0.00)
CATI25	I engage in certain repetitive actions when I feel stressed	Repetitive behaviours	AC	11	8 (72.73)	2 (18.18)	0 (0.00)	0 (0.00)	1 (9.09)
			AW	9	6 (66.67)	2 (22.22)	0 (0.00)	1 (11.11)	0 (0.00)
CATI26	I rarely use non-verbal cues in my interactions with others	Communication	AC	10	1 (10.00)	8 (80.00)	0 (0.00)	1 (10.00)	0 (0.00)
			AW	9	1 (11.11)	3 (33.33)	0 (0.00)	5 (55.56)	0 (0.00)
CATI27	I often insist on doing things in a certain way, or re-doing things until they are ‘just right’	Cognitive rigidity	AC	10	7 (70.00)	3 (30.00)	0 (0.00)	0 (0.00)	0 (0.00)
			AW	9	6 (66.67)	3 (33.33)	0 (0.00)	0 (0.00)	0 (0.00)
CATI28	I feel confident or capable when meeting new people (R)	Social interactions	AC	11	3 (27.27)	7 (63.64)	0 (0.00)	0 (0.00)	1 (9.09)
			AW	9	3 (33.33)	4 (44.44)	1 (11.11)	1 (11.11)	0 (0.00)
CATI29	Before engaging in a social situation, I will create a script to follow where possible	Social camouflage	AC	10	6 (60.00)	4 (40.00)	0 (0.00)	0 (0.00)	0 (0.00)
			AW	9	4 (44.44)	3 (33.33)	0 (0.00)	2 (22.22)	0 (0.00)
CATI30	Social occasions are often challenging for me	Social interactions	AC	10	8 (80.00)	2 (20.00)	0 (0.00)	0 (0.00)	0 (0.00)
			AW	9	8 (88.89)	1 (11.11)	0 (0.00)	0 (0.00)	0 (0.00)
CATI31	Sometimes the presence of a smell makes it hard for me to focus on anything else	Sensory sensitivity	AC	11	7 (63.64)	4 (36.36)	0 (0.00)	0 (0.00)	0 (0.00)
			AW	9	8 (88.89)	0 (0.00)	0 (0.00)	1 (11.11)	0 (0.00)

Item	Label	Factor	Expert group	N	Yes, it is relevant as it is (%)	Yes, it is relevant but needs to be improved (%)	No, it is not relevant but can be modified to be relevant (%)	No, it is not relevant to autistic women / to me (%)	No, it is not relevant to autism at all (%)
CATI32	There are certain repetitive actions that others consider to be characteristic of me (e.g. stroking my hair)	Repetitive behaviours	AC	10	4 (40.00)	5 (50.00)	0 (0.00)	0 (0.00)	1 (10.00)
			AW	9	7 (77.78)	1 (11.11)	0 (0.00)	1 (11.11)	0 (0.00)
CATI33	Metaphors or 'figures of speech' often confuse me	Communication	AC	10	6 (60.00)	3 (30.00)	0 (0.00)	1 (10.00)	0 (0.00)
			AW	9	5 (55.56)	0 (0.00)	0 (0.00)	4 (44.44)	0 (0.00)
CATI34	It annoys me when plans I have made are changed	Cognitive rigidity	AC	10	3 (30.00)	7 (70.00)	0 (0.00)	0 (0.00)	0 (0.00)
			AW	9	6 (66.67)	3 (33.33)	0 (0.00)	0 (0.00)	0 (0.00)
CATI35	I find it difficult to make new friends	Social interactions	AC	10	8 (80.00)	1 (10.00)	0 (0.00)	1 (10.00)	0 (0.00)
			AW	9	6 (66.67)	3 (33.33)	0 (0.00)	0 (0.00)	0 (0.00)
CATI36	I react poorly to unexpected loud noises	Sensory sensitivity	AC	10	0 (0.00)	10 (100.00)	0 (0.00)	0 (0.00)	0 (0.00)
			AW	9	6 (66.67)	2 (22.22)	0 (0.00)	1 (11.11)	0 (0.00)
CATI37	I have difficulty understanding someone else's point-of-view	Communication	AC	10	7 (70.00)	2 (20.00)	1 (10.00)	0 (0.00)	0 (0.00)
			AW	9	2 (22.22)	4 (44.44)	0 (0.00)	3 (33.33)	0 (0.00)
CATI38	I like to arrange items in rows or patterns	Cognitive rigidity	AC	10	4 (40.00)	4 (40.00)	1 (10.00)	1 (10.00)	0 (0.00)
			AW	9	6 (66.67)	2 (22.22)	1 (11.11)	0 (0.00)	0 (0.00)
CATI39	I try to follow certain rule in order to get by in social situations	Social camouflage	AC	10	8 (80.00)	2 (20.00)	0 (0.00)	0 (0.00)	0 (0.00)
			AW	9	5 (55.56)	2 (22.22)	1 (11.11)	1 (11.11)	0 (0.00)
CATI40	I am sensitive to flickering lights	Sensory sensitivity	AC	10	5 (50.00)	3 (30.00)	1 (10.00)	1 (10.00)	0 (0.00)
			AW	9	8 (88.89)	1 (11.11)	0 (0.00)	0 (0.00)	0 (0.00)
CATI41	I have certain habits that I find difficult to stop (e.g. biting/tearing nails, pulling strands of hair)	Repetitive behaviours	AC	10	4 (40.00)	2 (20.00)	1 (10.00)	2 (20.00)	1 (10.00)
			AW	9	7 (77.78)	1 (11.11)	0 (0.00)	1 (11.11)	0 (0.00)

Item	Label	Factor	Expert group	N	Yes, it is relevant as it is (%)	Yes, it is relevant but needs to be improved (%)	No, it is not relevant but can be modified to be relevant (%)	No, it is not relevant to autistic women / to me (%)	No, it is not relevant to autism at all (%)
CATI42	I have difficulty understanding the unspoken rules of social situations	Communication	AC	10	9 (90.00)	1 (10.00)	0 (0.00)	0 (0.00)	0 (0.00)
			AW	9	6 (66.67)	3 (33.33)	0 (0.00)	0 (0.00)	0 (0.00)

Note. CATI: Comprehensive Autistic Trait Inventory, AC: academics and clinicians, AW: autistic women. (R) signifies a reverse scored statement.

Table A9

Frequencies of relevant and non-relevant response options, I-CVI, p_c, and k^* per item for CATI across academics and clinicians and autistic women

Item	Label	Factor	Expert group	N	Relevant (%)	Not Relevant (%)	I-CVI	pc	k*	Interpretation	Decision
CATI01	I often find myself fiddling or playing repetitively with objects (e.g. clicking pens)	Repetitive behaviours	AC	10	7 (70.0)	3 (30.0)	0.700	0.117	0.660	Good	Discarded
			AW	9	9 (100.0)	0 (0.0)	1.000	0.002	1.000	Excellent	
CATI02	I like to stick to certain routines for every-day tasks	Cognitive rigidity	AC	10	9 (90.0)	1 (10.0)	0.900	0.010	0.899	Excellent	**Included**
			AW	9	9 (100.0)	0 (0.0)	1.000	0.002	1.000	Excellent	
CATI03	I expend a lot of mental energy trying to fit in with others	Social camouflage	AC	10	10 (100.0)	0 (0.0)	1.000	0.001	1.000	Excellent	Discarded
			AW	9	5 (55.6)	4 (44.4)	0.556	0.246	0.410	Fair	
CATI04	I am over-sensitive to bright lighting	Sensory sensitivity	AC	10	9 (90.0)	1 (10.0)	0.900	0.010	0.899	Excellent	**Included**
			AW	9	9 (100.0)	0 (0.0)	1.000	0.002	1.000	Excellent	
CATI05	There are certain activities that I always choose to do the same way, every time	Cognitive rigidity	AC	10	9 (90.0)	1 (10.0)	0.900	0.010	0.899	Excellent	**Included**
			AW	9	9 (100.0)	0 (0.0)	1.000	0.002	1.000	Excellent	
CATI06	Sometimes I watch people interacting and try to copy them when I need to socialise	Social camouflage	AC	10	10 (100.0)	0 (0.0)	1.000	0.001	1.000	Excellent	**Included**
			AW	9	8 (88.9)	1 (11.1)	0.889	0.018	0.887	Excellent	
CATI07			AC	11	9 (81.8)	2 (18.2)	0.818	0.027	0.813	Excellent	Discarded

Item	Label	Factor	Expert group	N	Relevant (%)	Not Relevant (%)	I-CVI	pc	k*	Interpretation	Decision
	I often rock when sitting in a chair	Repetitive behaviours	AW	9	6 (66.7)	3 (33.3)	0.667	0.164	0.601	Good	
CATI08	I generally enjoy social events (R)	Social interactions	AC	10	7 (70.0)	3 (30.0)	0.700	0.117	0.660	Good	Discarded
			AW	9	7 (77.8)	2 (22.2)	0.778	0.070	0.761	Excellent	
CATI09	I look for strategies and ways to appear more sociable	Social camouflage	AC	10	10 (100.0)	0 (0.0)	1.000	0.001	1.000	Excellent	**Included**
			AW	9	7 (77.8)	2 (22.2)	0.778	0.070	0.761	Excellent	
CATI10	In social situations, I try to avoid interactions with other people	Social interactions	AC	10	7 (70.0)	3 (30.0)	0.700	0.117	0.660	Good	Discarded
			AW	9	8 (88.9)	1 (11.1)	0.889	0.018	0.887	Excellent	
CATI11	There are times when I feel that my senses are overloaded	Sensory sensitivity	AC	10	9 (90.0)	1 (10.0)	0.900	0.010	0.899	Excellent	**Included**
			AW	9	9 (100.0)	0 (0.0)	1.000	0.002	1.000	Excellent	
CATI12	There are certain objects that I fiddle or play with that can help me calm down or collect my thoughts	Repetitive behaviours	AC	10	9 (90.0)	1 (10.0)	0.900	0.010	0.899	Excellent	**Included**
			AW	9	7 (77.8)	2 (22.2)	0.778	0.070	0.761	Excellent	
CATI13	Reading non-verbal cues (e.g. facial expressions, body language) is difficult for me	Communication	AC	10	10 (100.0)	0 (0.0)	1.000	0.001	1.000	Excellent	Discarded
			AW	9	5 (55.6)	4 (44.4)	0.556	0.246	0.410	Fair	
CATI14	I like my belongings to be sorted in certain ways and will spend time making sure they are that way	Cognitive rigidity	AC	10	8 (80.0)	2 (20.0)	0.800	0.044	0.791	Excellent	**Included**
			AW	9	8 (88.9)	1 (11.1)	0.889	0.018	0.887	Excellent	
CATI15	Social interaction is easy for me (R)	Social interactions	AC	10	10 (100.0)	0 (0.0)	1.000	0.001	1.000	Excellent	**Included**
			AW	9	9 (100.0)	0 (0.0)	1.000	0.002	1.000	Excellent	
CATI16	When interacting with other people, I spend a lot of effort monitoring how I am coming across	Social camouflage	AC	10	10 (100.0)	0 (0.0)	1.000	0.001	1.000	Excellent	**Included**
			AW	9	7 (77.8)	2 (22.2)	0.778	0.070	0.761	Excellent	
CATI17			AC	10	9 (90.0)	1 (10.0)	0.900	0.010	0.899	Excellent	**Included**

Item	Label	Factor	Expert group	N	Relevant (%)	Not Relevant (%)	I-CVI	pc	k*	Interpretation	Decision
	I find social interactions stressful	Social interactions	AW	9	9 (100.0)	0 (0.0)	1.000	0.002	1.000	Excellent	
CATI18	I am over-sensitive to touch	Sensory sensitivity	AC	10	10 (100.0)	0 (0.0)	1.000	0.001	1.000	Excellent	**Included**
			AW	9	9 (100.0)	0 (0.0)	1.000	0.002	1.000	Excellent	
CATI19	I can tell how people feel from their facial expressions (R)	Communication	AC	10	9 (90.0)	1 (10.0)	0.900	0.010	0.899	Excellent	**Included**
			AW	9	7 (77.8)	2 (22.2)	0.778	0.070	0.761	Excellent	
CATI20	I have a tendency to pace or move around in a repetitive path	Repetitive behaviours	AC	10	6 (60.0)	4 (40.0)	0.600	0.205	0.497	Fair	Discarded
			AW	9	6 (66.7)	3 (33.3)	0.667	0.164	0.601	Good	
CATI21	I feel discomfort when prevented from completing a particular routine	Cognitive rigidity	AC	11	10 (90.9)	1 (9.1)	0.909	0.005	0.909	Excellent	**Included**
			AW	9	7 (77.8)	2 (22.2)	0.778	0.070	0.761	Excellent	
CATI22	I rely on a set of scripts when I talk with people	Social camouflage	AC	10	10 (100.0)	0 (0.0)	1.000	0.001	1.000	Excellent	Discarded
			AW	9	5 (55.6)	4 (44.4)	0.556	0.246	0.410	Fair	
CATI23	I find it easy to sense what someone else is feeling (R)	Communication	AC	10	10 (100.0)	0 (0.0)	1.000	0.001	1.000	Excellent	**Included**
			AW	9	8 (88.9)	1 (11.1)	0.889	0.018	0.887	Excellent	
CATI24	I am over-sensitive to particular tastes (e.g. salty, sour, spicy, or sweet)	Sensory sensitivity	AC	10	9 (90.0)	1 (10.0)	0.900	0.010	0.899	Excellent	**Included**
			AW	9	7 (77.8)	2 (22.2)	0.778	0.070	0.761	Excellent	
CATI25	I engage in certain repetitive actions when I feel stressed	Repetitive behaviours	AC	11	10 (90.9)	1 (9.1)	0.909	0.005	0.909	Excellent	**Included**
			AW	9	8 (88.9)	1 (11.1)	0.889	0.018	0.887	Excellent	
CATI26	I rarely use non-verbal cues in my interactions with others	Communication	AC	10	9 (90.0)	1 (10.0)	0.900	0.010	0.899	Excellent	Discarded
			AW	9	4 (44.4)	5 (55.6)	0.444	0.246	0.263	Poor	
CATI27	I often insist on doing things in a certain	Cognitive rigidity	AC	10	10 (100.0)	0 (0.0)	1.000	0.001	1.000	Excellent	**Included**

Item	Label	Factor	Expert group	N	Relevant (%)	Not Relevant (%)	I-CVI	pc	k*	Interpretation	Decision
	way, or re-doing things until they are 'just right'		AW	9	9 (100.0)	0 (0.0)	1.000	0.002	1.000	Excellent	
CATI28	I feel confident or capable when meeting new people (R)	Social interactions	AC	11	10 (90.9)	1 (9.1)	0.909	0.005	0.909	Excellent	**Included**
			AW	9	7 (77.8)	2 (22.2)	0.778	0.070	0.761	Excellent	
CATI29	Before engaging in a social situation, I will create a script to follow where possible	Social camouflage	AC	10	10 (100.0)	0 (0.0)	1.000	0.001	1.000	Excellent	**Included**
			AW	9	7 (77.8)	2 (22.2)	0.778	0.070	0.761	Excellent	
CATI30	Social occasions are often challenging for me	Social interactions	AC	10	10 (100.0)	0 (0.0)	1.000	0.001	1.000	Excellent	**Included**
			AW	9	9 (100.0)	0 (0.0)	1.000	0.002	1.000	Excellent	
CATI31	Sometimes the presence of a smell makes it hard for me to focus on anything else	Sensory sensitivity	AC	11	11 (100.0)	0 (0.0)	1.000	0.000	1.000	Excellent	**Included**
			AW	9	8 (88.9)	1 (11.1)	0.889	0.018	0.887	Excellent	
CATI32	There are certain repetitive actions that others consider to be characteristic of me (e.g. stroking my hair)	Repetitive behaviours	AC	10	9 (90.0)	1 (10.0)	0.900	0.010	0.899	Excellent	**Included**
			AW	9	8 (88.9)	1 (11.1)	0.889	0.018	0.887	Excellent	
CATI33	Metaphors or 'figures of speech' often confuse me	Communication	AC	10	9 (90.0)	1 (10.0)	0.900	0.010	0.899	Excellent	Discarded
			AW	9	5 (55.6)	4 (44.4)	0.556	0.246	0.410	Fair	
CATI34	It annoys me when plans I have made are changed	Cognitive rigidity	AC	10	10 (100.0)	0 (0.0)	1.000	0.001	1.000	Excellent	**Included**
			AW	9	9 (100.0)	0 (0.0)	1.000	0.002	1.000	Excellent	
CATI35	I find it difficult to make new friends	Social interactions	AC	10	9 (90.0)	1 (10.0)	0.900	0.010	0.899	Excellent	**Included**
			AW	9	9 (100.0)	0 (0.0)	1.000	0.002	1.000	Excellent	
CATI36	I react poorly to unexpected loud noises	Sensory sensitivity	AC	10	10 (100.0)	0 (0.0)	1.000	0.001	1.000	Excellent	**Included**
			AW	9	8 (88.9)	1 (11.1)	0.889	0.018	0.887	Excellent	
CATI37	I have difficulty understanding	Communication	AC	10	9 (90.0)	1 (10.0)	0.900	0.010	0.899	Excellent	Discarded

Item	Label	Factor	Expert group	N	Relevant (%)	Not Relevant (%)	I-CVI	pc	k*	Interpretation	Decision
	someone else's point-of-view		AW	9	6 (66.7)	3 (33.3)	0.667	0.164	0.601	Good	
CATI38	I like to arrange items in rows or patterns	Cognitive rigidity	AC	10	8 (80.0)	2 (20.0)	0.800	0.044	0.791	Excellent	**Included**
			AW	9	8 (88.9)	1 (11.1)	0.889	0.018	0.887	Excellent	
CATI39	I try to follow certain rule in order to get by in social situations	Social camouflage	AC	10	10 (100.0)	0 (0.0)	1.000	0.001	1.000	Excellent	**Included**
			AW	9	7 (77.8)	2 (22.2)	0.778	0.070	0.761	Excellent	
CATI40	I am sensitive to flickering lights	Sensory sensitivity	AC	10	8 (80.0)	2 (20.0)	0.800	0.044	0.791	Excellent	**Included**
			AW	9	9 (100.0)	0 (0.0)	1.000	0.002	1.000	Excellent	
CATI41	I have certain habits that I find difficult to stop (e.g. biting/tearing nails, pulling strands of hair)	Repetitive behaviours	AC	10	6 (60.0)	4 (40.0)	0.600	0.205	0.497	Fair	Discarded
			AW	9	8 (88.9)	1 (11.1)	0.889	0.018	0.887	Excellent	
CATI42	I have difficulty understanding the unspoken rules of social situations	Communication	AC	10	10 (100.0)	0 (0.0)	1.000	0.001	1.000	Excellent	**Included**
			AW	9	9 (100.0)	0 (0.0)	1.000	0.002	1.000	Excellent	

Note. CATI: Comprehensive Autistic Trait Inventory, AC: academics and clinicians, AW: autistic women, I-CVI: item-level content validity index, pc: probability of a chance occurance, k*: modified kappa. Agreement levels are indicated by modified kappa (k*) values: below 0.40 = poor, 0.40–0.59 = fair, 0.60–0.74 = good, and above 0.74 = excellent. Relevant items are those rated by experts as 1 ('Yes, it is relevant as is') or 2 ('Yes, it is relevant but needs to be improved'). Non-relevant items are those rated as 3 ('No, it is not relevant but it can be modified to be relevant'), 4 ('No, it is not relevant to autistic women'), or 5 ('No, it is not relevant to autism at all'). (R) signifies a reverse scored statement.

Table A10

Frequencies of response options for GQ-ASC relevance evaluations across academics, clinicians, and autistic women

Item	Label	Factor	Expert group	N	Yes, it is relevant as it is (%)	Yes, it is relevant but needs to be improved (%)	No, it is not relevant but can be modified to be relevant (%)	No, it is not relevant to autistic women / to me (%)	No, it is not relevant to autism at all (%)
GQASC01	I enjoy fantasy worlds (R)	Imagination and play	AC	10	3 (30.00)	4 (40.00)	1 (10.00)	1 (10.00)	1 (10.00)
			AW	10	3 (30.00)	2 (20.00)	0 (0.00)	5 (50.00)	0 (0.00)
GQASC02	I am interested in fiction (R)		AC	10	2 (20.00)	2 (20.00)	0 (0.00)	3 (30.00)	3 (30.00)

Item	Label	Factor	Expert group	N	Yes, it is relevant as it is (%)	Yes, it is relevant but needs to be improved (%)	No, it is not relevant but can be modified to be relevant (%)	No, it is not relevant to autistic women / to me (%)	No, it is not relevant to autism at all (%)
		Imagination and play	AW	10	2 (20.00)	3 (30.00)	0 (0.00)	5 (50.00)	0 (0.00)
GQASC03	When I was 5-12 years old, I played as imaginatively as other girls (R)	Imagination and play	AC	10	4 (40.00)	2 (20.00)	2 (20.00)	0 (0.00)	2 (20.00)
			AW	10	2 (20.00)	1 (10.00)	1 (10.00)	6 (60.00)	0 (0.00)
GQASC04	When I was 5-12 years old, I had imaginary friends or imaginary animals (R)	Imagination and play	AC	10	4 (40.00)	2 (20.00)	0 (0.00)	2 (20.00)	2 (20.00)
			AW	10	2 (20.00)	2 (20.00)	1 (10.00)	5 (50.00)	0 (0.00)
GQASC05	When I was 5-12 years old, I created my own complex 'set ups' with toys (R)	Imagination and play	AC	10	1 (10.00)	6 (60.00)	0 (0.00)	3 (30.00)	0 (0.00)
			AW	10	6 (60.00)	1 (10.00)	0 (0.00)	3 (30.00)	0 (0.00)
GQASC06	I copy or 'clone' myself on other females	Camouflaging	AC	10	7 (70.00)	1 (10.00)	2 (20.00)	0 (0.00)	0 (0.00)
			AW	10	4 (40.00)	3 (30.00)	2 (20.00)	1 (10.00)	0 (0.00)
GQASC07	I avidly observe other females socialising	Camouflaging	AC	10	7 (70.00)	2 (20.00)	1 (10.00)	0 (0.00)	0 (0.00)
			AW	10	5 (50.00)	3 (30.00)	1 (10.00)	1 (10.00)	0 (0.00)
GQASC08	I am attracted to females with strong personalities who tell me what to do	Camouflaging	AC	10	4 (40.00)	2 (20.00)	1 (10.00)	1 (10.00)	2 (20.00)
			AW	10	1 (10.00)	1 (10.00)	2 (20.00)	4 (40.00)	2 (20.00)
GQASC09	I adopt a different persona in different situations	Camouflaging	AC	10	6 (60.00)	3 (30.00)	0 (0.00)	0 (0.00)	1 (10.00)
			AW	10	6 (60.00)	3 (30.00)	1 (10.00)	0 (0.00)	0 (0.00)
GQASC10	I am attached to certain objects or toys (e.g., favourite toy, pillow, piece of cloth) which I carry, touch, or rub to calm myself	Sensory sensitivities	AC	10	4 (40.00)	4 (40.00)	0 (0.00)	2 (20.00)	0 (0.00)
			AW	10	9 (90.00)	1 (10.00)	0 (0.00)	0 (0.00)	0 (0.00)
GQASC11	I expressed distress during grooming (e.g., I fought or cried during fingernail cutting, haircutting, combing) or when I am touched (e.g., someone touches my feet)	Sensory sensitivities	AC	10	4 (40.00)	5 (50.00)	1 (10.00)	0 (0.00)	0 (0.00)
			AW	10	4 (40.00)	3 (30.00)	0 (0.00)	3 (30.00)	0 (0.00)

Item	Label	Factor	Expert group	N	Yes, it is relevant as it is (%)	Yes, it is relevant but needs to be improved (%)	No, it is not relevant but can be modified to be relevant (%)	No, it is not relevant to autistic women / to me (%)	No, it is not relevant to autism at all (%)
GQASC12	Some social situations make me mute	Sensory sensitivities	AC	10	5 (50.00)	5 (50.00)	0 (0.00)	0 (0.00)	0 (0.00)
			AW	10	5 (50.00)	4 (40.00)	1 (10.00)	0 (0.00)	0 (0.00)
GQASC13	I am distressed by certain smells or I avoid certain tastes that are a typical part of a diet	Sensory sensitivities	AC	10	6 (60.00)	3 (30.00)	0 (0.00)	1 (10.00)	0 (0.00)
			AW	10	7 (70.00)	2 (20.00)	1 (10.00)	0 (0.00)	0 (0.00)
GQASC14	I socialise quite well for a while, but subsequently feel exhausted	Socializing	AC	10	9 (90.00)	1 (10.00)	0 (0.00)	0 (0.00)	0 (0.00)
			AW	10	7 (70.00)	1 (10.00)	1 (10.00)	1 (10.00)	0 (0.00)
GQASC15	I often have a facial 'mask' that hides my social confusion	Socializing	AC	10	6 (60.00)	4 (40.00)	0 (0.00)	0 (0.00)	0 (0.00)
			AW	10	6 (60.00)	4 (40.00)	0 (0.00)	0 (0.00)	0 (0.00)
GQASC16	I have intense emotions	Socializing	AC	10	7 (70.00)	2 (20.00)	1 (10.00)	0 (0.00)	0 (0.00)
			AW	10	8 (80.00)	2 (20.00)	0 (0.00)	0 (0.00)	0 (0.00)
GQASC17	I apologise when I make a social error	Socializing	AC	10	4 (40.00)	3 (30.00)	1 (10.00)	0 (0.00)	2 (20.00)
			AW	10	3 (30.00)	6 (60.00)	0 (0.00)	1 (10.00)	0 (0.00)
GQASC18	When I was 5-12 years old, I preferred to play with girls' toys (R)	Interests	AC	10	1 (10.00)	3 (30.00)	0 (0.00)	3 (30.00)	3 (30.00)
			AW	10	3 (30.00)	0 (0.00)	4 (40.00)	2 (20.00)	1 (10.00)
GQASC19	When I was 5-12 years old, I preferred to play with boys' toys	Interests	AC	10	2 (20.00)	1 (10.00)	2 (20.00)	3 (30.00)	2 (20.00)
			AW	10	4 (40.00)	1 (10.00)	2 (20.00)	2 (20.00)	1 (10.00)
GQASC20	My interests were advanced for my age (e.g., opera)	Interests	AC	10	2 (20.00)	5 (50.00)	1 (10.00)	2 (20.00)	0 (0.00)
			AW	10	3 (30.00)	1 (10.00)	2 (20.00)	4 (40.00)	0 (0.00)
GQASC21	I am talented in music	Interests	AC	10	2 (20.00)	0 (0.00)	0 (0.00)	2 (20.00)	6 (60.00)

Item	Label	Factor	Expert group	N	Yes, it is relevant as it is (%)	Yes, it is relevant but needs to be improved (%)	No, it is not relevant but can be modified to be relevant (%)	No, it is not relevant to autistic women / to me (%)	No, it is not relevant to autism at all (%)
			AW	10	2 (20.00)	2 (20.00)	1 (10.00)	3 (30.00)	2 (20.00)

Note. GQASC: Girls Questionnaire for Autism Spectrum Condition, AC: academics and clinicians, AW: autistic women. (R) signifies a reverse scored statement.

Table A11

Frequencies of relevant and non-relevant response options, I-CVI, p_c, and k^* per item for GQ-ASC across academics and clinicians and autistic women

Item	Label	Factor	Expert group	N	Relevant (%)	Not Relevant (%)	I-CVI	pc	k*	Interpretation	Decision
GQASC01	I enjoy fantasy worlds (R)	Imagination and play	AC	10	7 (70.0)	3 (30.0)	0.7	0.117	0.660	Good	Discarded
			AW	10	5 (50.0)	5 (50.0)	0.5	0.246	0.337	Poor	
GQASC02	I am interested in fiction (R)	Imagination and play	AC	10	4 (40.0)	6 (60.0)	0.4	0.205	0.245	Poor	Discarded
			AW	10	5 (50.0)	5 (50.0)	0.5	0.246	0.337	Poor	
GQASC03	When I was 5-12 years old, I played as imaginatively as other girls (R)	Imagination and play	AC	10	6 (60.0)	4 (40.0)	0.6	0.205	0.497	Fair	Discarded
			AW	10	3 (30.0)	7 (70.0)	0.3	0.117	0.207	Poor	
GQASC04	When I was 5-12 years old, I had imaginary friends or imaginary animals (R)	Imagination and play	AC	10	6 (60.0)	4 (40.0)	0.6	0.205	0.497	Fair	Discarded
			AW	10	4 (40.0)	6 (60.0)	0.4	0.205	0.245	Poor	
GQASC05	When I was 5-12 years old, I created my own complex ‘set ups’ with toys (R)	Imagination and play	AC	10	7 (70.0)	3 (30.0)	0.7	0.117	0.660	Good	Discarded
			AW	10	7 (70.0)	3 (30.0)	0.7	0.117	0.660	Good	
GQASC06	I copy or ‘clone’ myself on other females	Camouflaging	AC	10	8 (80.0)	2 (20.0)	0.8	0.044	0.791	Excellent	Discarded
			AW	10	7 (70.0)	3 (30.0)	0.7	0.117	0.660	Good	
GQASC07	I avidly observe other females socialising	Camouflaging	AC	10	9 (90.0)	1 (10.0)	0.9	0.010	0.899	Excellent	**Included**
			AW	10	8 (80.0)	2 (20.0)	0.8	0.044	0.791	Excellent	

Item	Label	Factor	Expert group	N	Relevant (%)	Not Relevant (%)	I-CVI	pc	k*	Interpretation	Decision
GQASC08	I am attracted to females with strong personalities who tell me what to do	Camouflaging	AC	10	6 (60.0)	4 (40.0)	0.6	0.205	0.497	Fair	Discarded
			AW	10	2 (20.0)	8 (80.0)	0.2	0.044	0.163	Poor	
GQASC09	I adopt a different persona in different situations	Camouflaging	AC	10	9 (90.0)	1 (10.0)	0.9	0.010	0.899	Excellent	**Included**
			AW	10	9 (90.0)	1 (10.0)	0.9	0.010	0.899	Excellent	
GQASC10	I am attached to certain objects or toys (e.g., favourite toy, pillow, piece of cloth) which I carry, touch, or rub to calm myself	Sensory sensitivities	AC	10	8 (80.0)	2 (20.0)	0.8	0.044	0.791	Excellent	**Included**
			AW	10	10 (100.0)	0 (0.0)	1.0	0.001	1.000	Excellent	
GQASC11	I expressed distress during grooming (e.g., I fought or cried during fingernail cutting, haircutting, combing) or when I am touched (e.g., someone touches my feet)	Sensory sensitivities	AC	10	9 (90.0)	1 (10.0)	0.9	0.010	0.899	Excellent	Discarded
			AW	10	7 (70.0)	3 (30.0)	0.7	0.117	0.660	Good	
GQASC12	Some social situations make me mute	Sensory sensitivities	AC	10	10 (100.0)	0 (0.0)	1.0	0.001	1.000	Excellent	**Included**
			AW	10	9 (90.0)	1 (10.0)	0.9	0.010	0.899	Excellent	
GQASC13	I am distressed by certain smells or I avoid certain tastes that are a typical part of a diet	Sensory sensitivities	AC	10	9 (90.0)	1 (10.0)	0.9	0.010	0.899	Excellent	**Included**
			AW	10	9 (90.0)	1 (10.0)	0.9	0.010	0.899	Excellent	
GQASC14	I socialise quite well for a while, but subsequently feel exhausted	Socializing	AC	10	10 (100.0)	0 (0.0)	1.0	0.001	1.000	Excellent	**Included**
			AW	10	8 (80.0)	2 (20.0)	0.8	0.044	0.791	Excellent	
GQASC15	I often have a facial 'mask' that hides my social confusion	Socializing	AC	10	10 (100.0)	0 (0.0)	1.0	0.001	1.000	Excellent	**Included**
			AW	10	10 (100.0)	0 (0.0)	1.0	0.001	1.000	Excellent	
GQASC16	I have intense emotions	Socializing	AC	10	9 (90.0)	1 (10.0)	0.9	0.010	0.899	Excellent	**Included**
			AW	10	10 (100.0)	0 (0.0)	1.0	0.001	1.000	Excellent	

Item	Label	Factor	Expert group	N	Relevant (%)	Not Relevant (%)	I-CVI	pc	k*	Interpretation	Decision
GQASC17	I apologise when I make a social error	Socializing	AC	10	7 (70.0)	3 (30.0)	0.7	0.117	0.660	Good	Discarded
			AW	10	9 (90.0)	1 (10.0)	0.9	0.010	0.899	Excellent	
GQASC18	When I was 5-12 years old, I preferred to play with girls' toys (R)	Interests	AC	10	4 (40.0)	6 (60.0)	0.4	0.205	0.245	Poor	Discarded
			AW	10	3 (30.0)	7 (70.0)	0.3	0.117	0.207	Poor	
GQASC19	When I was 5-12 years old, I preferred to play with boys' toys	Interests	AC	10	3 (30.0)	7 (70.0)	0.3	0.117	0.207	Poor	Discarded
			AW	10	5 (50.0)	5 (50.0)	0.5	0.246	0.337	Poor	
GQASC20	My interests were advanced for my age (e.g., opera)	Interests	AC	10	7 (70.0)	3 (30.0)	0.7	0.117	0.660	Good	Discarded
			AW	10	4 (40.0)	6 (60.0)	0.4	0.205	0.245	Poor	
GQASC21	I am talented in music	Interests	AC	10	2 (20.0)	8 (80.0)	0.2	0.044	0.163	Poor	Discarded
			AW	10	4 (40.0)	6 (60.0)	0.4	0.205	0.245	Poor	

Note. GQASC: Girls Questionnaire for Autism Spectrum Condition, AC: academics and clinicians, AW: autistic women, I-CVI: item-level content validity index, pc: probability of a chance occurance, k*: modified kappa. Agreement levels are indicated by modified kappa (k*) values: below 0.40 = poor, 0.40–0.59 = fair, 0.60–0.74 = good, and above 0.74 = excellent. Relevant items are those rated by experts as 1 ('Yes, it is relevant as is') or 2 ('Yes, it is relevant but needs to be improved'). Non-relevant items are those rated as 3 ('No, it is not relevant but it can be modified to be relevant'), 4 ('No, it is not relevant to autistic women'), or 5 ('No, it is not relevant to autism at all'). (R) signifies a reverse scored statement.

Table A12

Frequencies of response options for RAADS-14 relevance evaluations across academics, clinicians, and autistic women

Item	Label	Factor	Expert group	N	Yes, it is relevant as it is (%)	Yes, it is relevant but needs to be improved (%)	No, it is not relevant but can be modified to be relevant (%)	No, it is not relevant to autistic women / to me (%)	No, it is not relevant to autism at all (%)
RAADS01	It is difficult for me to understand how other people are feeling when we are talking	Mentalizing deficits	AC	10	7 (70.00)	3 (30.00)	0 (0.00)	0 (0.00)	0 (0.00)
			AW	10	4 (40.00)	5 (50.00)	0 (0.00)	1 (10.00)	0 (0.00)
RAADS02	Some ordinary textures that do not bother others feel very offensive when they touch my skin	Sensory reactivity	AC	10	3 (30.00)	7 (70.00)	0 (0.00)	0 (0.00)	0 (0.00)
			AW	11	10 (90.91)	0 (0.00)	0 (0.00)	1 (9.09)	0 (0.00)

Item	Label	Factor	Expert group	N	Yes, it is relevant as it is (%)	Yes, it is relevant but needs to be improved (%)	No, it is not relevant but can be modified to be relevant (%)	No, it is not relevant to autistic women / to me (%)	No, it is not relevant to autism at all (%)
RAADS03	It is very difficult for me to work and function in groups	Social anxiety	AC	10	2 (20.00)	5 (50.00)	3 (30.00)	0 (0.00)	0 (0.00)
			AW	11	7 (63.64)	4 (36.36)	0 (0.00)	0 (0.00)	0 (0.00)
RAADS04	It is difficult to figure out what other people expect of me	Mentalizing deficits	AC	10	9 (90.00)	1 (10.00)	0 (0.00)	0 (0.00)	0 (0.00)
			AW	11	9 (81.82)	2 (18.18)	0 (0.00)	0 (0.00)	0 (0.00)
RAADS05	I often don't know how to act in social situations	Social anxiety	AC	10	4 (40.00)	6 (60.00)	0 (0.00)	0 (0.00)	0 (0.00)
			AW	10	8 (80.00)	2 (20.00)	0 (0.00)	0 (0.00)	0 (0.00)
RAADS06	I can chat and make small talk with people (R)	Social anxiety	AC	10	2 (20.00)	6 (60.00)	2 (20.00)	0 (0.00)	0 (0.00)
			AW	10	7 (70.00)	3 (30.00)	0 (0.00)	0 (0.00)	0 (0.00)
RAADS07	When I feel overwhelmed by my senses, I have to isolate myself to shut them down	Sensory reactivity	AC	10	4 (40.00)	6 (60.00)	0 (0.00)	0 (0.00)	0 (0.00)
			AW	11	7 (63.64)	4 (36.36)	0 (0.00)	0 (0.00)	0 (0.00)
RAADS08	How to make friends and socialize is a mystery to me	Social anxiety	AC	10	1 (10.00)	8 (80.00)	1 (10.00)	0 (0.00)	0 (0.00)
			AW	10	5 (50.00)	4 (40.00)	1 (10.00)	0 (0.00)	0 (0.00)
RAADS09	When talking to someone, I have a hard time telling when it is my turn to talk or to listen	Mentalizing deficits	AC	10	9 (90.00)	1 (10.00)	0 (0.00)	0 (0.00)	0 (0.00)
			AW	10	6 (60.00)	4 (40.00)	0 (0.00)	0 (0.00)	0 (0.00)
RAADS10	Sometimes I have to cover my ears to block out painful noises (like vacuum cleaners or people talking too much or too loudly)	Sensory reactivity	AC	10	4 (40.00)	6 (60.00)	0 (0.00)	0 (0.00)	0 (0.00)
			AW	10	7 (70.00)	1 (10.00)	0 (0.00)	2 (20.00)	0 (0.00)
RAADS11	It can be very hard to read someone's face, hand, and body movements when we are talking	Mentalizing deficits	AC	10	4 (40.00)	6 (60.00)	0 (0.00)	0 (0.00)	0 (0.00)
			AW	10	4 (40.00)	3 (30.00)	0 (0.00)	3 (30.00)	0 (0.00)
RAADS12	I focus on details rather than the overall idea	Mentalizing deficits	AC	10	8 (80.00)	2 (20.00)	0 (0.00)	0 (0.00)	0 (0.00)
			AW	10	6 (60.00)	2 (20.00)	0 (0.00)	2 (20.00)	0 (0.00)

Item	Label	Factor	Expert group	N	Yes, it is relevant as it is (%)	Yes, it is relevant but needs to be improved (%)	No, it is not relevant but can be modified to be relevant (%)	No, it is not relevant to autistic women / to me (%)	No, it is not relevant to autism at all (%)
RAADS13	I take things too literally, so I often miss what people are trying to say	Mentalizing deficits	AC	10	2 (20.00)	8 (80.00)	0 (0.00)	0 (0.00)	0 (0.00)
			AW	10	5 (50.00)	4 (40.00)	1 (10.00)	0 (0.00)	0 (0.00)
RAADS14	I get extremely upset when the way I like to do things is suddenly changed	Mentalizing deficits	AC	10	8 (80.00)	2 (20.00)	0 (0.00)	0 (0.00)	0 (0.00)
			AW	11	8 (72.73)	3 (27.27)	0 (0.00)	0 (0.00)	0 (0.00)

Note. RAADS: Ritvo Autism & Asperger Diagnostic Scale-14, AC: academics and clinicians, AW: autistic women. (R) signifies a reverse scored statement.

Table A13

Frequencies of relevant and non-relevant response options, I-CVI, p_c, and k^* per item for RAADS-14 across academics and clinicians and autistic women

Item	Label	Factor	Expert group	N	Relevant (%)	Not Relevant (%)	I-CVI	pc	k*	Interpretation	Decision
RAADS01	It is difficult for me to understand how other people are feeling when we are talking	Mentalizing deficits	AC	10	10 (100.0)	0 (0.0)	1.000	0.001	1.000	Excellent	**Included**
			AW	10	9 (90.0)	1 (10.0)	0.900	0.010	0.899	Excellent	
RAADS02	Some ordinary textures that do not bother others feel very offensive when they touch my skin	Sensory reactivity	AC	10	10 (100.0)	0 (0.0)	1.000	0.001	1.000	Excellent	**Included**
			AW	11	10 (90.9)	1 (9.1)	0.909	0.005	0.909	Excellent	
RAADS03	It is very difficult for me to work and function in groups	Social anxiety	AC	10	7 (70.0)	3 (30.0)	0.700	0.117	0.660	Good	Discarded
			AW	11	11 (100.0)	0 (0.0)	1.000	0.000	1.000	Excellent	
RAADS04	It is difficult to figure out what other people expect of me	Mentalizing deficits	AC	10	10 (100.0)	0 (0.0)	1.000	0.001	1.000	Excellent	**Included**
			AW	11	11 (100.0)	0 (0.0)	1.000	0.000	1.000	Excellent	
RAADS05	I often don't know how to act in social situations	Social anxiety	AC	10	10 (100.0)	0 (0.0)	1.000	0.001	1.000	Excellent	**Included**
			AW	10	10 (100.0)	0 (0.0)	1.000	0.001	1.000	Excellent	
RAADS06			AC	10	8 (80.0)	2 (20.0)	0.800	0.044	0.791	Excellent	**Included**

Item	Label	Factor	Expert group	N	Relevant (%)	Not Relevant (%)	I-CVI	pc	k*	Interpretation	Decision
	I can chat and make small talk with people (R)	Social anxiety	AW	10	10 (100.0)	0 (0.0)	1.000	0.001	1.000	Excellent	
RAADS07	When I feel overwhelmed by my senses, I have to isolate myself to shut them down	Sensory reactivity	AC	10	10 (100.0)	0 (0.0)	1.000	0.001	1.000	Excellent	**Included**
			AW	11	11 (100.0)	0 (0.0)	1.000	0.000	1.000	Excellent	
RAADS08	How to make friends and socialize is a mystery to me	Social anxiety	AC	10	9 (90.0)	1 (10.0)	0.900	0.010	0.899	Excellent	**Included**
			AW	10	9 (90.0)	1 (10.0)	0.900	0.010	0.899	Excellent	
RAADS09	When talking to someone, I have a hard time telling when it is my turn to talk or to listen	Mentalizing deficits	AC	10	10 (100.0)	0 (0.0)	1.000	0.001	1.000	Excellent	**Included**
			AW	10	10 (100.0)	0 (0.0)	1.000	0.001	1.000	Excellent	
RAADS10	Sometimes I have to cover my ears to block out painful noises (like vacuum cleaners or people talking too much or too loudly)	Sensory reactivity	AC	10	10 (100.0)	0 (0.0)	1.000	0.001	1.000	Excellent	**Included**
			AW	10	8 (80.0)	2 (20.0)	0.800	0.044	0.791	Excellent	
RAADS11	It can be very hard to read someone's face, hand, and body movements when we are talking	Mentalizing deficits	AC	10	10 (100.0)	0 (0.0)	1.000	0.001	1.000	Excellent	Discarded
			AW	10	7 (70.0)	3 (30.0)	0.700	0.117	0.660	Good	
RAADS12	I focus on details rather than the overall idea	Mentalizing deficits	AC	10	10 (100.0)	0 (0.0)	1.000	0.001	1.000	Excellent	**Included**
			AW	10	8 (80.0)	2 (20.0)	0.800	0.044	0.791	Excellent	
RAADS13	I take things too literally, so I often miss what people are trying to say	Mentalizing deficits	AC	10	10 (100.0)	0 (0.0)	1.000	0.001	1.000	Excellent	**Included**
			AW	10	9 (90.0)	1 (10.0)	0.900	0.010	0.899	Excellent	
RAADS14	I get extremely upset when the way I like to do things is suddenly changed	Mentalizing deficits	AC	10	10 (100.0)	0 (0.0)	1.000	0.001	1.000	Excellent	**Included**
			AW	11	11 (100.0)	0 (0.0)	1.000	0.000	1.000	Excellent	

Note. RAADS: Ritvo Autism & Asperger Diagnostic Scale-14, AC: academics and clinicians, AW: autistic women, I-CVI: item-level content validity index, pc: probability of a chance occurrence, k*: modified kappa. Agreement levels are indicated by modified kappa (k*) values: below 0.40 = poor, 0.40–0.59 = fair, 0.60–0.74 = good, and above 0.74 = excellent. Relevant items are those rated by experts as 1 ('Yes, it is relevant as is') or 2 ('Yes, it is relevant but needs to be improved'). Non-relevant items are those rated as 3 ('No, it is not relevant but it can be modified to be relevant'), 4 ('No, it is not relevant to autistic women'), or 5 ('No, it is not relevant to autism at all'). (R) signifies a reverse scored statement.

Table A14

Frequencies of expert ratings on each questionnaire's coverage of autistic experiences relevant to autistic women, along with I-CVI, p_c and k^* across expert groups

Scale	Expert group	N	Not missing (%)	Missing (%)	I-CVI	p_c	k*	Interpretation
AQ	AC	9	0 (0.0)	9 (100.0)	0.00	0.002	-0.002	Poor
	AW	10	3 (30.0)	7 (70.0)	0.300	0.117	0.207	Poor
BAPQ	AC	8	2 (25.0)	6 (75.0)	0.25	0.109	0.158	Poor
	AW	10	5 (50.0)	5 (50.0)	0.500	0.246	0.337	Poor
CATI	AC	10	5 (50.0)	5 (50.0)	0.50	0.246	0.337	Poor
	AW	9	4 (44.4)	5 (55.6)	0.444	0.246	0.263	Poor
RAADS14	AC	10	3 (30.0)	7 (70.0)	0.30	0.117	0.207	Poor
	AW	10	5 (50.0)	5 (50.0)	0.500	0.246	0.337	Poor
GQASC	AC	10	2 (20.0)	8 (80.0)	0.20	0.044	0.163	Poor
	AW	10	3 (30.0)	7 (70.0)	0.300	0.117	0.207	Poor

Note. AQ: Autism Spectrum Quotient, BAPQ: Broad Autism Phenotype Questionnaire, CATI: The Comprehensive Autistic Trait Inventory, RAADS-14: Ritvo Autism Asperger Diagnostic Scale 14, GQ-ASC: the Girls Questionnaire for Autism Spectrum Condition. I-CVI: item-level content validity index, pc: probability of a chance occurrence, k*: modified kappa. Agreement levels are indicated by modified kappa (k*) values: below 0.40 = poor, 0.40–0.59 = fair, 0.60–0.74 = good, and above 0.74 = excellent.

Table A15

Frequencies of expert ratings on the relevance of each questionnaire's response scale, along with I-CVI, p_c and k^* across expert groups

Scale	Expert group	N	Relevant (%) [1]	Somewhat relevant (%) [1]	Not relevant (%)	I-CVI	p_c	k*	Interpretation
AQ	AC	9	3 (33.3)	6 (66.7)	0 (0.0)	1.0	0.002	1.000	Excellent
	AW	10	3 (30.0)	7 (70.0)	0 (0.0)	1.0	0.001	1.000	Excellent

BAPQ	AC	8	5 (62.5)	3 (37.5)	0 (0.0)	1.0	0.004	1.000	Excellent
	AW	10	7 (70.0)	1 (10.0)	2 (20.0)	0.8	0.044	0.791	Excellent
CATI	AC	10	6 (60.0)	3 (30.0)	1 (10.0)	0.9	0.001	0.899	Excellent
	AW	9	6 (66.7)	3 (33.3)	0 (0.0)	1.0	0.002	1.000	Excellent
RAADS14	AC	10	7 (70.0)	3 (30.0)	0 (0.0)	1.0	0.001	1.000	Excellent
	AW	10	5 (50.0)	4 (40.0)	1 (10.0)	0.9	0.001	0.899	Excellent
GQASC	AC	10	6 (60.0)	4 (40.0)	0 (0.0)	1.0	0.001	1.000	Excellent
	AW	10	4 (40.0)	5 (50.0)	1 (10.0)	0.9	0.01	0.899	Excellent

Note. AQ: Autism Spectrum Quotient, BAPQ: Broad Autism Phenotype Questionnaire, CATI: The Comprehensive Autistic Trait Inventory, RAADS-14: Ritvo Autism Asperger Diagnostic Scale 14, GQ-ASC: the Girls Questionnaire for Autism Spectrum Condition. I-CVI: item-level content validity index, pc: probability of a chance occurance, k*: modified kappa. Agreement levels are indicated by modified kappa (k*) values: below 0.40 = poor, 0.40–0.59 = fair, 0.60–0.74 = good, and above 0.74 = excellent.
[1] The response options ‘Relevant’ and ‘Somewhat relevant’ were combined into a single ‘Relevant’ category for calculating I-CVI and k*.

Figure A1

Percentage differences in relevance ratings of AQ items between AC and AW

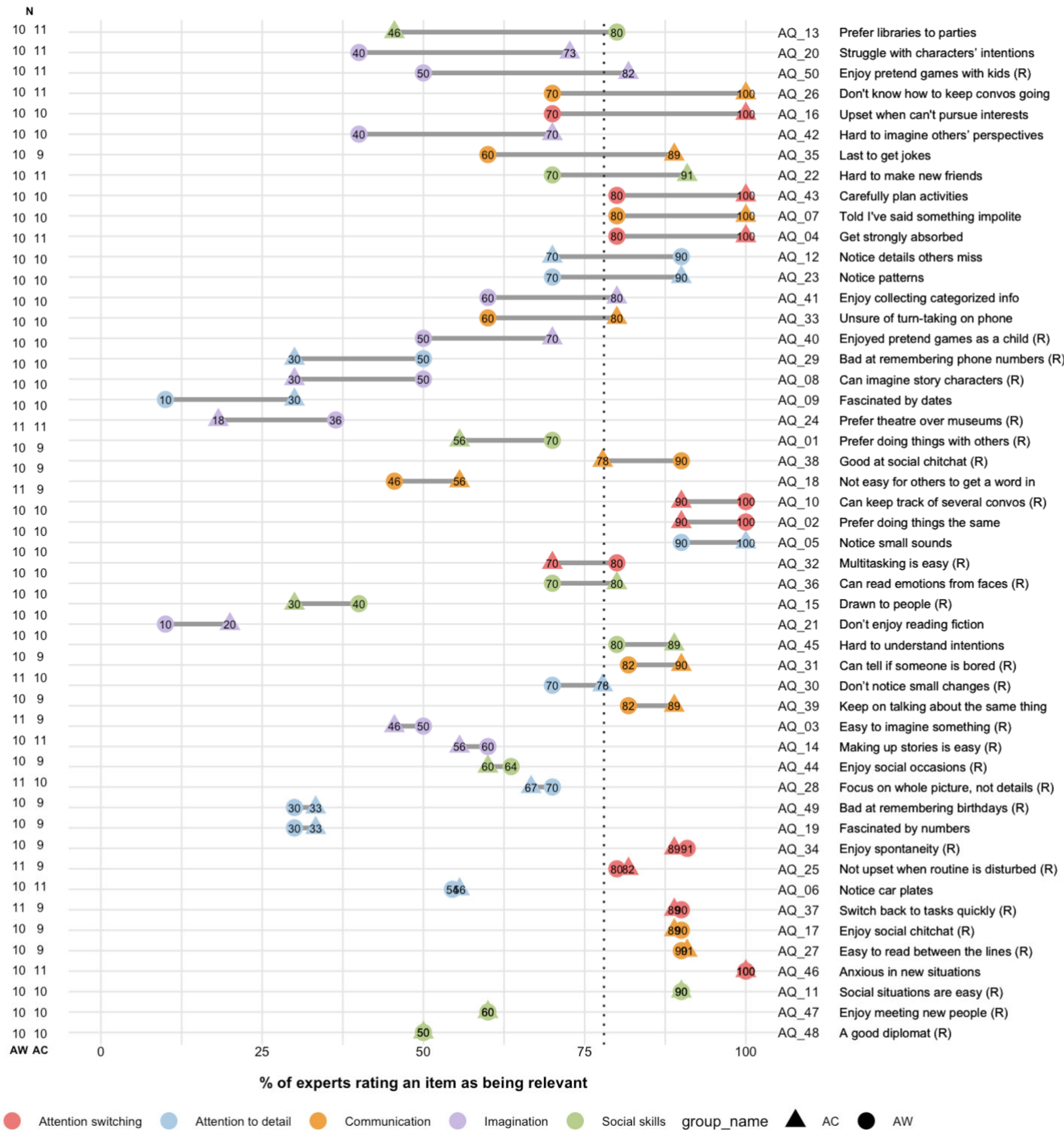

Note. AC: academics and clinicians, AW: autistic women. Dotted vertical line represents I-CVI ≥0.78 ≈ 78%, which corresponds to excellent agreement for modified kappa (Polit et al., 2007). (R) signifies a reverse scored statement.

Figure A2

Percentage differences in relevance ratings of BAPQ items between AC and AW

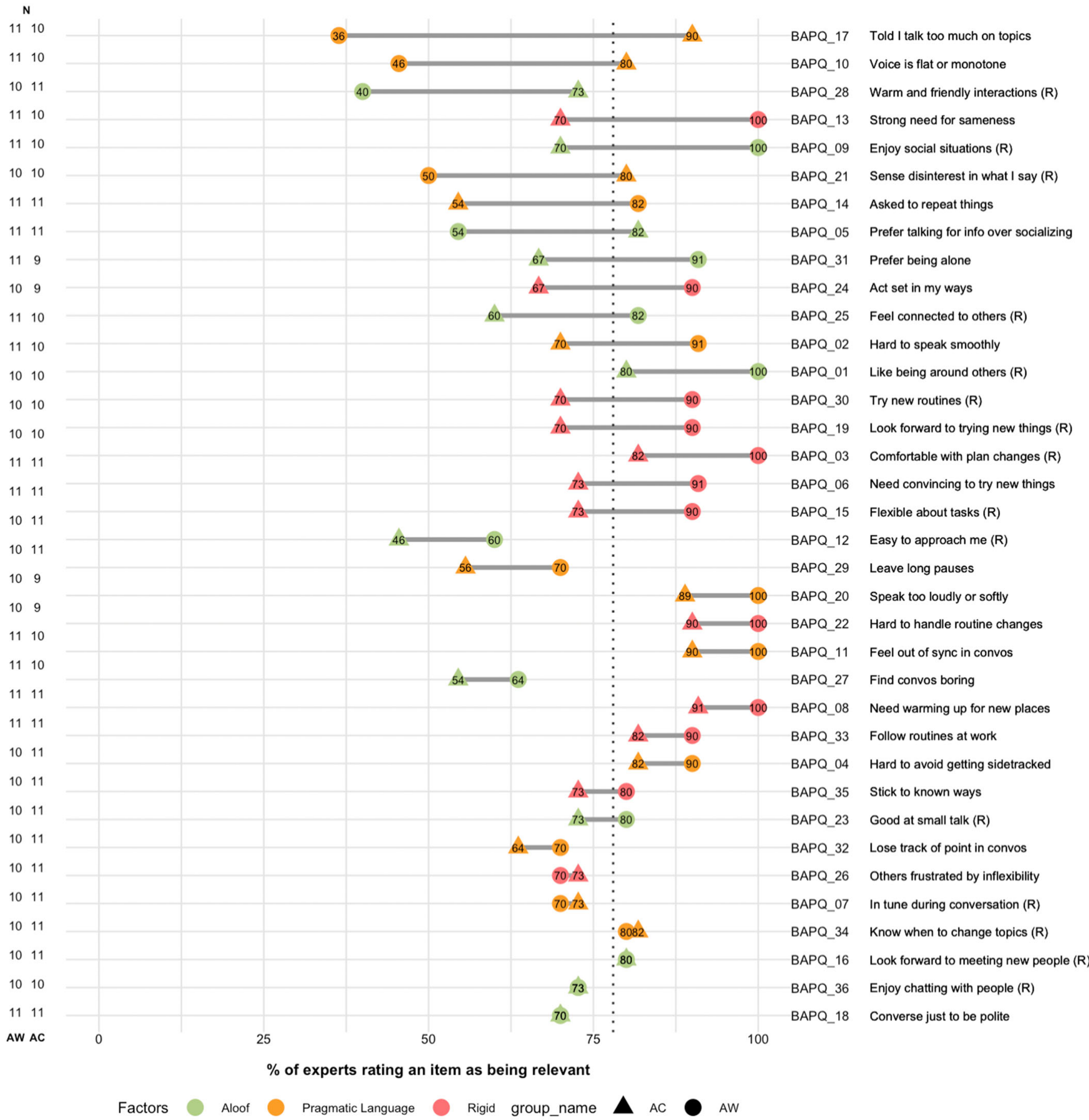

Note. AC: academics and clinicians, AW: autistic women. Dotted vertical line represents I-CVI ≥0.78 ≈ 78%, which corresponds to excellent agreement for modified kappa (Polit et al., 2007). (R) signifies a reverse scored statement.

Figure A3

Percentage differences in relevance ratings of CATI items between AC and AW

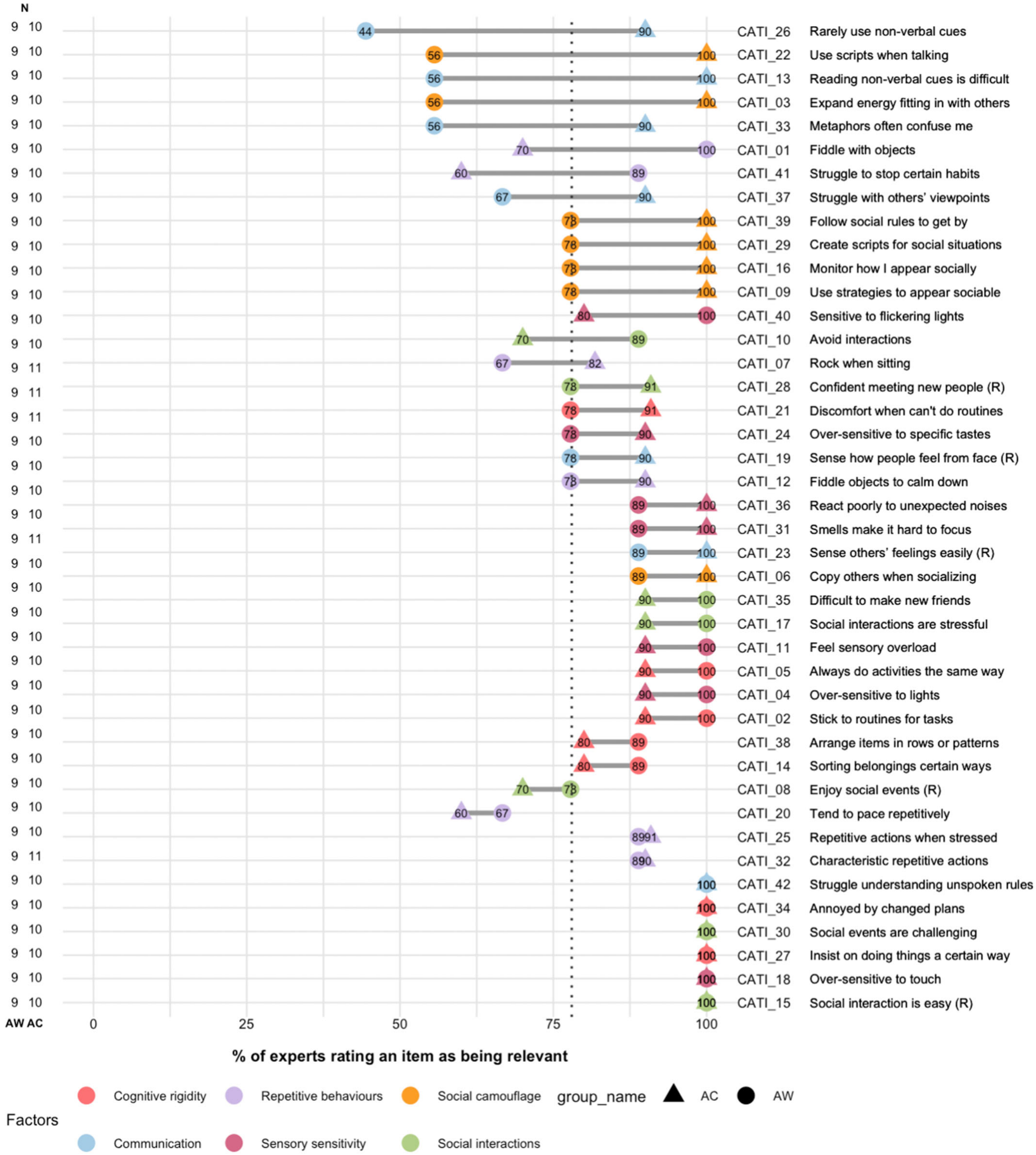

Note. AC: academics and clinicians, AW: autistic women. Dotted vertical line represents I-CVI ≥0.78 ≈ 78%, which corresponds to excellent agreement for modified kappa (Polit et al., 2007). (R) signifies a reverse scored statement.

Figure A4

Percentage differences in relevance ratings of GQ-ASC items between AC and AW

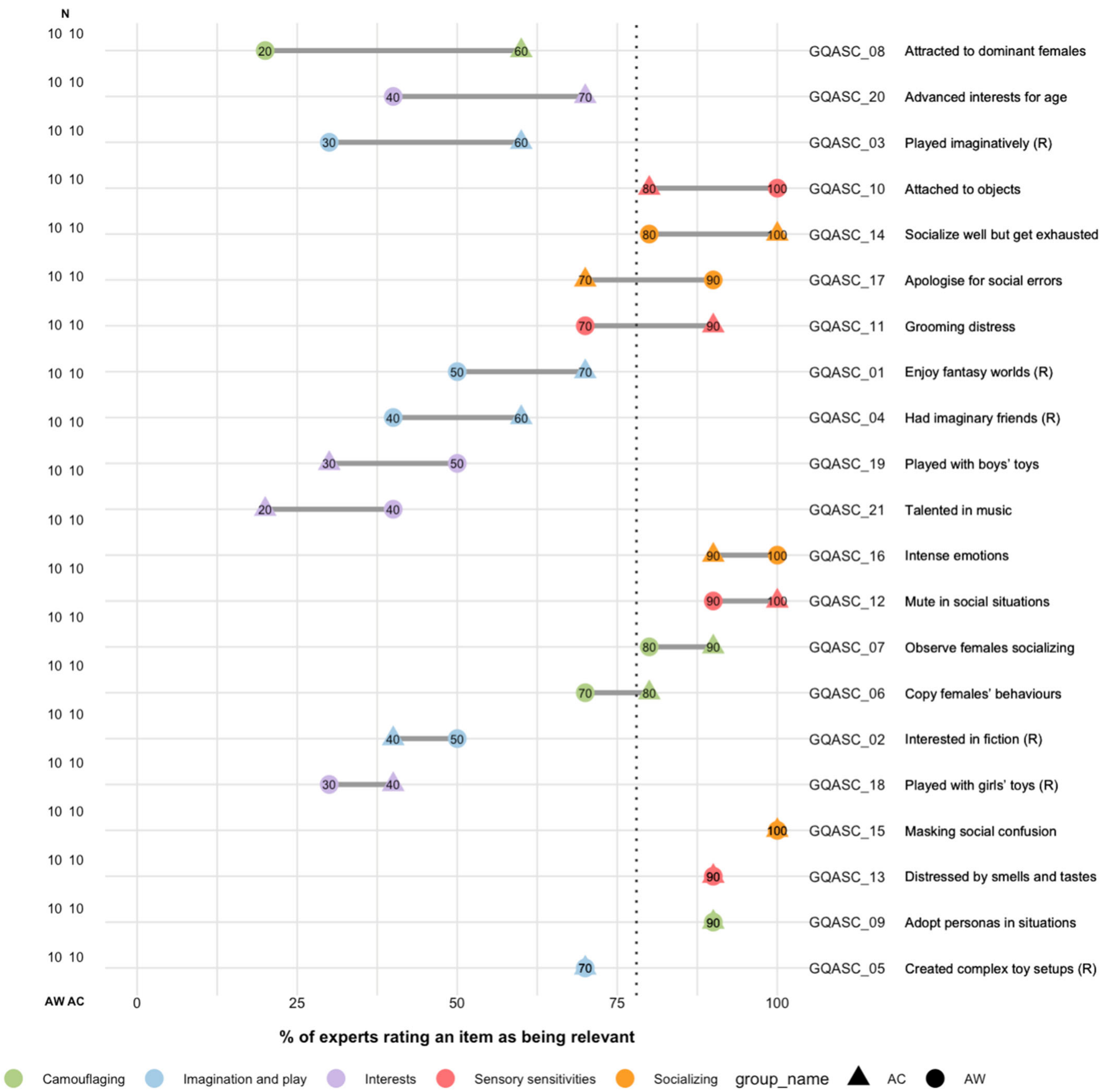

Note. AC: academics and clinicians, AW: autistic women. Dotted vertical line represents I-CVI ≥0.78 ≈ 78%, which corresponds to excellent agreement for modified kappa (Polit et al., 2007). (R) signifies a reverse scored statement.

Figure A5

Percentage differences in relevance ratings of RAADS-14 items between AC and AW

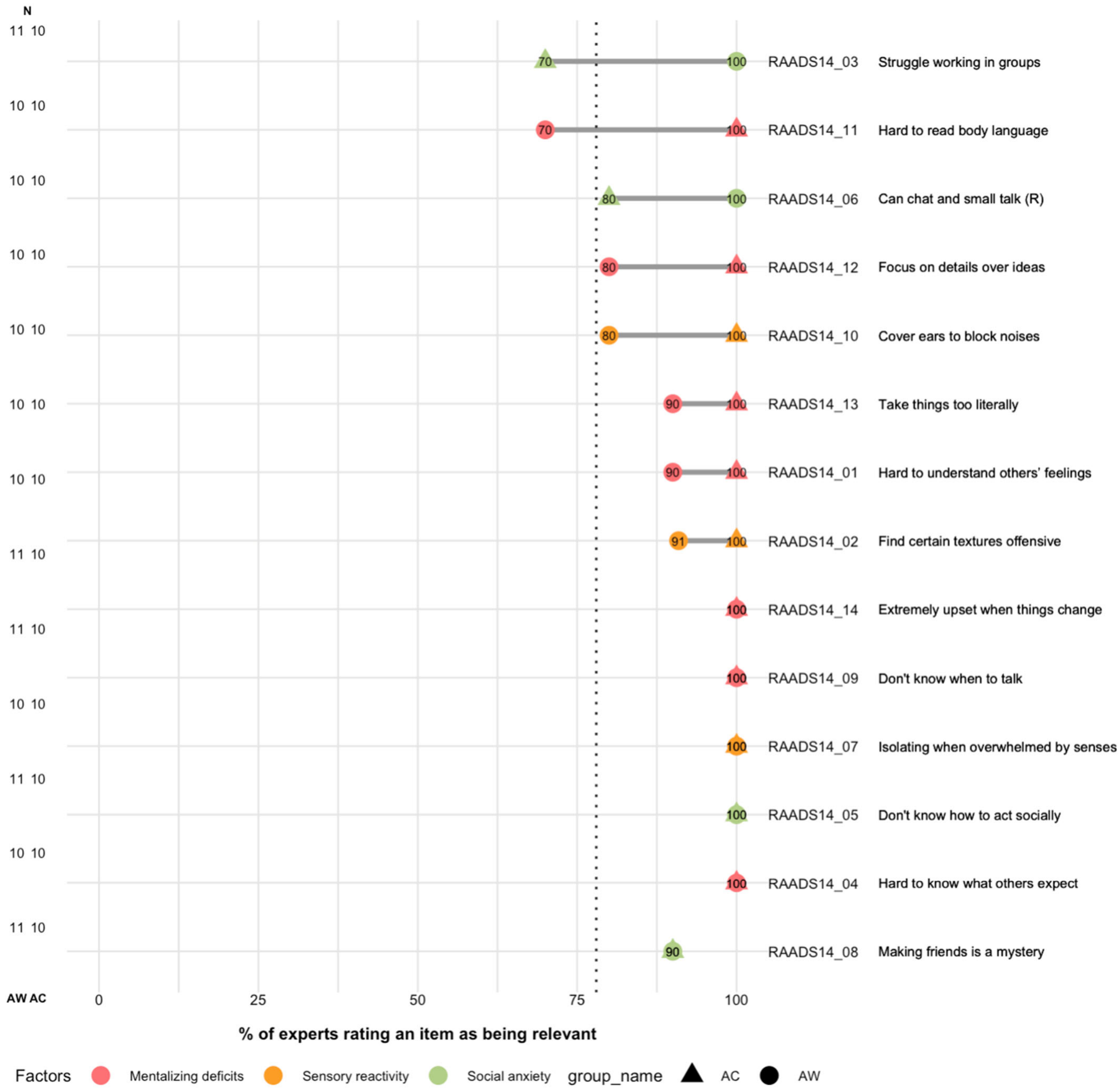

Note. AC: academics and clinicians, AW: autistic women. Dotted vertical line represents I-CVI ≥0.78 ≈ 78%, which corresponds to excellent agreement for modified kappa (Polit et al., 2007). (R) signifies a reverse scored statement.
